# Ambiphilicity of ring-expanded N-heterocyclic carbenes†

**DOI:** 10.1039/d3sc04543a

**Published:** 2024-01-31

**Authors:** François Vermersch, Victor T. Wang, Mehdi Abdellaoui, Rodolphe Jazzar, Guy Bertrand

**Affiliations:** a UCSD-CNRS Joint Research Chemistry Laboratory (IRL 3555), Department of Chemistry and Biochemistry, University of California La Jolla San Diego California 92093-0358 USA rjazzar@ucsd.edu gbertrand@ucsd.edu

## Abstract

N-heterocyclic carbenes, such as imidazole-2-ylidenes and imidazolin-2-ylidenes, the popular class of singlet carbenes introduced by Arduengo in 1991 have not been shown to be ambiphilic owing to the two σ-withdrawing, π-donating amino groups flanking the carbene centre. However, our experimental data suggest that ring-expanded N-heterocyclic carbenes (RE-NHCs), especially the seven and eight membered rings, are significantly ambiphilic. Our results also show that the steric environment in RE-NHCs can become a determining factor for controlling the E–H bond activation.

## Abstract 2

Contrasting with popular beliefs, experimental and theoretical data show that some N-heterocyclic carbenes (NHCs), especially the seven and eight membered rings NHCs, are very ambiphilic.

## Graphical abstract

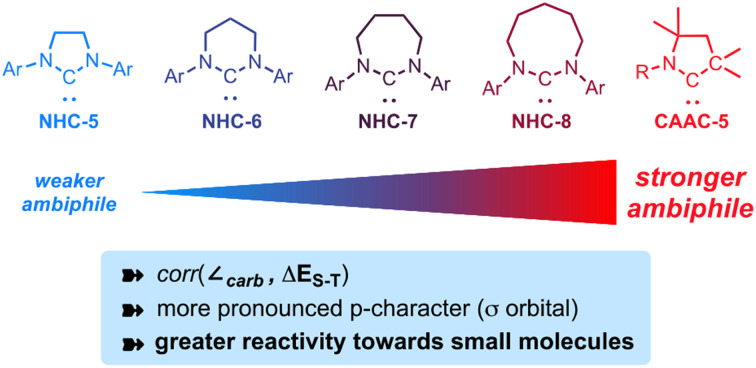

## Introduction

Thanks to growing efforts in main group chemistry, the activation of enthalpically strong bonds and industrially relevant small molecules is no longer restricted to transition-metals.^1^ More than a decade ago, our group discovered that cyclic (alkyl)(amino)carbenes (CAAC-5),^2,3^ a class of highly ambiphilic carbenes, could react with carbon monoxide,^4^ H_2_,^5^ NH_3_ (ref. 5) and P_4_.^6^ More recently, it has been shown that CAAC-5s not only activate a variety of bonds (C–H, Si–H, B–H…)^7^ but also promote catalytic reactions.^8^ In comparison, imidazole-2-ylidenes^9^ and imidazolin-2-ylidenes,10 the classical N-heterocyclic carbenes (NHC-5s), are much less ambiphilic due to their two π-donating amino substituents. Consequently, they are reluctant to activate small molecules, as illustrated by their lack of reactivity with CO.^11,12^ Much less studied than NHC-5s are the so-called ring-expanded N-heterocyclic carbenes (RE-NHCs).^13^ Herein we compare the ambiphilic nature of NHC-5 with RE-NHCs (−6,^13*a*^ −7 (ref. 14) and −8 (ref. 13c)) and CAAC-5 through DFT calculations and their reactivity with small molecules.

## Results and discussion

Compared to NHC-5s, RE-NHCs display a larger N–C–N bond angle (∠_carb_) which imposes greater steric constraint when used as a ligand for transition metals, a feature used to enhance catalytic activity.^15^ Arguably less emphasized, is the larger carbene bond angle, which increases the p-character of the lone pair, and thus the energy level of the HOMO.^16^ Comparatively, the LUMO is less affected since ring expansion does not significantly disrupt the planarization of the α-amino fragments, which leaves the mesomeric stabilization of the p_π_ orbital by the nitrogen lone pairs nearly identical.

**Scheme 1:**
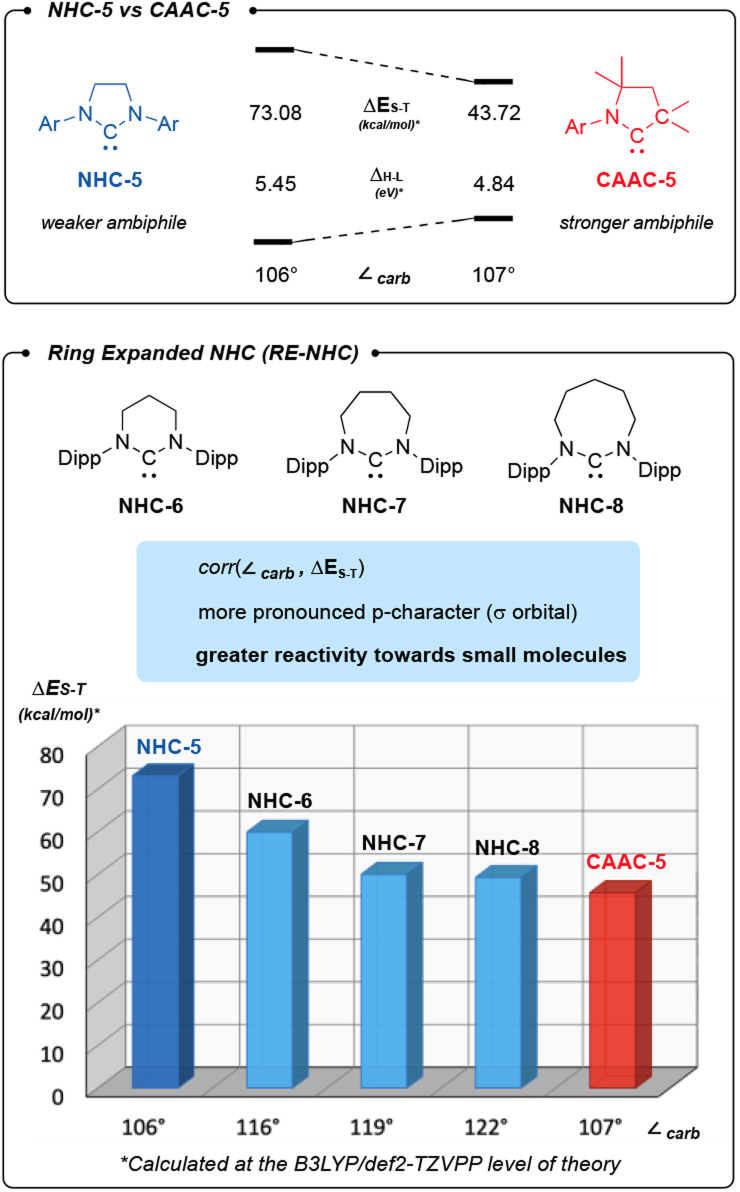
CAAC-5 is more ambiphilic than NHC-5. NHC ambiphilicity is improved in ring-expanded NHC (ReNHCs) as shown by their decreasing singlet–triplet gap (Δ*E*_S–T_).

The ambiphilicity of a carbene can be estimated computationally by considering the singlet–triplet gap (Δ*E*_S–T_) (Scheme 1). As expected, our calculations indicate a correlation between the ring size and ambiphilicity of a carbene. Interestingly, the data also suggests that the ambiphilicity of NHC-7 and NHC-8 approaches that of CAAC-5.

**Scheme 2:**
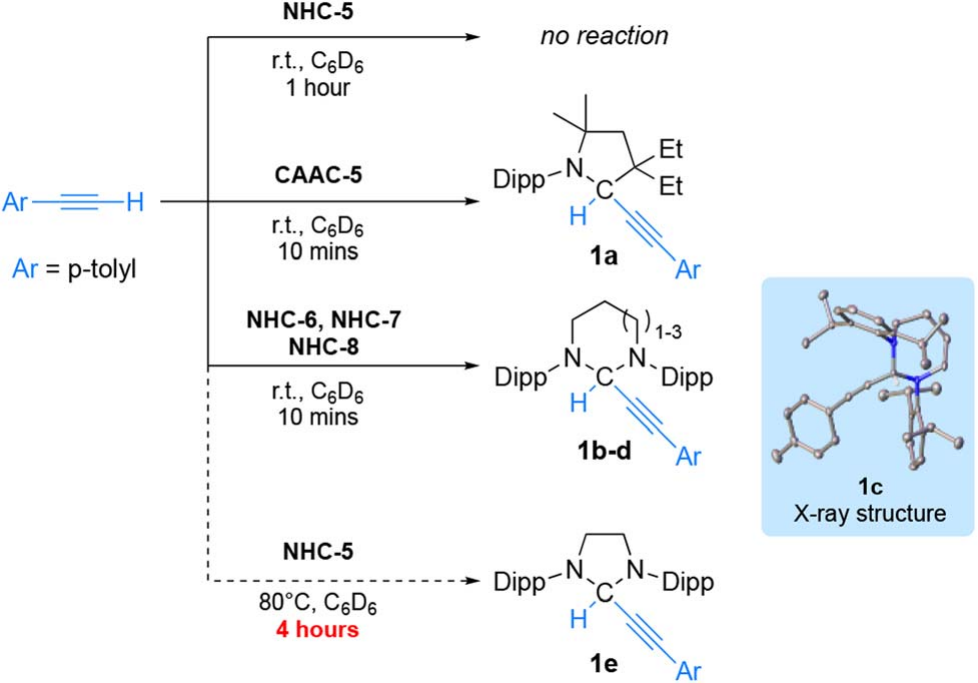
Reactivity of NHC-5–8 and CAAC-5 with *p*-tolylacetylene.

To compare experimentally the ambiphilicity of NHCs with that of CAAC-5, we first considered the activation of sp-hybridized CH bonds which has been reported with CAACs,^17^ but seldomly described with NHCs (one example has been reported using acetylene gas).^18^ We first investigated the reaction of *p*-tolylacetylene [p*K*_a_ (DMSO) = 28.8 *vs.* 25 for acetylene] with NHC-5 at room temperature in benzene solution (Scheme 2). In this case, no reaction was observed within 1 hour. In marked contrast, using CAAC-5 the oxidative addition product 1a was quantitatively obtained within minutes. Under the same conditions a rapid and clean reaction was also observed with NHC-6^13*a*^NHC-7^15^ and NHC-8^13*c*^ giving adducts 1b–d as shown by characteristic ^1^H NMR signals at 6.04, 5.87 and 5.64 ppm, and ^13^C NMR signals at 72.3, 73.9 and 77.1 ppm, respectively. The structure of adduct 1c (from NHC-7) was confirmed by X-ray crystallography. Because of the significant difference in reactivity observed between NHC-5 and the RE-NHCs, we re-evaluated the reaction of NHC-5 with *p*-tolylacetylene and observed very slow conversion to adduct 1e upon performing the reaction at 80 °C for 4 hours.

These initial results prompted us to search for more challenging molecules to activate. Examples of stable carbenes reacting with isonitriles to afford ketenimines are scarce.

**Scheme 3:**
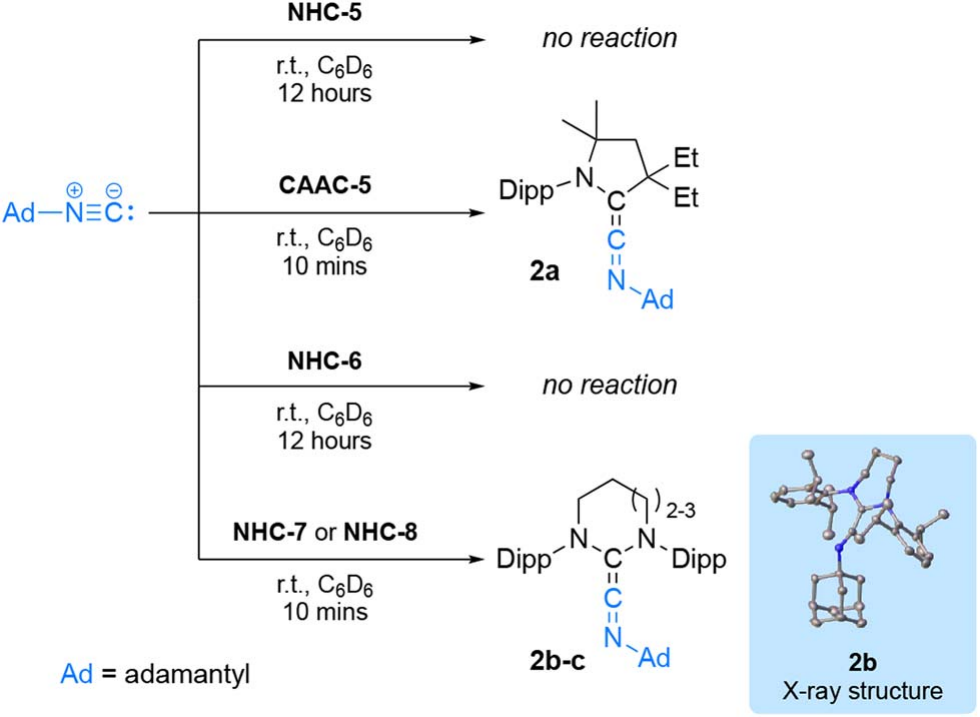
Reactivity of NHC-5–8 and CAAC-5 with adamantyl isocyanide.

They only include the anti-Bredt NHC^19^ and diamidocarbenes (DAC) 20 thanks to their enhanced electrophilicity resulting from reduced donation of the nitrogen lone-pair into the empty p-type orbital of the carbene carbon. Curious to probe the reactivity of RE-NHCs, we considered their reactivity and that of NHC-5 or CAAC-5 with adamantyl isocyanide (Scheme 3). CAAC-5 cleanly afforded the ketenimine 2a within minutes, while no reaction occurred with NHC-5 after 12 hours at room temperature in benzene solution.^21^ This result contrasts with CAAC-5 which cleanly afforded the ketenimine 2a within minutes. With NHC-6, no reaction was observed even after 12 hours. However, with NHC-7 and NHC-8, the quantitative formation of compound 2b and 2c, was observed after 10 minutes, as evidenced by the diagnostic ^13^C NMR signal for the central carbon of ketenimines at 216.9 ppm and 211.5 ppm, respectively. We confirmed the structure of compound 2b by X-ray crystallography. Interestingly, the solid-state structure of 2b revealed a pronounced bent geometry (C_NHC_–C–N angle: 158.5°) compared to that of diamido cyclohexylketenimine (C_DAC_–C–N angle: 173.8°)^20*b*^ with a longer C_NHC_–C_ket_ bond (133.8 pm *vs.* 129.7 pm for DAC). This observation indicates that NHC-7 is less electrophilic than DAC.

**Scheme 4:**
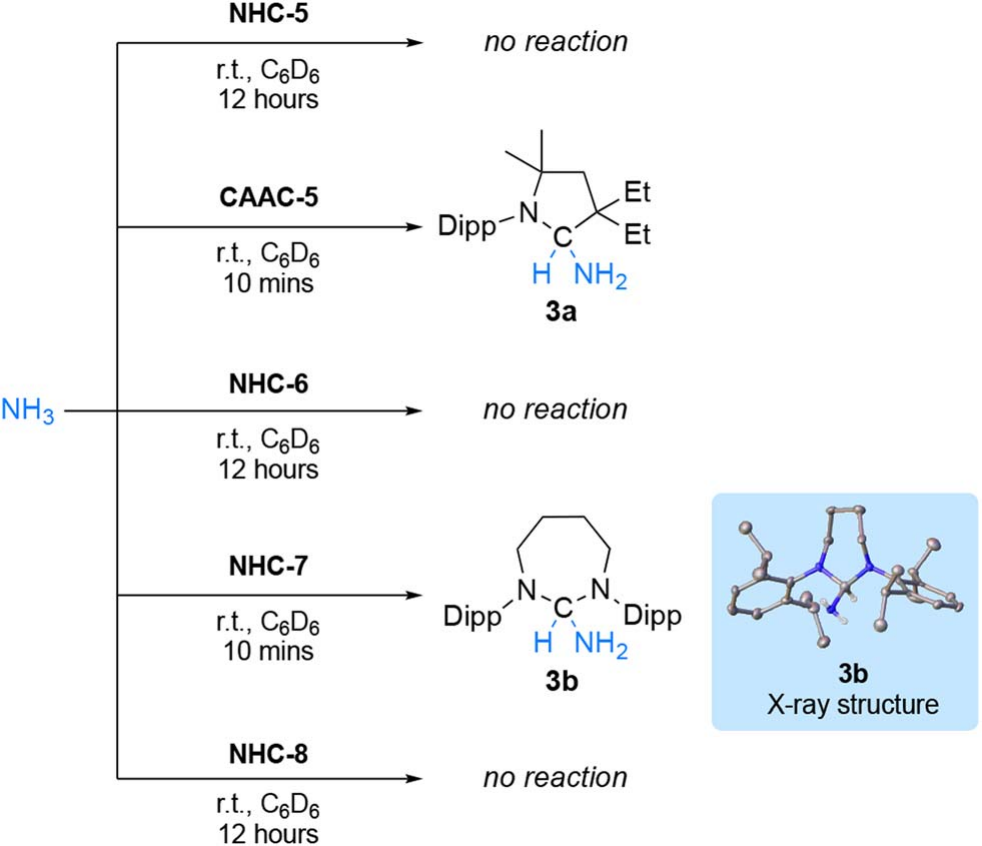
Reactivity of NHC-5–8 and CAAC-5 with ammonia.

Collectively, the reactions with terminal alkynes and isocyanides suggest that the ambiphilicity of the carbenes is in the order NHC-5 < NHC-6 < NHC-7 < NHC-8 < CAAC-5, which is in agreement with their singlet–triplet gap. To deconvolute these results further, we wondered if RE-NHCs, notwithstanding their lower electrophilicity could compare with CAAC-5 in the activation of ammonia.^5^ Under 2 atmospheres of NH_3_, no reaction occurred with NHC-5, which was expected since several diaminocarbenes have even been generated in liquid ammonia.^22^ (Scheme 4). In agreement with literature precedent,^5^ under the same conditions, CAAC-5 rapidly led to the ammonia adduct 3a. Switching to RE-NHCs, no reaction was observed with NHC-6 despite prolonged reaction time, while NHC-7 led to the clean formation of product 3b with distinctive ^1^H and ^13^C NMR signals at *δ* = 5.25 ppm and 85.8 ppm, respectively. This result was confirmed by single crystal X-ray diffraction. However, to our surprise, no reaction was observed with NHC-8.

**Scheme 5:**
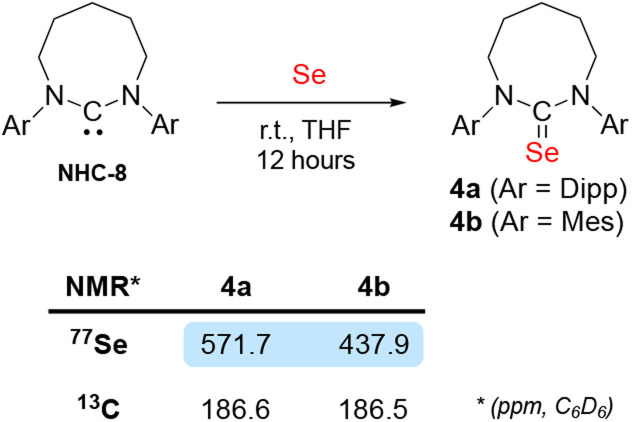
Selenium adducts of Dipp- and Mes-substituted NHC-8.

We previously reported that the steric environment of CAAC-5 is a determining factor in controlling the reversibility of E–H bond activation (E = N–H, P–H).^8^ Compared to CAAC-5 (∠_carb_ = 106°), NHC-8 is more sterically constrained around the carbene carbon due to its large N–C_NHC_–N bond angle (∠_carb_ = 122°).^13*c*^ We hypothesized this could explain its lack of reactivity with ammonia despite favourable electronics. To probe this hypothesis, we prepared the *N*-Mesityl (-Mes) substituted NHC-8 (^Mes^NHC-8) since its steric profile is significantly smaller than that of the Dipp-substituted NHC-8. This is apparent from the solid state structures, when considering the steric maps (see ESI for details†) and percent buried volumes (%*V*_bur_^23^) around the carbene carbon. NHC-8 (80.1%) compared to ^Mes^NHC-8 (77.2%) which is closer to that of NHC-7 (78.4.%). The larger steric hindrance is also apparent in solution when considering the unusual ^77^Se NMR downfield shift of the NHC-8-Se adduct 4a (571.1 ppm) compared to ^Mes^NHC-8-Se adduct 4b (437.9 ppm) (Scheme 5). Indeed, ^77^Se NMR is a spectroscopic marker for highlighting non-classical bonding (NCB) interactions between pendant *N*-Dipp substituents and the selenium atom.^24^ Note that when comparing the reactivity of *N*-tolyl and *N*-Dipp 8-membered NHCs with silver chloride, Cavell and co-workers discovered that in very large ring NHCs the steric environment provided by *N*-Dipp substituents can become so overwhelming that it prevents coordination.^13*c*^

**Scheme 6:**
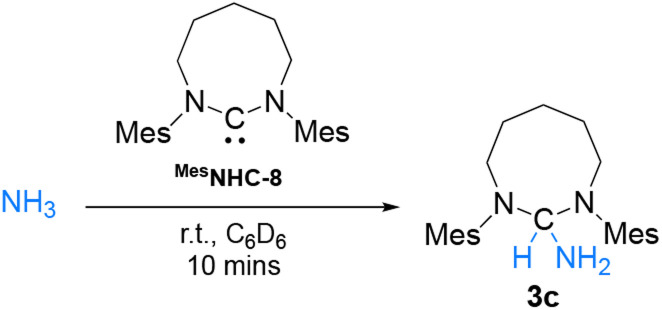
Reactivity of ^Mes^NHC-8 with ammonia.

**Scheme 7:**
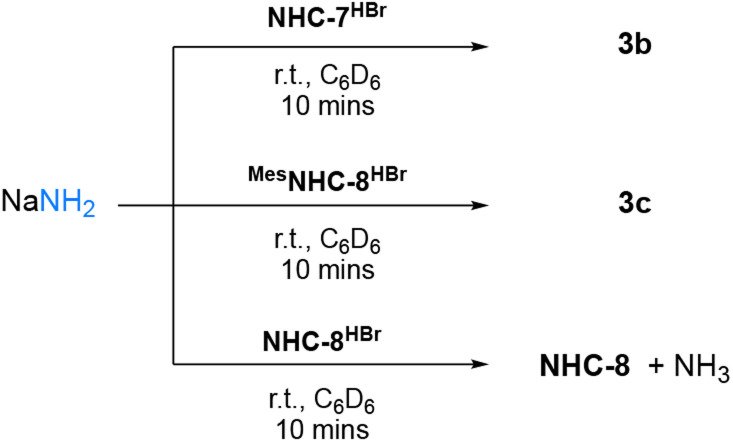
Reactivity of NHC-7, ^Mes^NHC-8 and NHC-8 conjugate acid salts with NaNH_2_.

Having confirmed that ^Mes^NHC-8 is less sterically hindered than NHC-8 and even NHC-7, we evaluated its reactivity towards ammonia. Gratifyingly, rapid formation of the corresponding ammonia adduct was observed when performing the reaction in C_6_D_6_ under 2 atmospheres of NH_3_ (Scheme 6). To confirm these results, we also investigated the reactivity of the corresponding imidazolium salts with sodium amide which provided the expected adducts *via* nucleophilic addition of NH_2_^−^ (Scheme 7). Note that under these conditions, reaction of NHC-8^HBr^ with NaNH_2_ afforded the free NHC-8 and ammonia. Overall, these results suggest that for 8-membered ring NHCs, the activation of ammonia is controlled by steric parameters and possibly reversible.

## Conclusions

Imidazole-2-ylidenes^9^ and imidazolin-2-ylidenes,^10^ the popular class of singlet carbenes introduced by Arduengo, have not proven to be ambiphilic owing to the two σ-withdrawing, π-donating amino groups stabilizing the carbene centre. However, our experimental data demonstrate that ring-expanded N-heterocyclic carbenes, NHC-7s and NHC-8s, belong to the class of ambiphilic carbenes. Our results also show that the steric environment in RE-NHCs can become a determining factor for controlling the E–H bond activation. We anticipate these results will have far reaching implications in the design and applications of large ring singlet carbene skeletons.

## Data availability

All the data available has been provided in the ESI.†

## Author contributions

F. V. and R. J. conceptualized this work. F. V., V. T. W. and M. A. performed the synthetic work. R. J. performed X-ray diffraction analysis. The manuscript was written by R. J. and G. B. and reviewed by all the authors. R. J. and G. B. guided the project.

## Conflicts of interest

There are no conflicts to declare.

## Supplementary Material

SC-015-D3SC04543A-s001

SC-015-D3SC04543A-s002
