# Supplementary material for: Ambiphilicity of ring-expanded N-heterocyclic carbenes
Source: Chem Sci. 2024 Jan 31;15(10):3707–10. doi: 10.1039/d3sc04543a (PMC10915854; doi:10.1039/d3sc04543a)
Supplement: SC-015-D3SC04543A-s001 [file SC-015-D3SC04543A-s001.pdf]

Electronic Supplementary Material (ESI) for Chemical Science.
This journal is © The Royal Society of Chemistry 2024

Supplementary Information for

Ambiphilicity of Ring-Expanded *N*-Heterocyclic Carbenes

Francois L. Vermersch,[#a] Victor Wang,[#a] Mehdi Abdellaoui,[a] Rodolphe Jazzar,*[a] Guy Bertrand*[a]

[a] UCSD–CNRS Joint Research Laboratory (UMI 3555), Department of Chemistry and Biochemistry, University of California, San Diego, La Jolla, CA 92093–0343, United States.

e-mail: rjazzar@ucsd.edu; gbertrand@ucsd.edu

Table of Contents

I. GENERAL CONSIDERATIONS 3
II. SYNTHESIS AND CHARACTERIZATION OF IMINIUMS AND CARBENES 3
A. Literature procedures 3
B. Characterizations 3
B.1. Carbene Iminium Precursors 3
B.2. Carbene Iminium Precursors NMR Spectra 4
B.3. Free Carbenes 9
B.4. Free Carbene NMR Spectra 10
III. COMPARISON OF REACTIVITY BETWEEN NHC-5,6,7,8 15
A. Reactions Between NHC and Alkynes 15
A.1. NHC-5 with *p*-Tolylacetylene (1e) 15
A.2. NHC-6 with *p*-Tolylacetylene (1b) 16
A.3. NHC-7 with *p*-Tolylacetylene (1c) 18
A.4. NHC-8 with *p*-Tolylacetylene (1d) conformers 19
B. Reactions Between NHC and Isocyanides 21
B.1. NHC-7 with Adamantyl Isocyanide (2b) 21
B.2. NHC-8 with Adamantyl Isocyanide (2c) 22
C. Reactions Between NHC and Ammonia 24
C.1. NHC-7 with Ammonia (3a) 24
C.2. ^{Mes}NHC-8 with Ammonia (3c) 25
D. Synthesis of NHC-Selenium Adducts 27
D.1. NHC-8.HBr with Selenium (4a) 27
D.2. ^{Mes}NHC-8.HBr with Selenium (4b) 29
E. Reactions Between NHC.HBr and $NaNH_2$ 31
E.1. NHC6.HBr and $NaNH_2$ with *dibenzo*-18-crown-6. 31
E.2. NHC6.HBr and $NaNH_2$ with *dibenzo*-18-crown-6. 32
E.3. NHC8.HBr and $NaNH_2$ with *dibenzo*-18-crown-6. 33
E.4. NHC8.HBr and $NaNH_2$ with *dibenzo*-18-crown-6. 34
IV. X-RAYS STRUCTURES 35
IV.1 X-Ray crystal structure determination 35
IV.2 X-Ray crystal structure determination for Compound 1c (CCDC 2291449) 35
IV.3 X-Ray crystal structure determination for Compound 1d (CCDC 2291455) 37
IV.4 X-Ray crystal structure determination for Compound 2b (CCDC 2291453) 39
IV.5 X-Ray crystal structure determination for Compound 2c (CCDC 2291452) 41
IV.6 X-Ray crystal structure determination for Compound 3b (CCDC 2291450) 43
IV.7 X-Ray crystal structure determination for Compound 4a (CCDC 2291454) 45
IV.8 X-Ray crystal structure determination for Compound 4b (CCDC 2291451) 47
X. COMPUTATIONAL DATA 49

I. General Considerations

All manipulations were performed using standard glovebox and Schlenk techniques. Glassware was dried in an oven overnight at 150 °C or flame-dried, and solvent were dried and degassed before use. Benzene, THF, diethyl ether, and n-pentane were freshly distilled over Na metal. Hexanes, dichloromethane, and chloroform were freshly distilled over CaH_2.

NMR Characterization: Deuterium-labeled solvents were purchased from Cambridge Isotope Laboratories. NMR: Multinuclear NMR data were recorded on a Varian INOVA 500 MHz or a Bruker Avance 300 MHz at UCSD. Chemical shifts (δ) are reported in parts per million (ppm) and are referenced to residual solvent signals (^{1}H, ^{13}C). Coupling constants J are given in hertz (Hz). NMR multiplicities are abbreviated as follows: s = singlet, d = doublet, t = triplet, q = quartet, sext = sextet, sept = septet, m = multiplet, br = broad. All spectra were recorded at 298 K unless otherwise noted.

II. Synthesis and Characterization of Iminiums and Carbenes

A. Literature procedures

Iminium precursors and the corresponding carbenes were prepared according to known literature procedures: **NHC-5,**[1] **NHC-6**,[2] **NHC-7**,[3] and **NHC-8** and ^{Mes}**NHC-8**[4].

B. Characterizations

B.1. Carbene Iminium Precursors

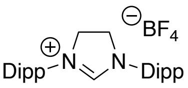

NHC-5^H

NHC-5.HBF$_4$ (NHC-5^H): ^{1}H NMR (300 MHz, $CDCl_3$) δ = 8.49 (s, 1H), 7.44 (t, 2H, J=7.8Hz), 7.25 (d, 4H, J=7.8Hz), 4.80 (s, 4H), 2.97 (sept, 4H, J=6.9Hz), 1.36 (d, 12H, J=6.9Hz), 1.23 (d, 12H, J=6.9Hz) ^{13}C{^{1}H} NMR (125 MHz, $CDCl_3$) δ = 158.8, 146.5, 131.9, 129.5, 125.3, 55.4, 29.0, 25.3, 23.5.

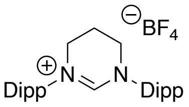

NHC-6^H

NHC-6.HBF$_4$ (NHC-6^H): ^{1}H NMR (500 MHz, $CDCl_3$) δ = 7.56 (s, 1H), 7.42 (t, 2H, J=7.8Hz), 7.24 (d, 4H, J=7.8Hz), 4.22 (t, 4H), 3.01 (dt, 4H), 2.74 (br m, 2H), 1.35 (d, 12H, J=6.8Hz), 1.20 (d, 12H, J=6.8Hz) ^{13}C{^{1}H} NMR (125 MHz, $CDCl_3$) δ = 153.3, 146.1, 136.1, 131.6, 125.4, 48.9, 28.7, 24.7, 24.6, 19.2.

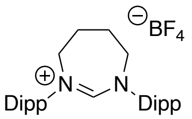

NHC-7^H

NHC-7.HBF$_4$ (NHC-7^H): ^{1}H NMR (500 MHz, $CDCl_3$) δ = 7.42 (t, 2H, J=7.8Hz), 7.30 (s, 1H), 7.25 (d, 4H, J=7.8Hz), 4.68 (br s, 4H), 3.21 (dt, 4H, J=6.8Hz), 2.64 (br s, 4H), 1.39 (d, 12H, J=6.8Hz), 1.24 (d, 12H, J=6.8Hz) ^{13}C{^{1}H} NMR (125 MHz, $CDCl_3$) δ = 157.5, 145.3, 139.2, 131.3, 125.7, 56.2, 28.8, 24.8, 24.6, 24.4.

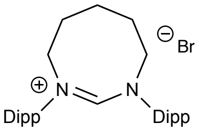

NHC-8^H

NHC-8.HBr (NHC-8^H): ^{1}H NMR (400 MHz, $CDCl_3$) δ = 7.47 (s, 1H), 7.36 (t, 2H, J=7.7Hz), 7.20 (d, 4H, J=7.7Hz), 4.83 (br s, 4H), 3.24 (dt, 4H, J=6.8Hz), 2.30 (br s, 4H), 2.12 (br s, 2H), 1.34 (d, 12H, J=6.8Hz), 1.21 (d, 12H, J=6.8Hz) ^{13}C{^{1}H} NMR (100 MHz, $CDCl_3$) δ = 157.03, 144.79, 141.05, 130.75, 125.55, 55.13, 28.74, 27.67, 25.17, 24.94, 21.46.

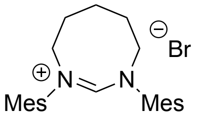

^{Mes}NHC-8^H

^{Mes}NHC-8.HBr (^{Mes}NHC-8^H): ^{1}H NMR (400 MHz, $CDCl_3$) δ = 7.39 (s, 1H), 6.93 (s, 4H), 4.81 (m, 4H), 2.41 (s, 12H), 2.29 (m, 4H), 2.26 (s, 6H), 2.13 (m, 2H), 1.34 (d, 12H, J=6.8Hz), 1.21 (d, 12H, J=6.8Hz) ^{13}C{^{1}H} NMR (100 MHz, $CDCl_3$) δ = 158.8, 141.9, 140.1, 133.7, 130.5, 53.9, 28.4, 21.0, 20.9, 18.9.

B.2. Carbene Iminium Precursors NMR Spectra

NHC-5.HBF$_4$ (NHC-5^H)

^{1}H

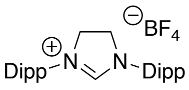

NHC-5^H

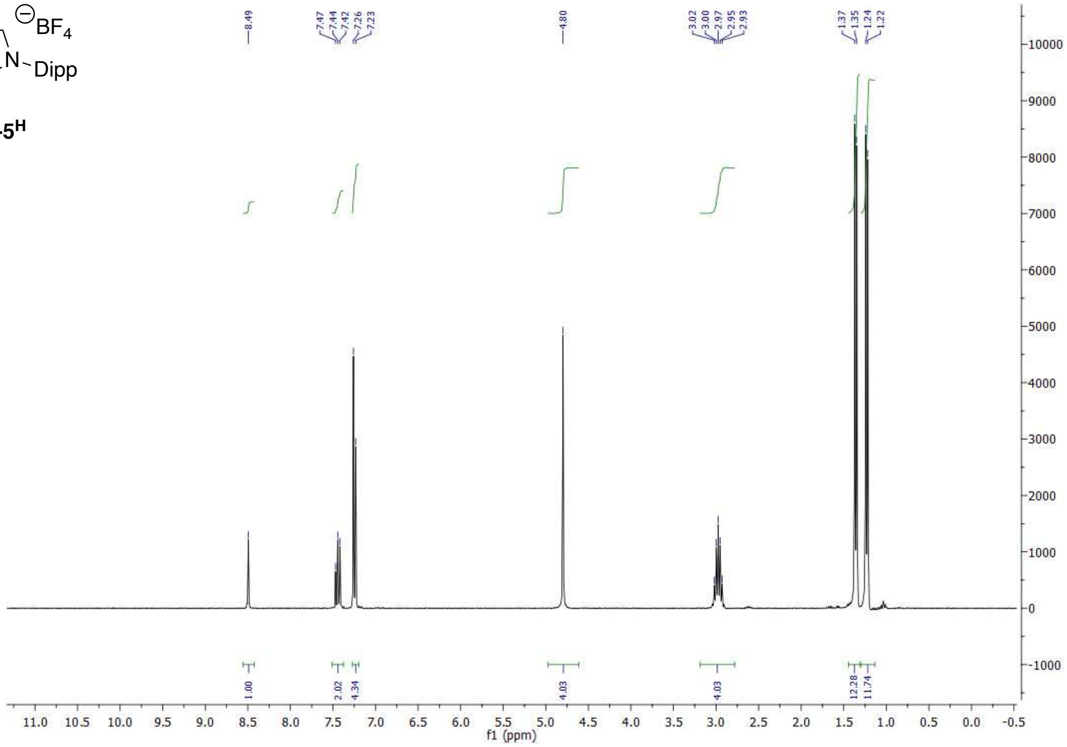

^{13}C{^{1}H}

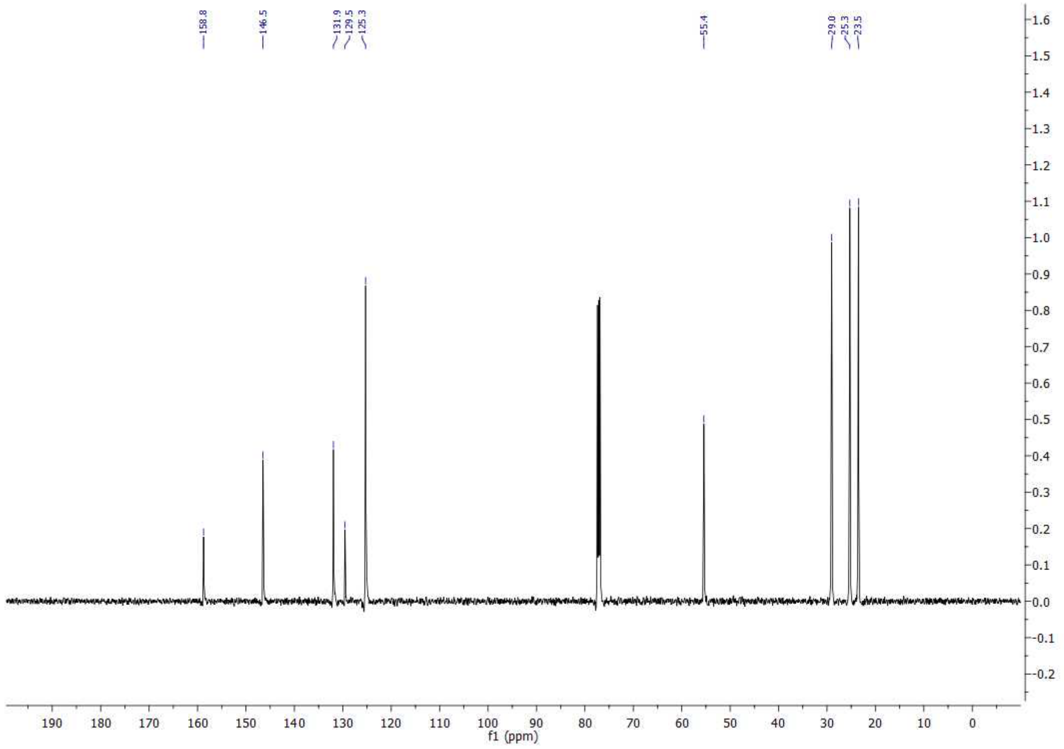

NHC-6.HBF$_4$ (NHC-6^H)

^{1}H

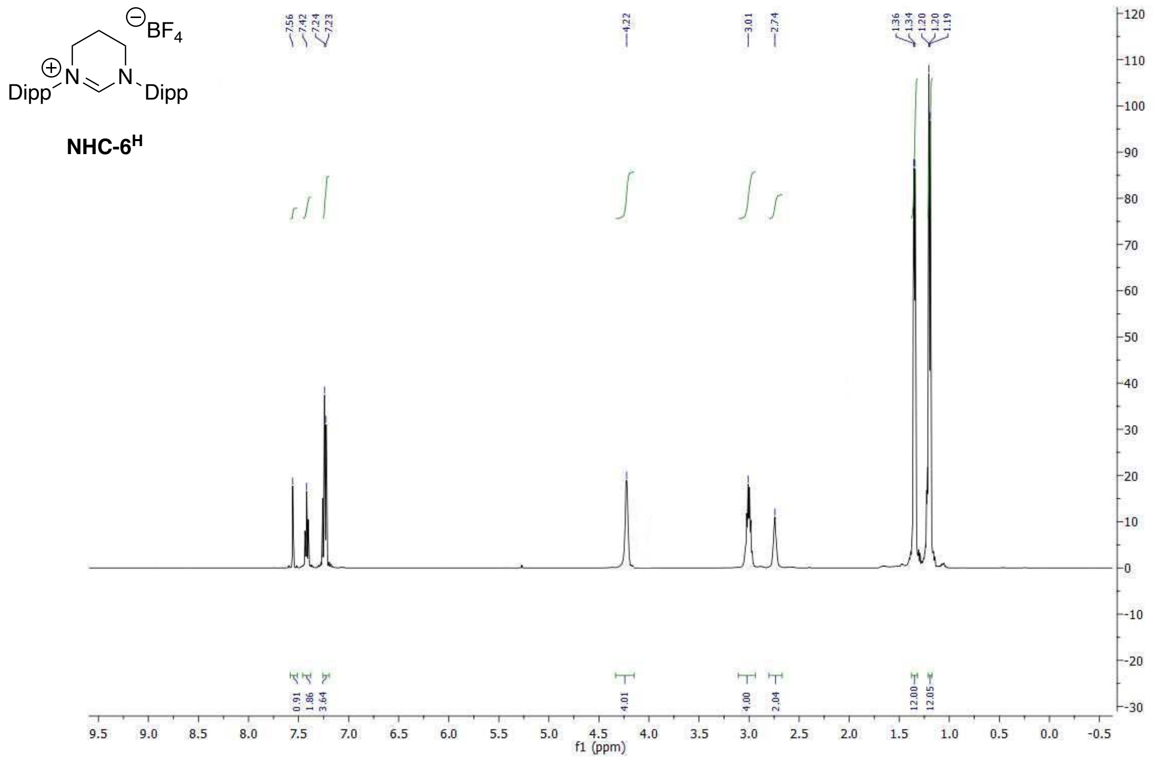

^{13}C{^{1}H}

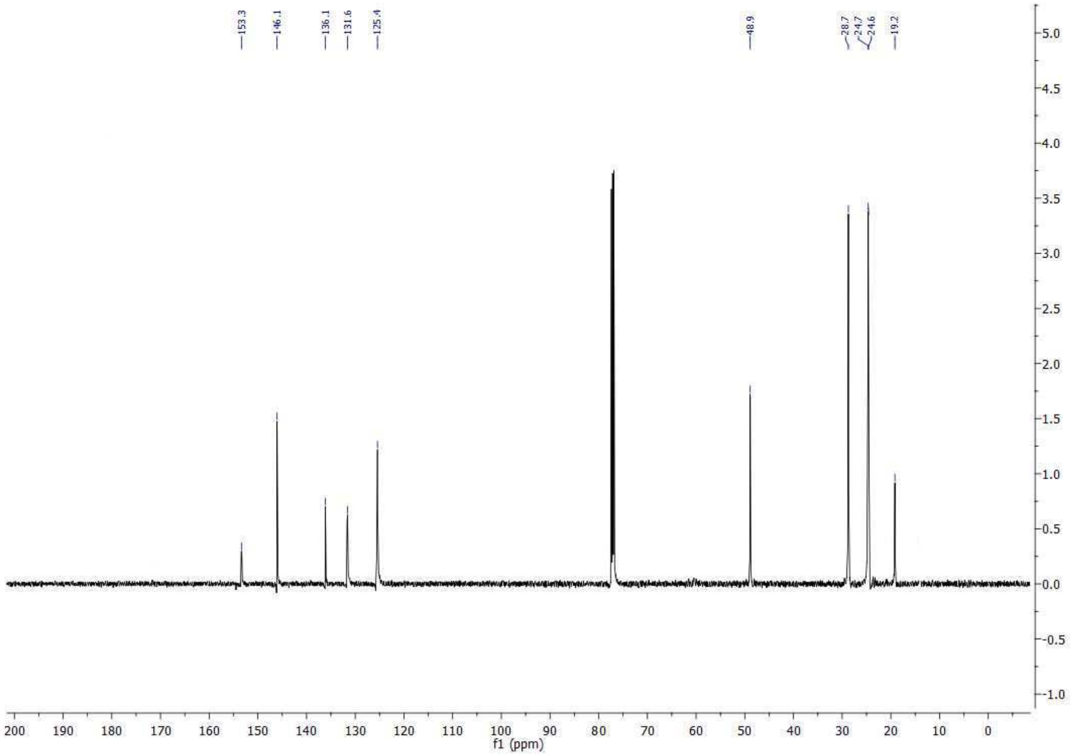

NHC-7.HBF$_4$ (NHC-7^H)

^{1}H

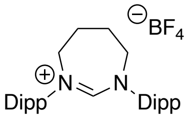

NHC-7^H

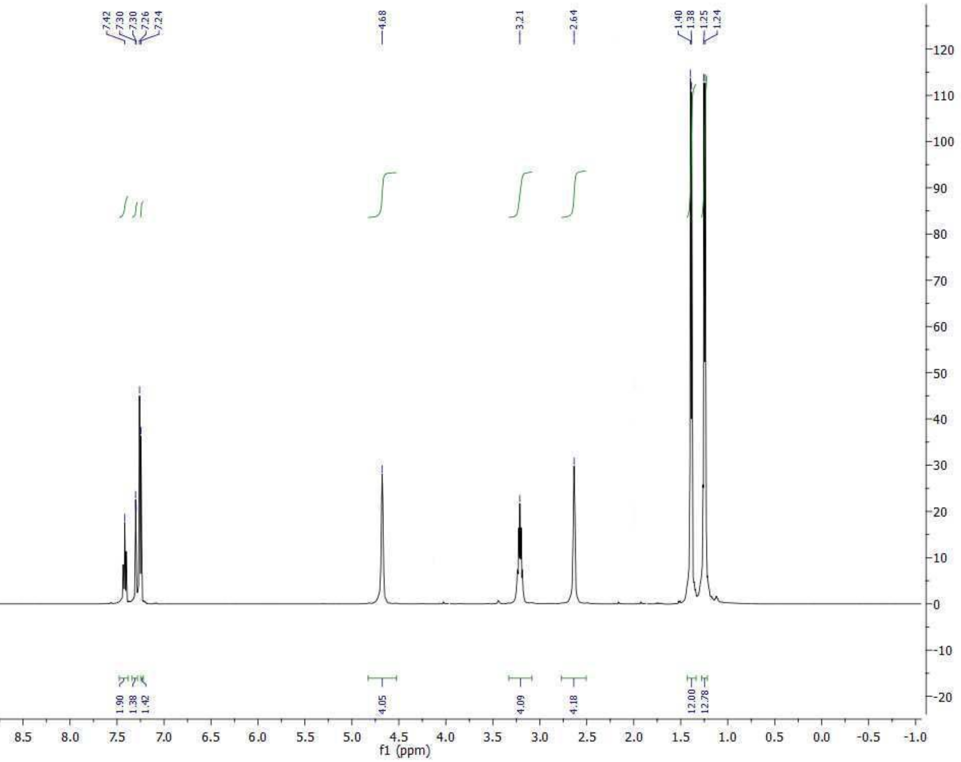

^{13}C{^{1}H}

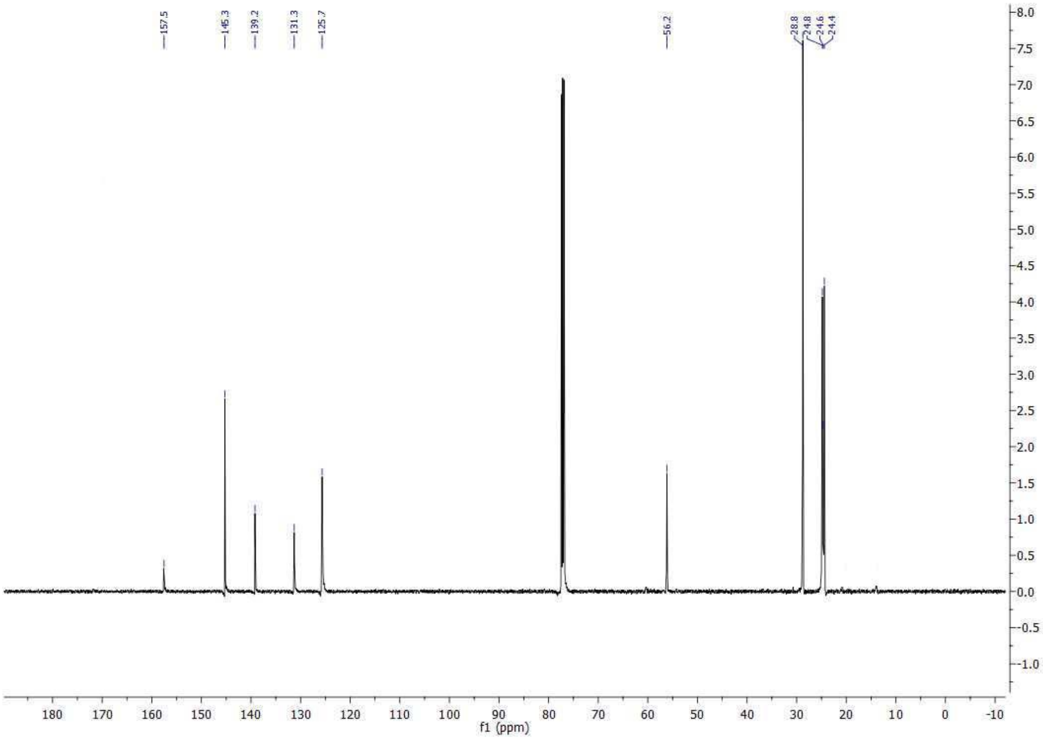

NHC-8.HBr (NHC-8^{H})

^{1}H

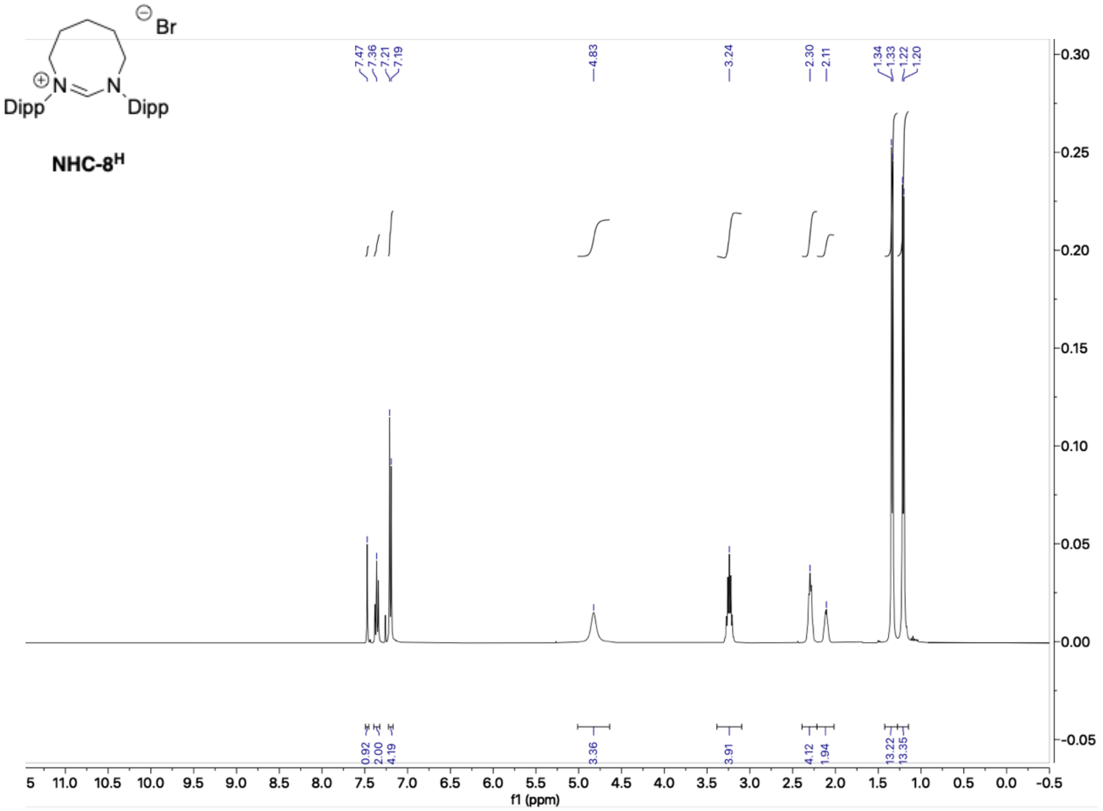

^{13}C{^{1}H}

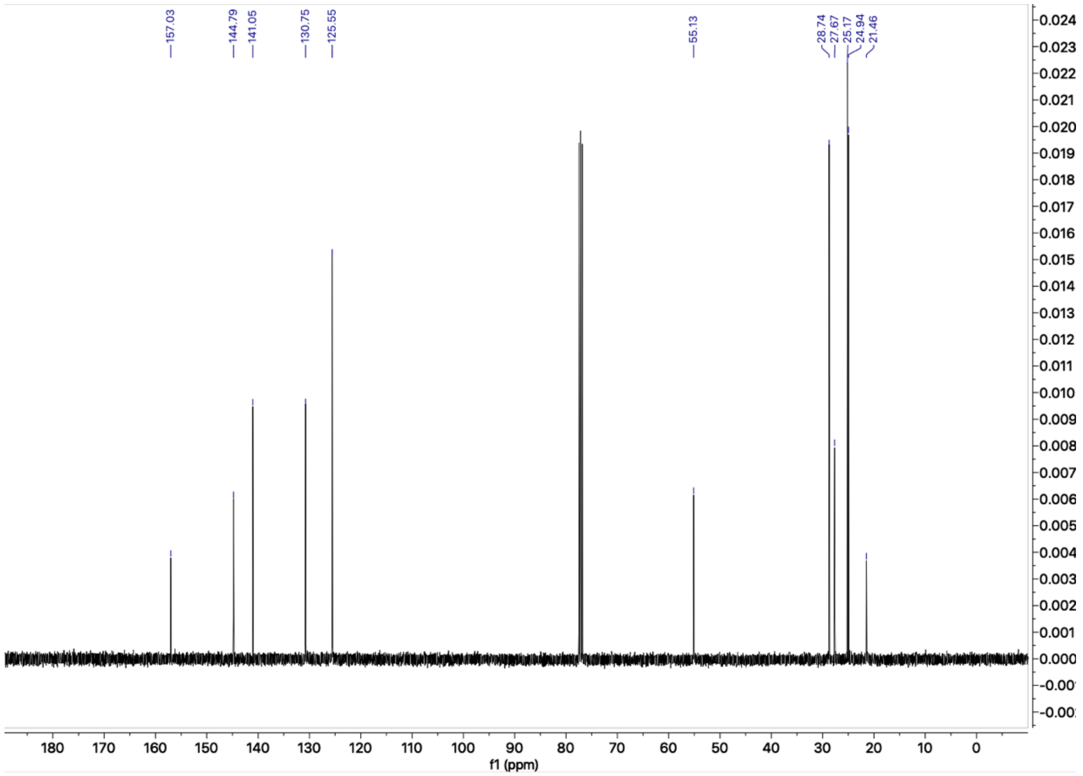

^{Mes}NHC-8.HBr (^{Mes}NHC-8^{H})

^{1}H

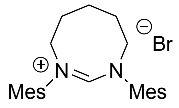

^{Mes}NHC-8^{H}

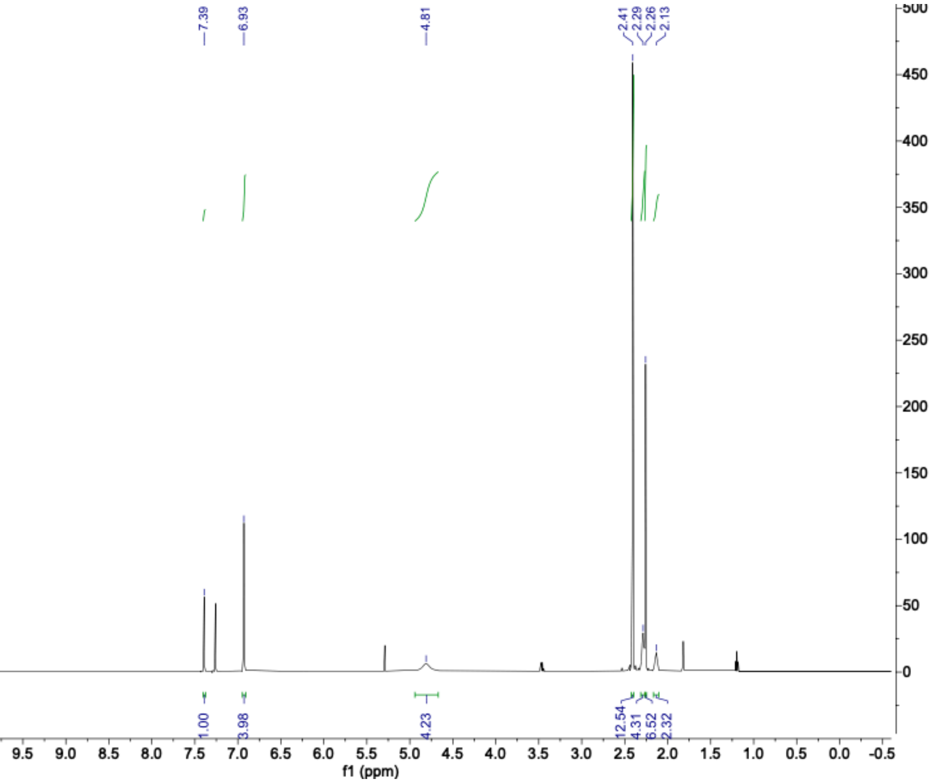

^{13}C{^{1}H}

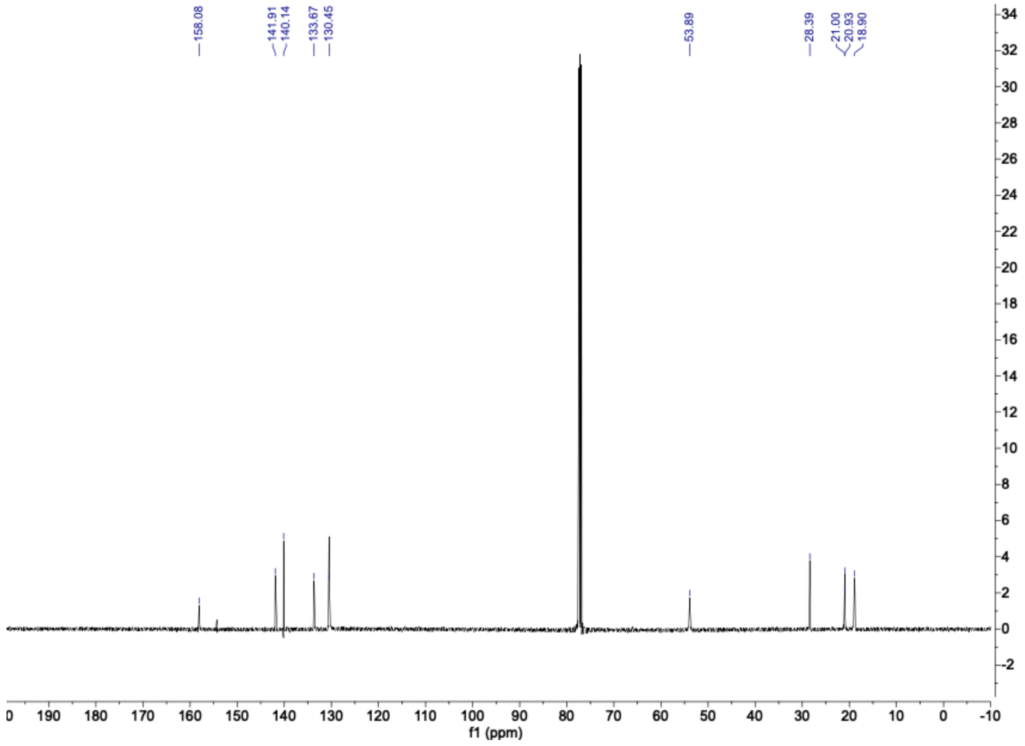

B.3. Free Carbenes

<u>NHC-5:</u> ^{1}H NMR (500 MHz, C_6D_6) δ = 7.28 (t, 2H, J=7.5Hz), 7.19 (d, 4H, J=7.5Hz), 3.34 (s, 4H), 3.27 (sept, 4H, J=6.6Hz), 1.34 (d, 12H, J=6.5Hz), 1.28 (d, 12H, J=6.5Hz) $^{13}C\{^1H\}$ NMR (125 MHz, C_6D_6) δ = 244.6, 147.5, 139.5, 128.3, 124.0, 53.3, 28.4, 24.9, 23.1.

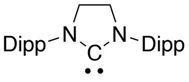

NHC-5

<u>NHC-6:</u> ^{1}H NMR (500 MHz, C_6D_6) δ = 7.25 (dd, 2H, J=7.5Hz), 7.17 (d, 4H, J=7.5Hz), 3.39 (sept, 4H, J=7.0Hz), 2.84 (t, 4H, J=6.0Hz), 1.71 (t, 2H, J=6.0Hz), 1.29 (d, 12H, J=7.0Hz), 1.28 (d, 12H, J=7.0Hz) $^{13}C\{^1H\}$ NMR (125 MHz, C_6D_6) δ = 245.6, 146.2, 145.5, 127.6, 124.1, 43.9, 28.1, 24.6, 23.9, 21.4.

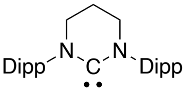

NHC-6

<u>NHC-7:</u> ^{1}H NMR (500 MHz, C_6D_6) δ = 7.23 (dd, 4H, J=7.5Hz), 7.15 (d, 4H, J=7.5Hz), 3.56 (sept, 4H, J=7.0Hz), 3.48 (dd, 4H, J=5.5Hz), 1.80 (dd, 4H, J=5.5Hz), 1.29 (d, 12H, J=7.0Hz), 1.25 (d, 12H, J=7.0Hz) $^{13}C\{^1H\}$ NMR (125 MHz, C_6D_6) δ = 261.0, 148.2, 145.6, 127.1, 124.1, 52.8, 28.2, 26.0, 24.3, 24.0.

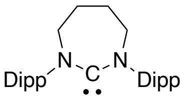

NHC-7

<u>NHC-8:</u> ^{1}H NMR (500 MHz, C_6D_6) δ = 7.23 (t, 2H, J=7.6Hz), 7.14-7.16 (m, 4H), 3.56 (m, 8H), 1.87 (m, 2H), 1.54 (m, 4H), 1.28 (d, 12H, J=7.0Hz), 1.22 (d, 12H, J=7.0Hz), 0.14 (s, HHMDS) $^{13}C\{^1H\}$ NMR (125 MHz, C_6D_6) δ = 255.0, 149.5, 145.0, 126.7, 124.4, 52.1, 29.0, 28.5, 25.2, 24.7, 22.5, 7.21 (HHMDS). *Contains trace hexamethyl-disilazane (HHMDS).*

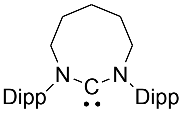

NHC-8

<u>^{Mes}NHC-8:</u> ^{1}H NMR (500 MHz, C_6D_6) δ 6.87 (s, 4H), 3.37 (m, 4H), 2.33 (s, 12H), 2.22 (s, 6H), 1.80 (m, 2H), 1.47 – 1.37 (m, 4H), 0.14 (s, HHMDS) $^{13}C\{^1H\}$ NMR (126 MHz, C_6D_6) δ 250.5, 149.6, 135.2, 134.0, 130.0, 50.8, 29.6, 21.8, 21.0, 19.2, 7.3. *Contains trace hexamethyl-disilazane (HHMDS).*

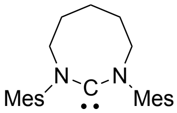

^{Mes}NHC-8

B.4. Free Carbene NMR Spectra

NHC-5

^{1}H

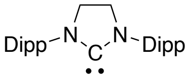

NHC-5

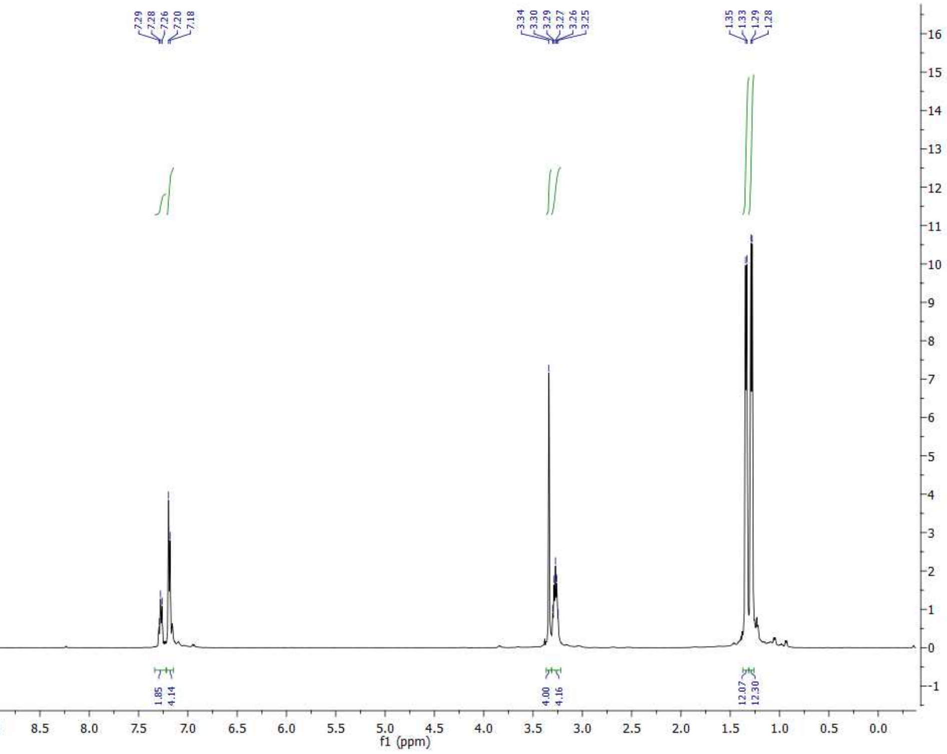

^{13}C{^{1}H}

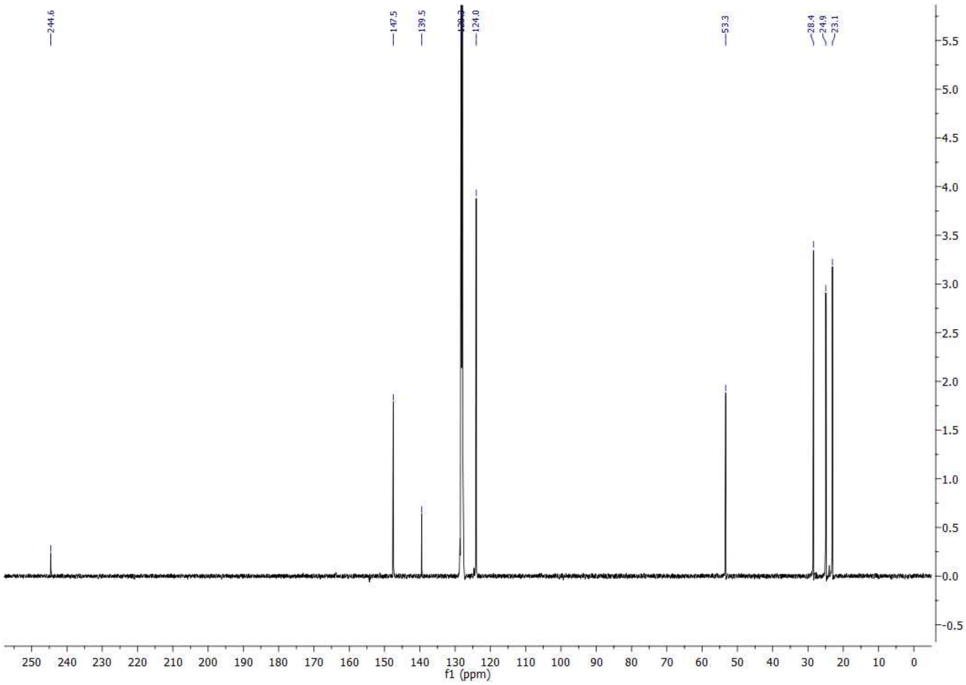

NHC-6

^{1}H

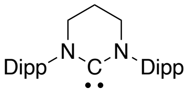

NHC-6

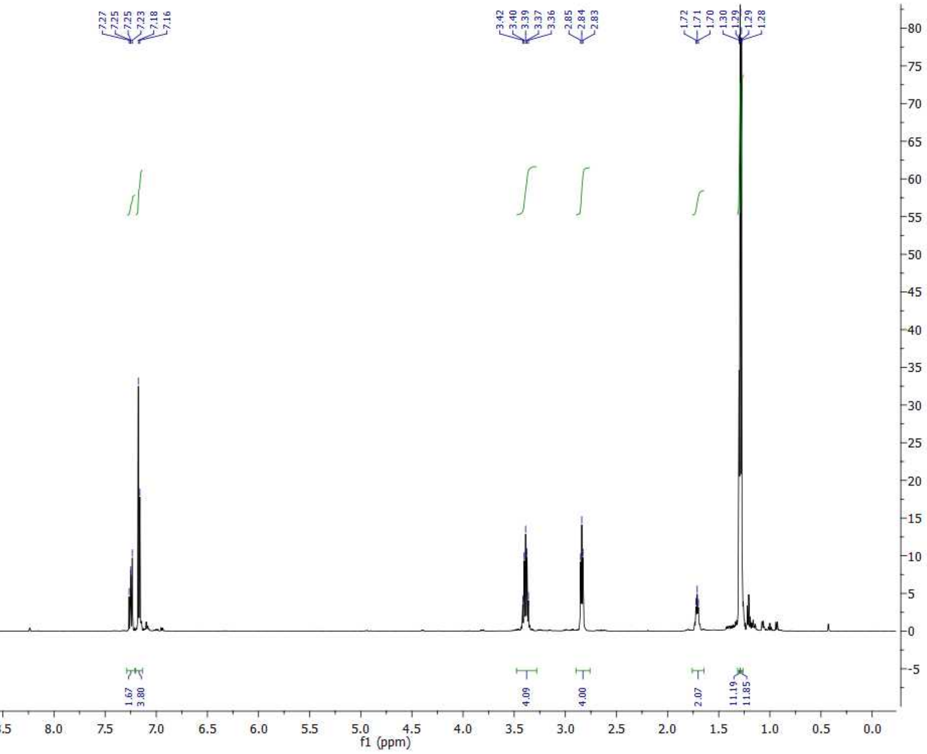

^{13}C{^{1}H}

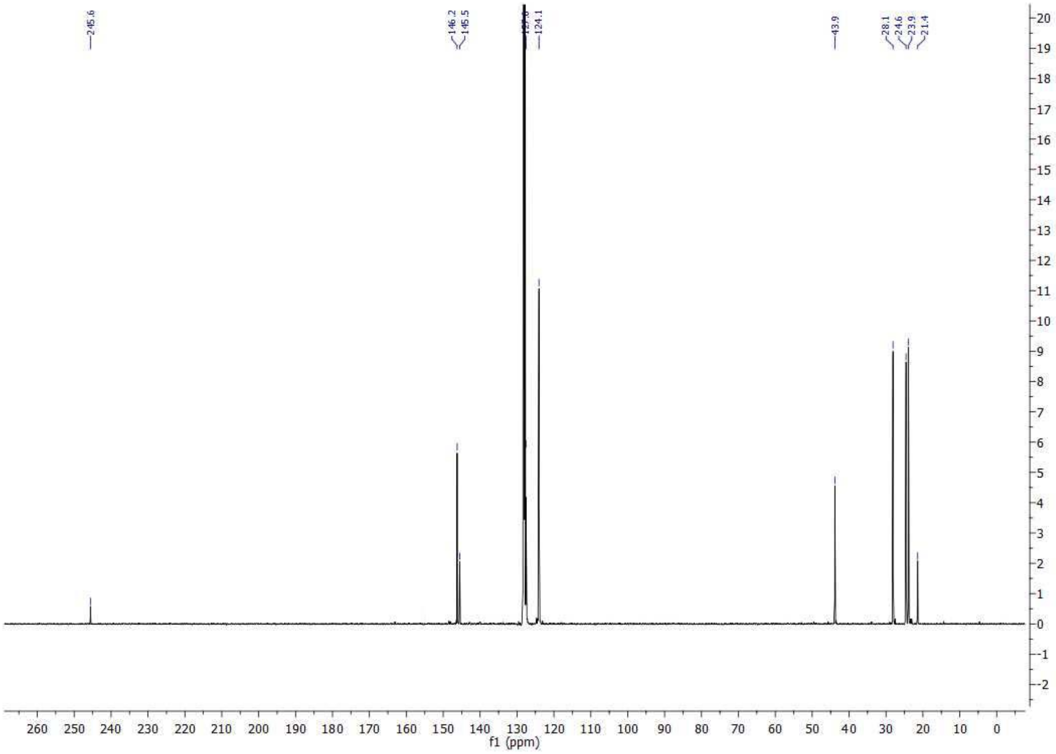

NHC-7

^{1}H

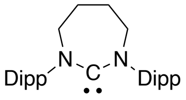

NHC-7

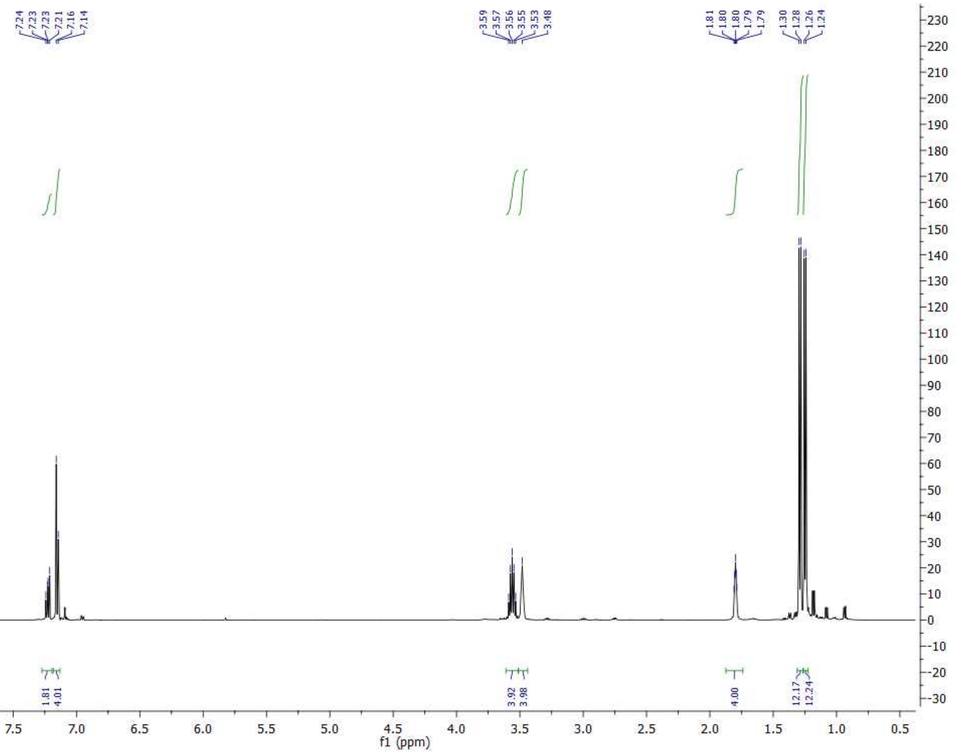

^{13}C{^{1}H}

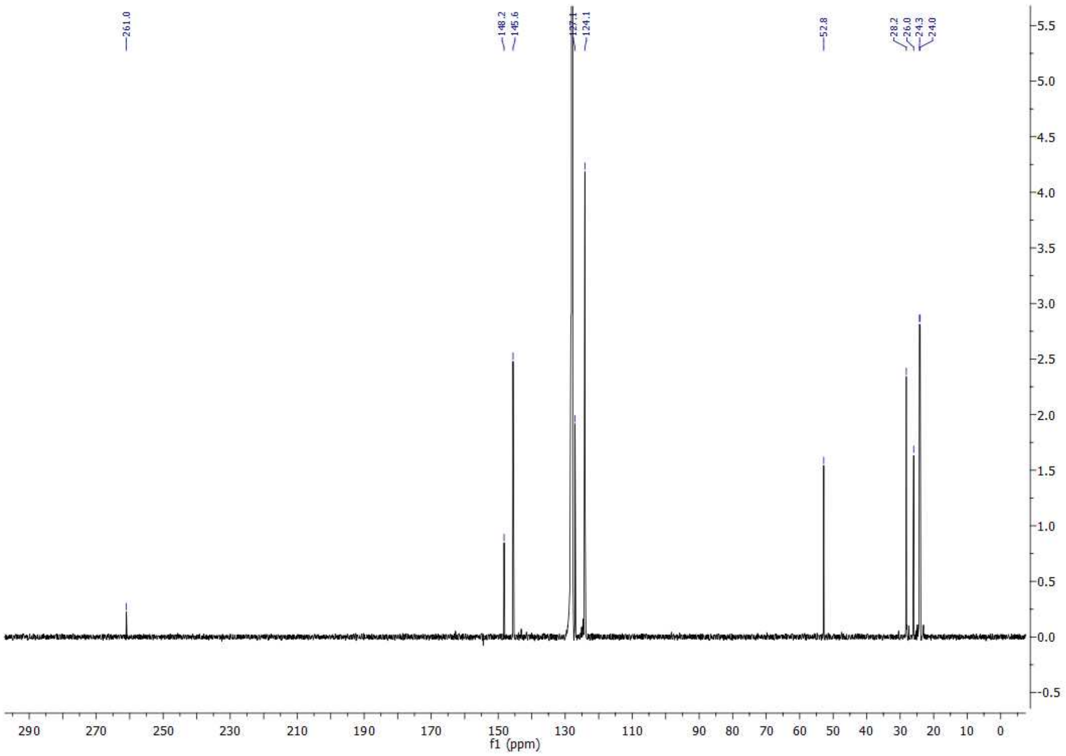

NHC-8

^{1}H

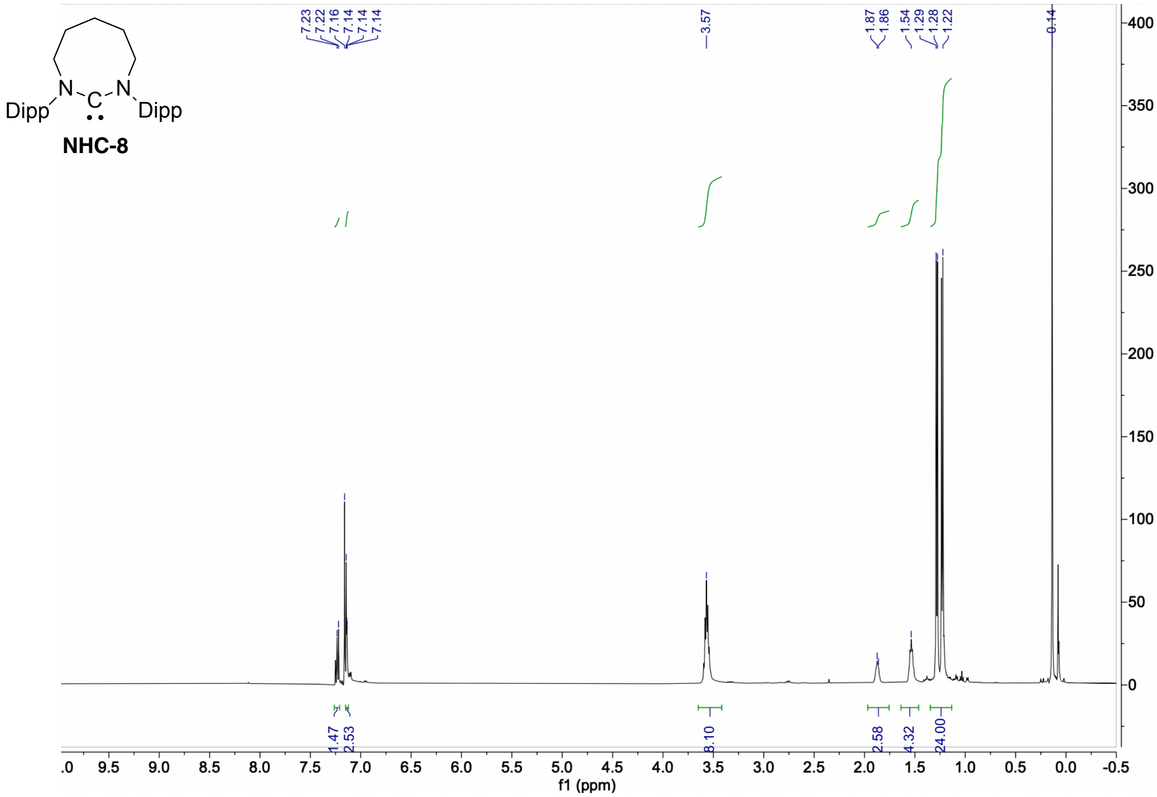

$^{13}C\{^{1}H\}$

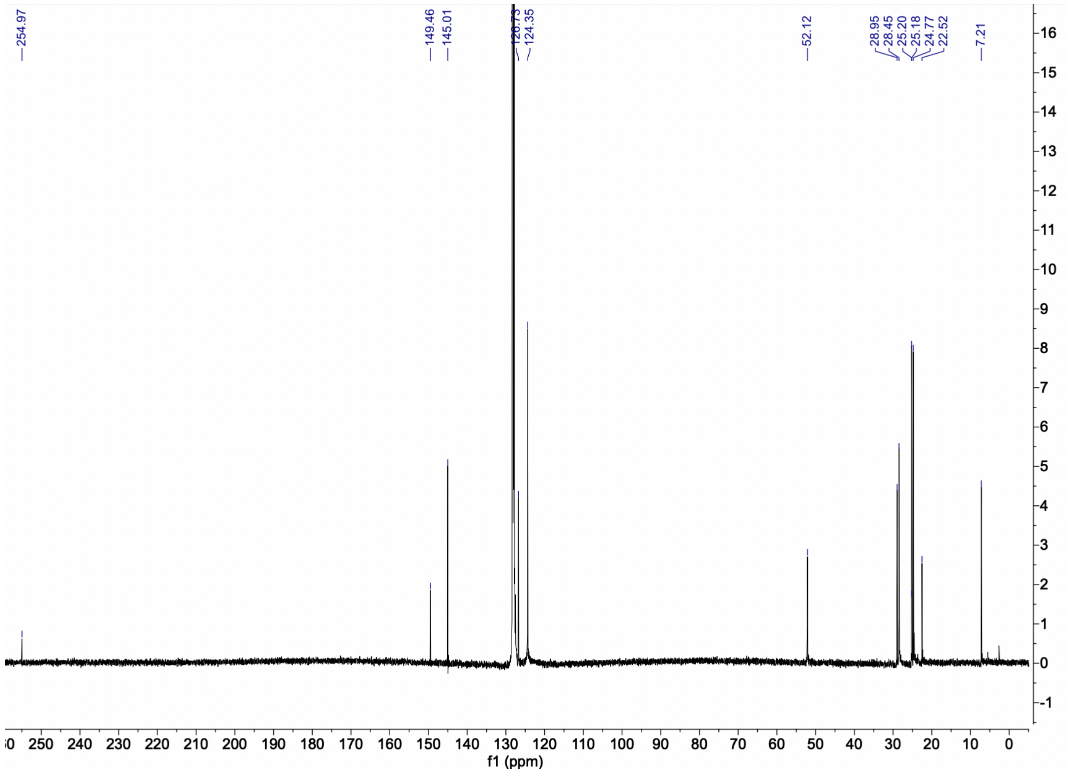

^{Mes}NHC-8

^{1}H

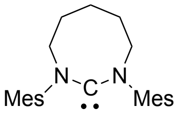

^{Mes}NHC-8

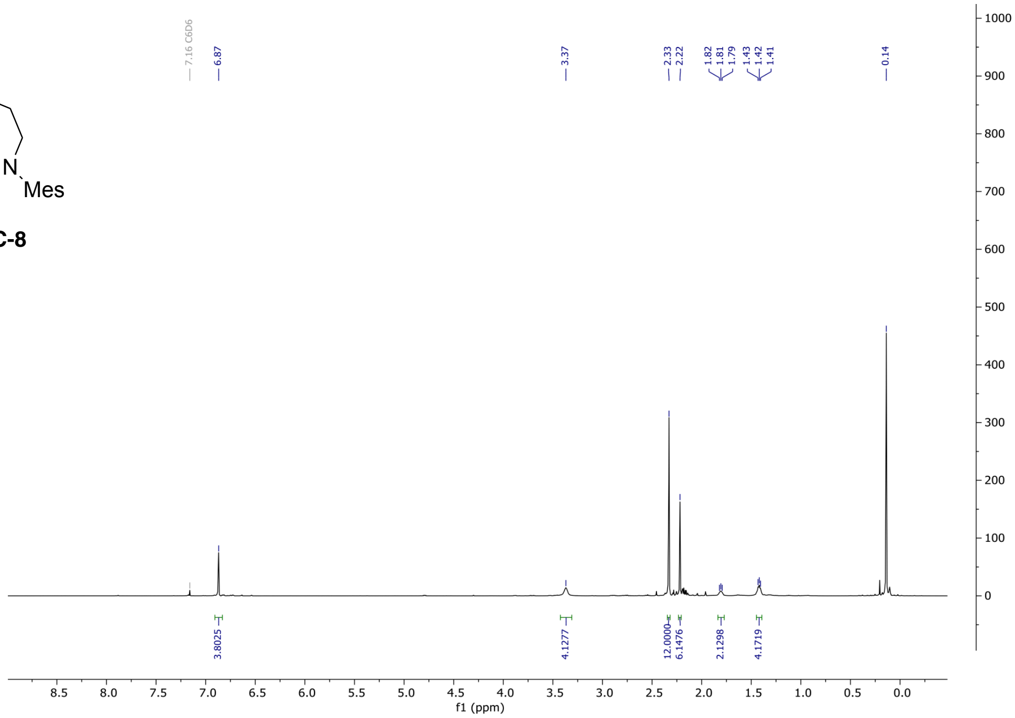

^{13}C{^{1}H}

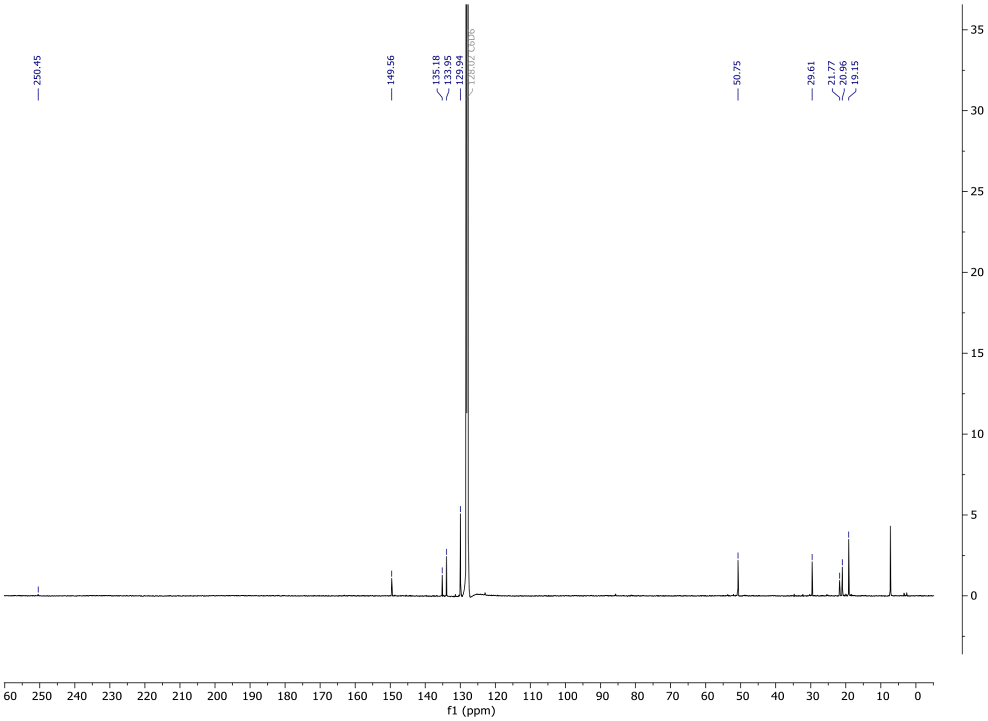

III. Comparison of Reactivity Between NHC-5,6,7,8

A. Reactions Between NHC and Alkynes

A.1. NHC-5 with *p*-Tolylacetylene (1e)

C_6D_6 (0.7 mL) was added under argon to a J.Young NMR tube containing free carbene NHC-5 (39 mg, 1.0 eq) and 4-ethynyltoluene (13 µL, 1.05 eq). The tube was shaken for 5 minutes at room temperature then heated at 80°C. NMR of the resulting colorless solution showed quantitative formation of the carbene(H)(*p*-tolylacetylene) adduct <u>after 4 hours</u>. *Note: At room temperature no reaction is observed after 1h.*

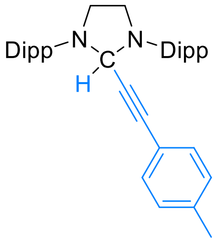

<u>NHC-5(H)(*p*-tolylacetylene) Adduct:</u> ^{1}H NMR (500 MHz, C_6D_6) δ = 7.22-7.29 (m, 4H), 7.16-7.19 (m, 4H), 6.69 (d, 2H, J=8.0Hz), 5.96 (s, 1H), 4.39 (sept, 2H, J=7.0Hz), 3.87 (m, 2H), 3.59 (sept, 2H, J=7.0Hz), 3.56 (m, 2H), 1.90 (s, 3H), 1.43 (d, 6H, J=7.0Hz), 1.33 (d, 6H, J=7.0Hz), 1.32 (d, 6H, J=7.0Hz), 1.32 (d, 6H, J=7.0Hz) ^{13}C{^{1}H} NMR (125 MHz, C_6D_6) δ = 152.0, 149.8, 139.4, 138.0, 132.1, 129.1, 128.0, 124.6, 124.5, 120.7, 89.4, 85.1, 73.0, 52.3, 28.5, 28.4, 25.7,24.7, 24.0, 23.8, 20.6.

^{1}H

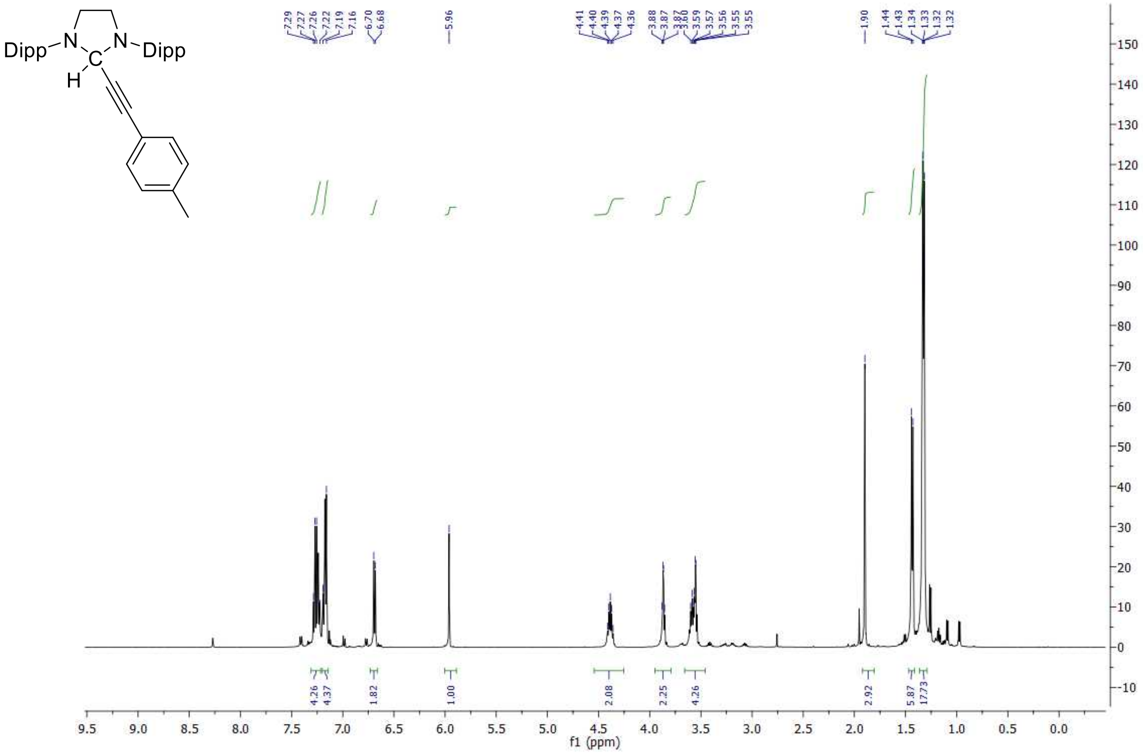

$^{13}C\{^{1}H\}$

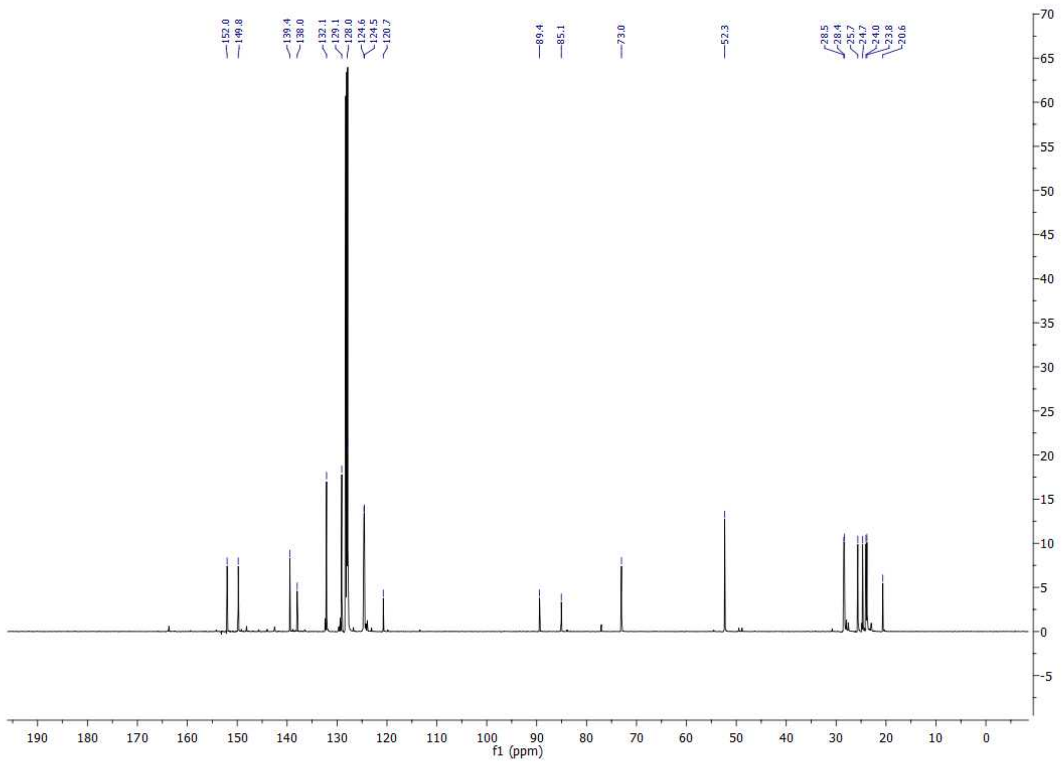

A.2. NHC-6 with *p*-Tolylacetylene (1b)

C_6D_6 (0.7 mL) was added under argon to a J.Young NMR tube containing free carbene NHC-6 (42 mg, 1.0 eq) and 4-ethynyltoluene (15 µL, 1.05 eq). The tube was shaken for 10 minutes at room temperature. NMR of the resulting colorless solution showed quantitative formation of NHC-6(H)(*p*-tolylacetylene) adduct.

Note that NHC-6(H)(p-tolylacetylene) adduct did not afford free NHC-6 when heated at 80°C in vacuo and did not demonstrate exchange upon addition of 1-hexyne.

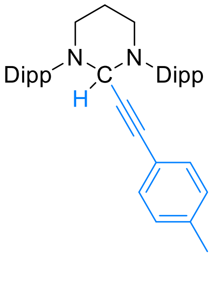

<u>NHC-6(H)(*p*-tolylacetylene) Adduct:</u> ^{1}H NMR (500 MHz, C_6D_6) δ = 7.15-7.19 (m, 4H), 7.03 (dd, 2H, J=6.5Hz), 6.55 (d, 2H, J=8.0Hz), 6.46 (d, 2H, J=8.0Hz), 6.07 (s, 1H), 4.48 (sept, 2H, J=7.0Hz), 3.93 (sept, 2H, J=6.5Hz), 3.38 (m, 4H), 3.13 (m, 2H), 1.69 (s, 3H), 1.36 (d, 6H, J=7.0Hz), 1.36 (d, 6H, J=7.0Hz), 1.29 (d, 6H, J=7.0Hz), 1.24 (d, 6H, J=7.0Hz), 1.23 (m, 2H) $^{13}C\{^{1}H\}$ NMR (125 MHz, C_6D_6) δ = 151.0, 149.5, 144.7, 137.9, 131.3, 128.9, 127.4, 124.4, 123.6, 119.9, 88.3, 86.0, 72.3, 52.2, 30.0, 28.6, 27.6, 25.6, 24.9, 24.8, 23.9, 21.1.

^{1}H

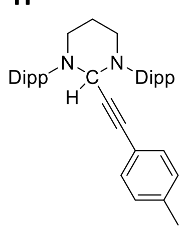

Dipp
N
C
N
Dipp
H

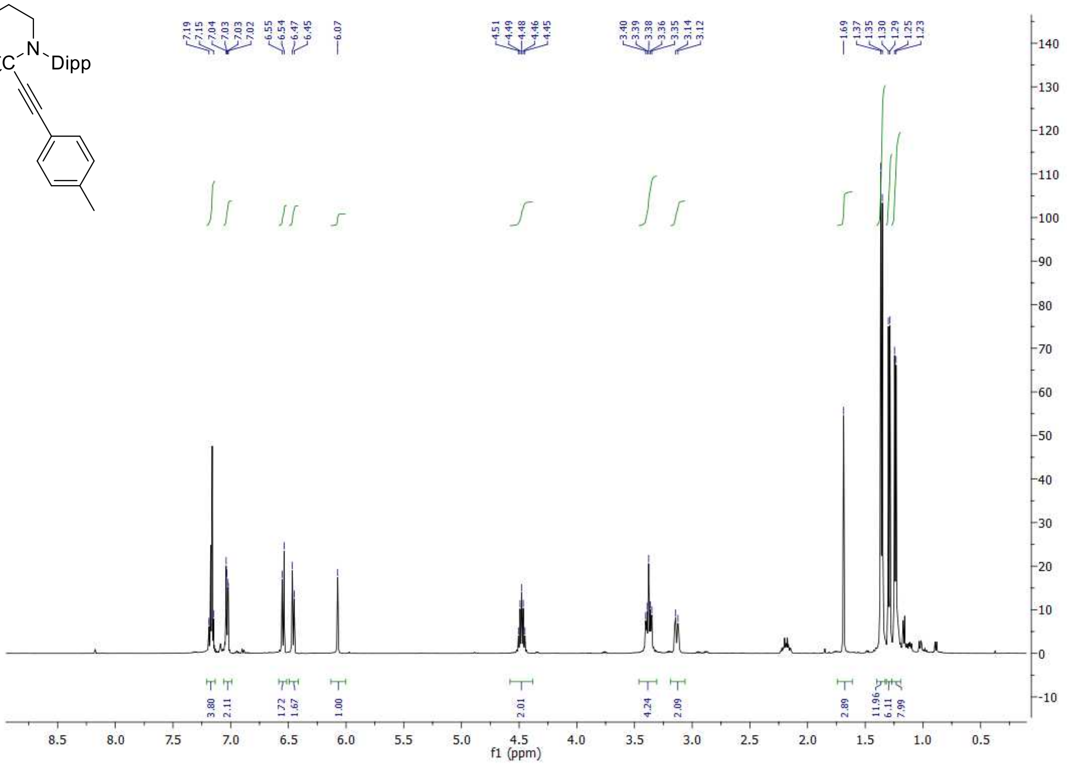

7.19 7.15 7.04 7.03 7.03 7.02 6.55 6.54 6.47 6.45 6.07
4.51 4.49 4.48 4.46 4.45
3.40 3.39 3.38 3.36 3.35 3.14 3.12
1.69 1.37 1.35 1.30 1.29 1.25 1.23
3.80 2.11 1.72 1.67 1.00 2.01 4.24 2.09 2.89 11.96 6.11 7.99
f1 (ppm)

$^{13}C\{^{1}H\}$

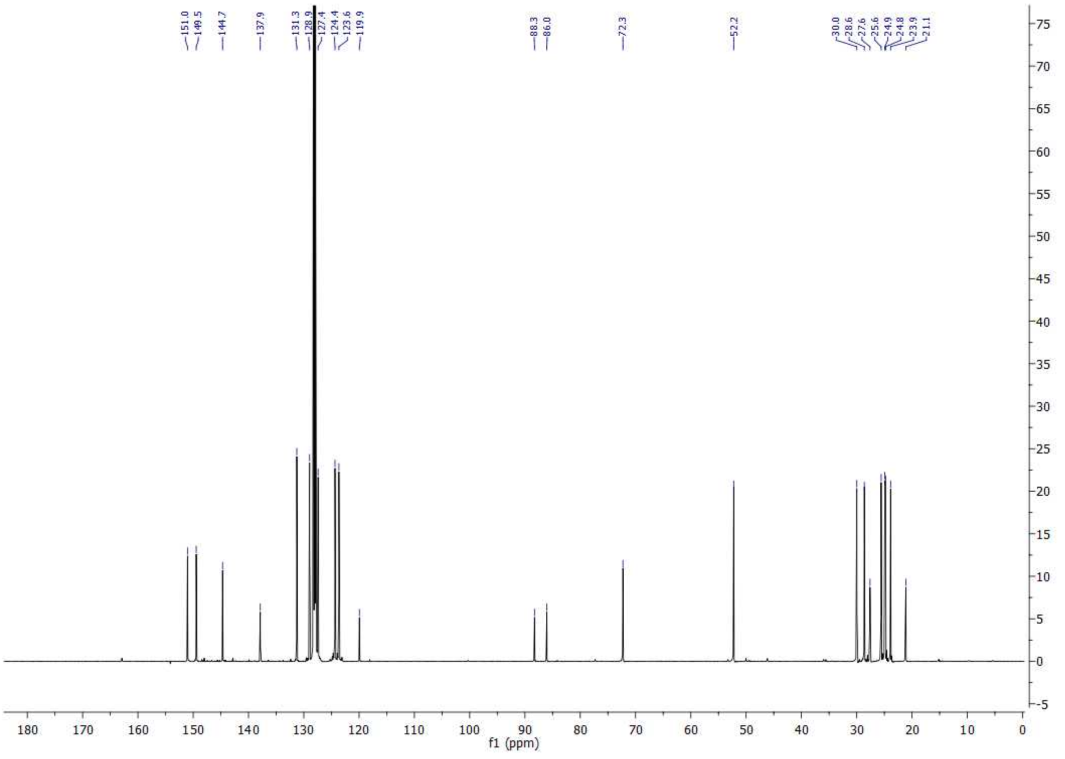

151.0 149.5 144.7 137.9 131.3 128.9 127.4 124.4 123.6 119.9
88.3 86.0 72.3 52.2
30.0 28.6 27.6 25.6 24.9 24.8 23.9 21.1
f1 (ppm)

A.3. NHC-7 with *p*-Tolylacetylene (1c)

C_6D_6 (0.7 mL) was added under argon to a J.Young NMR tube containing free carbene NHC-7 (47 mg, 1.0 eq) and 4-ethynyltoluene (14.1 µL, 1.05 eq). The tube was shaken for 10 minutes at room temperature. NMR of the resulting colorless solution showed quantitative formation of NHC-7(H)(*p*-tolylacetylene) adduct. Slow evaporation of C_6D_6 under argon gave colorless crystals. Note that the complex ^1H NMR signals of the aliphatic region of the spectrum can be explained by the presence of conformers. This is supported by the ^{13}C NMR spectrum which displays only a single species in solution.

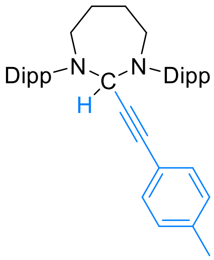

<u>NHC-7(H)(*p*-tolylacetylene) Adduct:</u> ^1H NMR (500 MHz, C_6D_6) δ = 7.29-7.33 (m, 6H), 7.06 (d, 2H, J=7.0Hz), 6.79 (d, 2H, J=7.0Hz), 5.87 (s, 1H), 4.30 (sept, 2H, J=6.5Hz), 3.93 (sept, 2H, J=6.5Hz), 3.92 (m, 4H), 1.99 (s, 3H), 1.95-2.05 (m, 2H), 1.72-1.78 (m, 2H), 1.53 (d, 6H, J=6.5Hz), 1.45 (d, 6H, J=6.5Hz), 1.45 (d, 6H, J=6.5Hz), 1.41 (d, 6H, J=6.5Hz)
$^{13}C\{^1H\}$ NMR (125 MHz, C_6D_6) δ = 150.1, 148.8, 145.3, 137.7, 131.9, 129.0, 127.2, 124.9, 124.6, 120.9, 89.6, 86.9, 73.9, 52.9, 30.4, 28.6, 28.3, 25.4, 24.7, 24.7, 24.1, 20.6
HRMS (ESI): m/z calculated for $C_{38}H_{51}N_2$ $[M+H]^+$ 535.4047, found 535.4042.

^{1}H

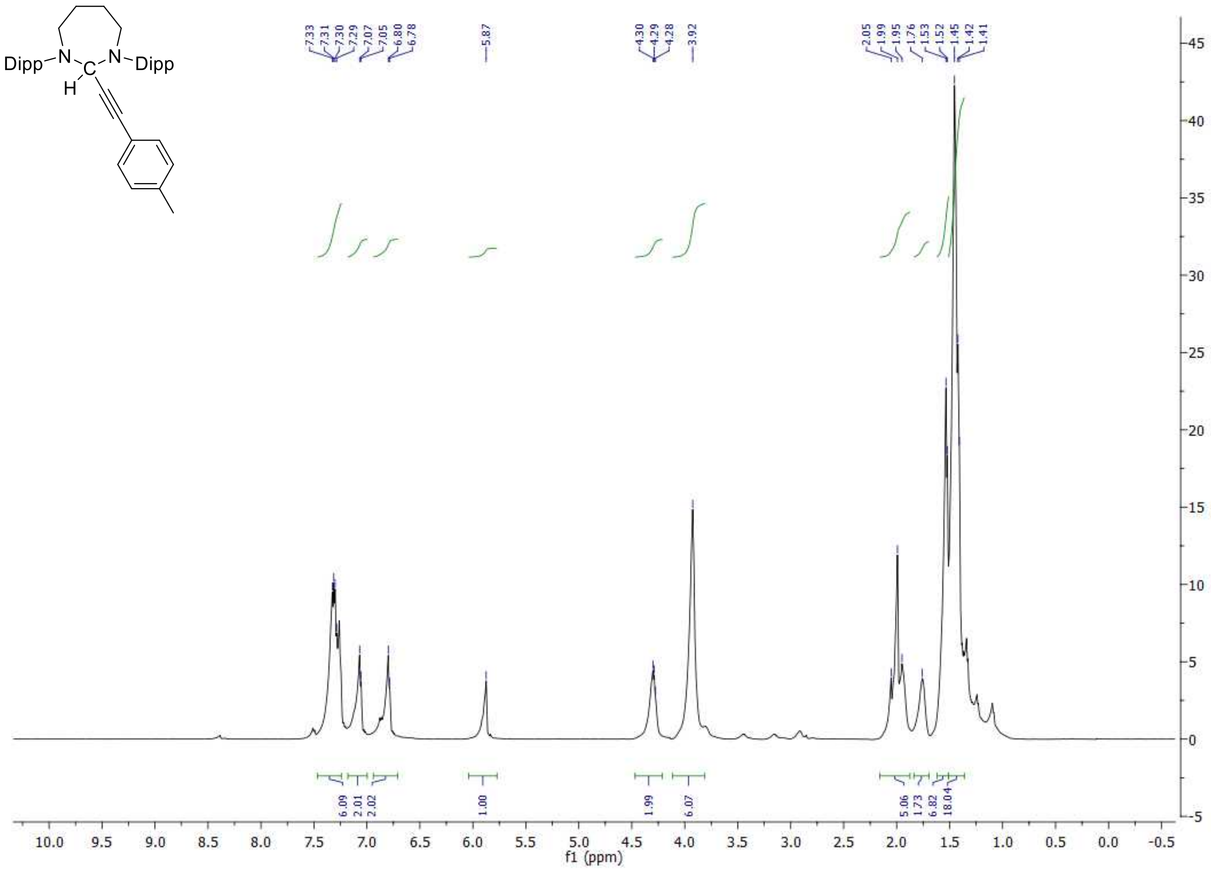

$^{13}C\{^{1}H\}$

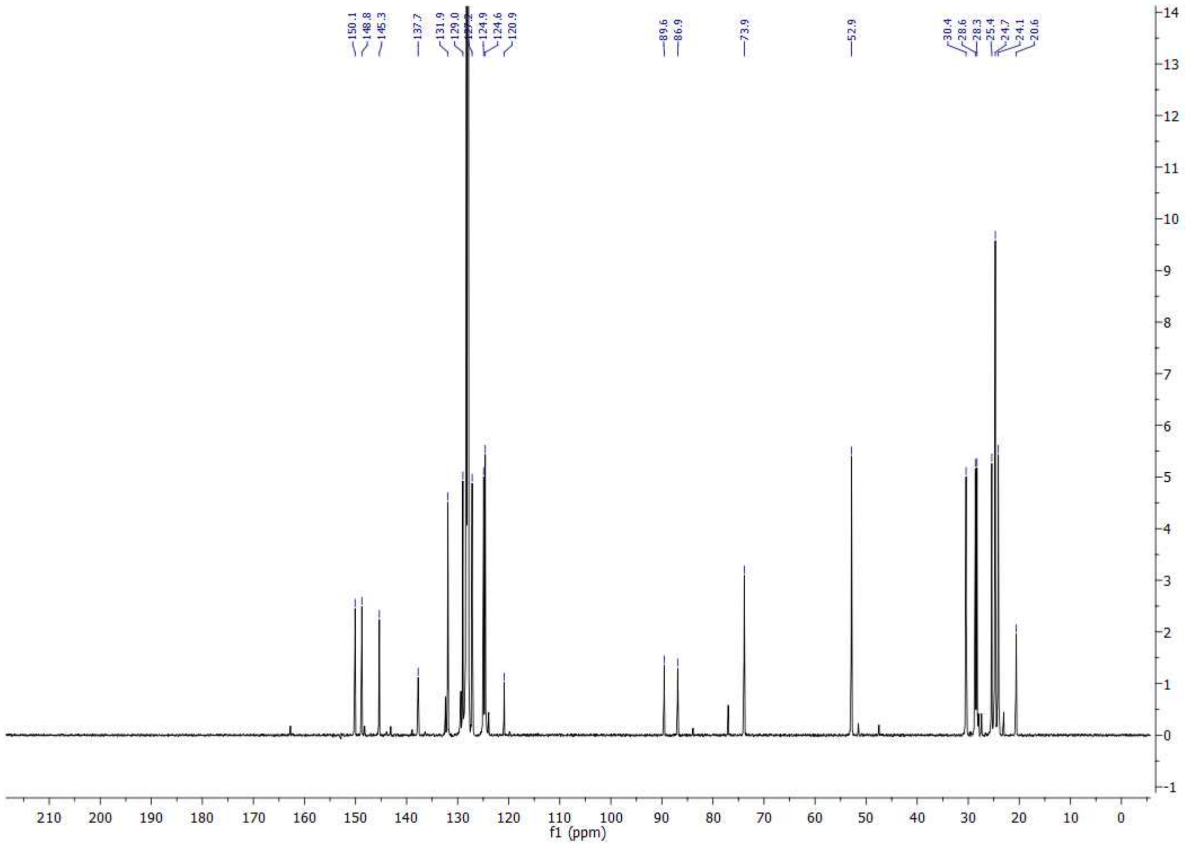

A.4. NHC-8 with *p*-Tolylacetylene (1d) conformers

C_6D_6 (0.7 mL) was added under argon to a J.Young NMR tube containing free carbene NHC-8 (43 mg, 1.0 eq) and 4-ethynyltoluene (13 µL, 1.05 eq). The tube was shaken for 10 minutes at room temperature. NMR of the resulting colorless solution showed quantitative formation of NHC-8(H)(*p*-tolylacetylene) adduct. The NMR contains residual HHMDS that was present with the free NHC-8 carbene. Note that the complex ^{1}H NMR signals of the aliphatic region of the spectrum can be explained by the presence of conformers. This is supported by the ^{13}C NMR spectrum which only display a single species in solution.

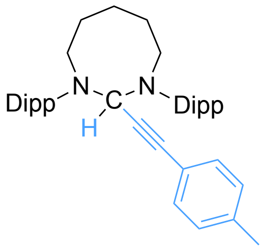

<u>NHC-8(H)(*p*-tolylacetylene) Adduct:</u> ^{1}H NMR (400 MHz, C_6D_6) δ = 7.11-7.16 (m, 6H), 7.00 (d, 2H, J=7.9Hz), 6.73 (d, 2H, J=7.4Hz), 5.63 (s, 1H), 4.33 (m, 2H, J=6.5Hz), 3.81 (m, 2H), 3.66 (m, 4H), 1.93 (s, 3H), 1.90-1.94 (m, 4H), 1.64 (m, 2H), 1.36 (d, 12H, J=6.9Hz), 1.31 (d, 12Hz, J=6.9Hz), $^{13}C\{^{1}H\}$ NMR (100 MHz, C_6D_6) δ = 149.7, 148.5, 147.4, 137.9, 131.8, 129.1, 127.0, 124.9, 89.1, 86.2, 77.1, 53.1, 30.7, 28.9, 26.1, 25.2, 25.0, 24.4.

^{1}H

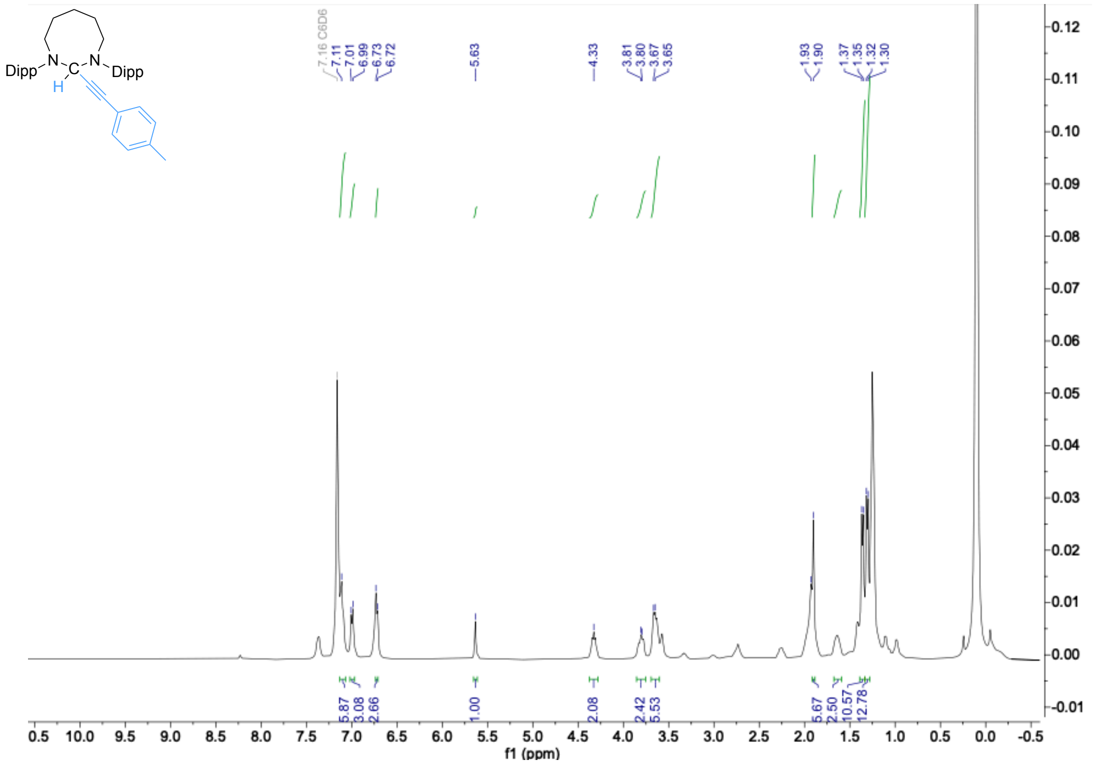
Dipp
N
C
N
Dipp
H
7.16 C6D6
7.11
7.01
6.99
6.73
6.72
5.63
4.33
3.81
3.80
3.67
3.65
1.93
1.90
1.37
1.35
1.32
1.30
5.87
3.08
2.66
1.00
2.08
2.42
5.53
5.67
2.50
10.57
12.78
f1 (ppm)

^{13}C{^{1}H}

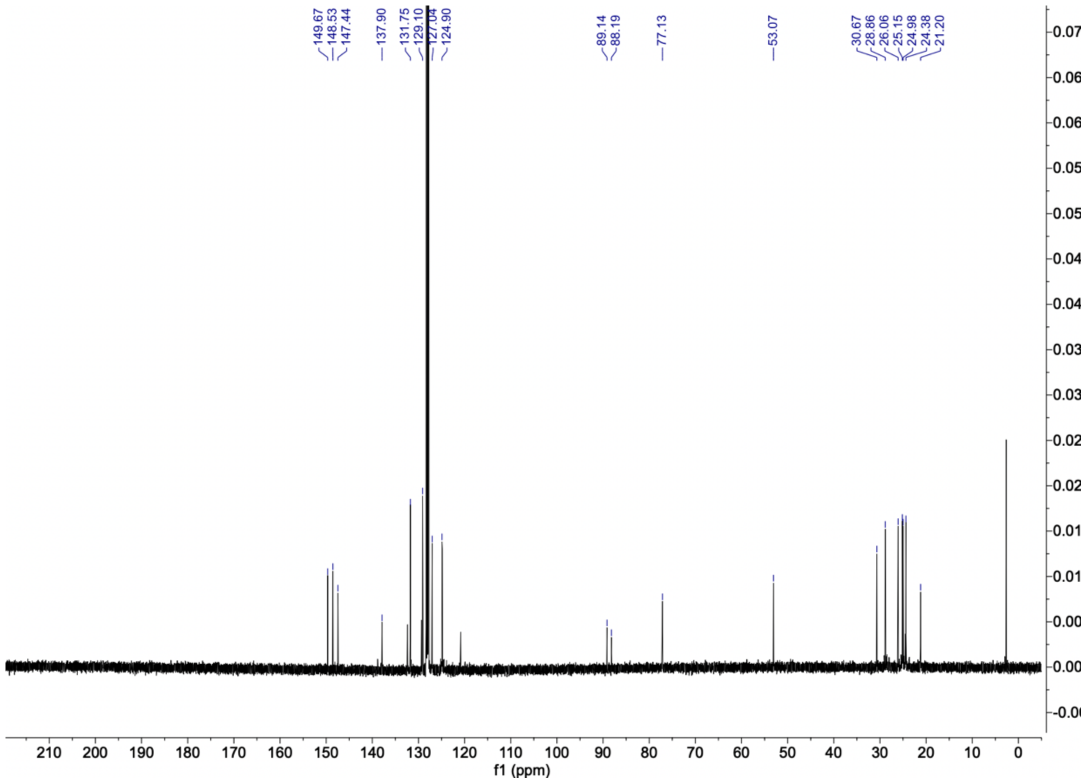
149.67
148.53
147.44
137.90
131.75
129.10
127.04
124.90
89.14
88.19
77.13
53.07
30.67
28.86
26.06
25.15
24.98
24.38
21.20
f1 (ppm)

B. Reactions Between NHC and Isocyanides

No reaction was observed after 12h by reacting free NHC-5 or NHC-6 with one equivalent of adamantyl isocyanide at room temperature in benzene.

B.1. NHC-7 with Adamantyl Isocyanide (2b)

C_6D_6 (0.7 mL) was added under argon to a J.Young NMR tube containing free carbene NHC-7 (37.1 mg, 1.0 eq) and adamantyl isocyanide (10.9 mg, 1.05 eq). The tube was shaken for 10 minutes at room temperature. NMR of the resulting colorless solution showed quantitative formation of the corresponding ketenimine. Slow evaporation of C_6D_6 under argon gave colorless crystals.

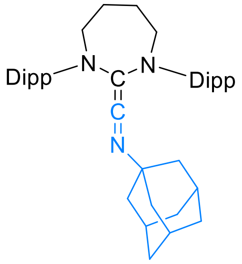

NHC-7 Adamantyl ketenimine: ^{1}H NMR (500 MHz, C_6D_6) δ = 7.20 (t, 1H, J=7.0Hz), 7.17 (dd, 4H, J=7.0Hz), 7.11 (t, 1H, J=7.0Hz), 3.83 (m, 4H), 3.73 (sept, 2H, J=7.0Hz), 3.63 (sept, 2H, J=7.0Hz), 1.86 (m, 4H), 1.61 (m, 2H), 1.58 (d, 6H, J=7.0Hz), 1.52 (m, 2H), 1.43 (t, 8H, J=7.0Hz), 1.38 (m, 1H), 1.33 (d, 6H, J=7.0Hz), 1.32 (d, 6H, J=7.0Hz), 1.21 (d, 6H, J=7.0Hz) ^{13}C{^{1}H} NMR (125 MHz, C_6D_6) δ = 216.9, 148.7, 146.1, 143.6, 126.9, 124.6, 124.2, 124.1, 59.3, 55.1, 42.4, 36.2, 30.6, 29.5, 28.4, 28.2, 25.3, 25.1, 24.0, 23.4 HRMS (ESI): m/z calculated for $C_{40}H_{58}N_3$ $[M+H]^+$ 580.4625, found 580.4620.

^{1}H

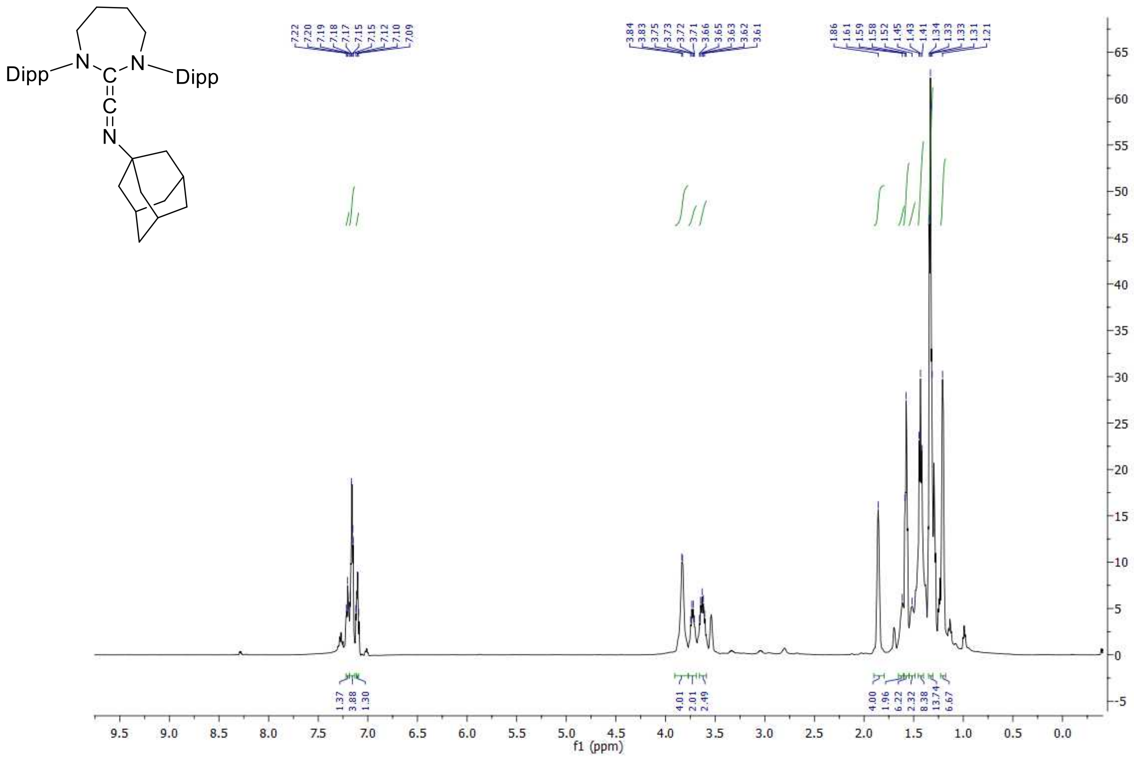

$^{13}C\{^1H\}$

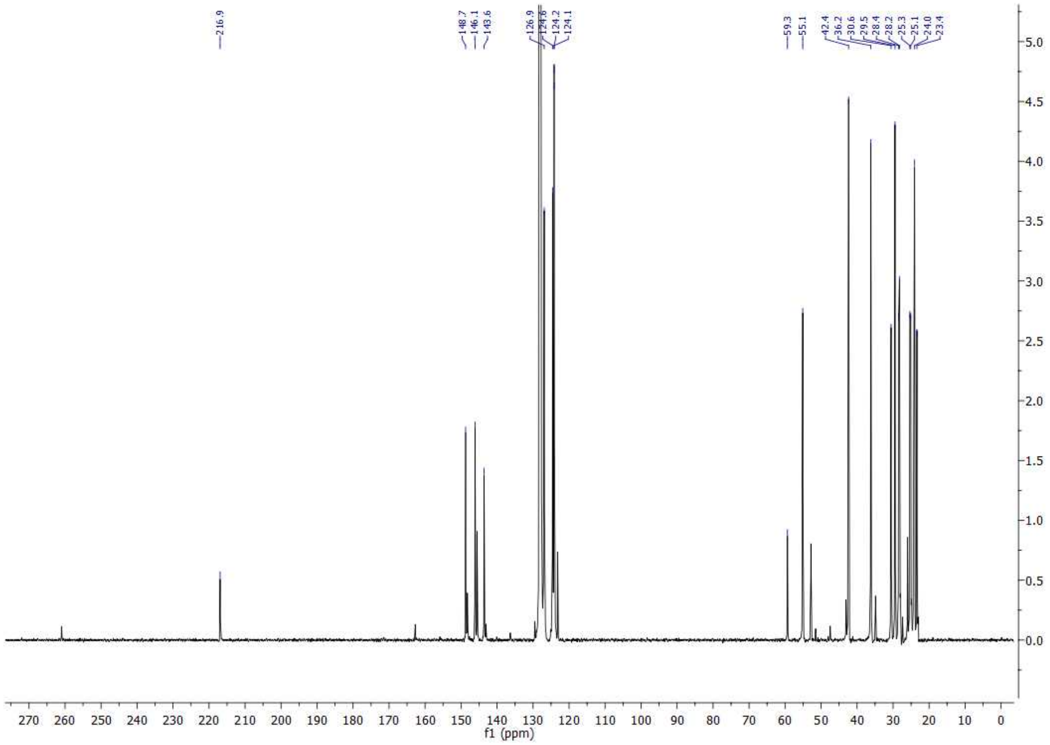

B.2. NHC-8 with Adamantyl Isocyanide (2c)

C_6D_6 (0.7 mL) was added under argon to a J.Young NMR tube containing free carbene NHC-8 (76.2 mg, 1.0 eq) and adamantyl isocyanide (31.2 mg, 1.05 eq). The tube was shaken for 10 minutes and then left to stand for 1 hour at room temperature. NMR of the resulting yellow solution showed quantitative formation of the corresponding ketenimine.

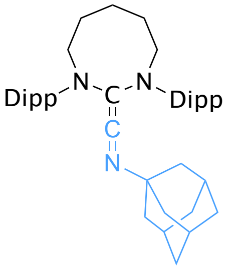

<u>NHC-8 2,6-Dimethylphenyl ketenimine:</u> ^1H NMR (500 MHz, C_6D_6) δ = 7.34 (t, 2H, J=7.5Hz), 7.23 (d, 4H, J=7.5Hz), 4.08 (m, 4H), 3.84 (s, 1H, J=6.6Hz), 3.64-3.68 (m, 5H), 1.73 (m, 4H), 1.44-1.47 (m, 4H), 1.38 (d, 12H, J=6.6Hz), 1.33(d, 12H, J=6.6Hz), 1.26 (m, 6H) $^{13}C\{^1H\}$ NMR (125 MHz, C_6D_6) δ = 211.5149.5, 145.0, 126.7, 124.3, 5939, 52.1, 34.5, 35.3, 29.9, 28.9, 28.5, 25.2, 24.8.

^1H

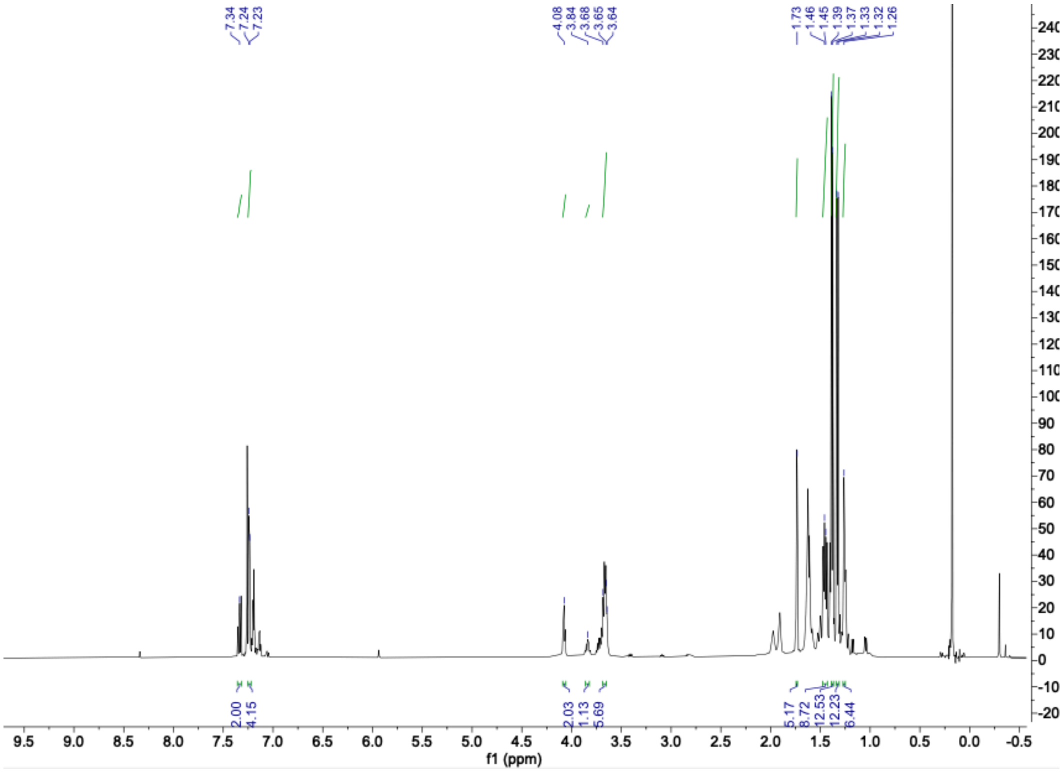
7.34
7.24
7.23
4.08
3.84
3.68
3.65
3.64
1.73
1.46
1.45
1.39
1.37
1.33
1.32
1.26
2.00
4.15
2.03
1.13
5.69
5.17
8.72
12.53
12.23
6.44
f1 (ppm)

$^{13}C\{^{1}H\}$

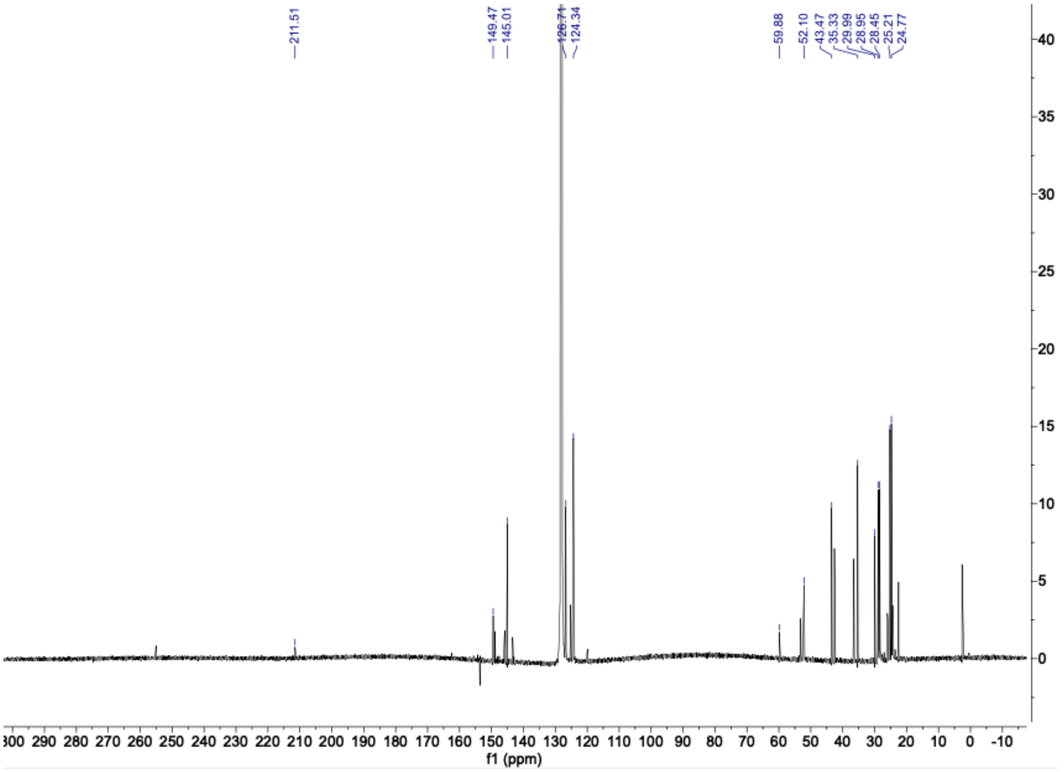
211.51
149.47
145.01
126.71
124.34
59.88
52.10
43.47
35.33
29.99
28.95
28.45
25.21
24.77
f1 (ppm)

C. Reactions Between NHC and Ammonia

No reaction was observed with free NHC-5, NHC-6, or NHC-8 in the presence of ammonia.

C.1. NHC-7 with Ammonia (3a)

In a dry pressure Schlenk under argon, free NHC-7 (35 mg) was dissolved in anhydrous THF. The solution was degassed 3 times. NH_3 (1 bar) was added at -47°C. Then the solution was stirred overnight from -47°C to room temperature. The solvent was removed under vacuum and NHC-7(H)(NH_2) adduct was obtained as a yellowish solid – quantitative reaction. Slow diffusion of pentane into a saturated solution of NHC-7(H)(NH_2) adduct in C_6D_6 under argon gave colorless crystals.

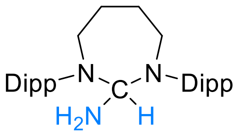

NHC-7(H)(NH_2) Adduct: ^1H NMR (500 MHz, C_6D_6) δ = 7.18 (t, 2H, J=7.5Hz), 7.10-7.12 (m, 4H), 5.25 (t, 1H, J=6.0Hz), 3.99 (sept, 2H, J=6.5Hz), 3.78 (sept, 2H, J=6.5Hz), 3.64 (m, 4H), 1.69 (m, 2H), 1.57 (m, 2H) 1.27 (d, 6H, J=7.5Hz), 1.26 (d, 6H, J=7.5Hz), 1.24 (d, 6H, J=7.5Hz), 1.23 (d, 6H, J=7.5Hz) $^{13}C\{^1H\}$ NMR (125 MHz, C_6D_6) δ = 149.9 (C), 148.5 (C), 145.3 (C), 127.0 (CH), 125.0 (CH), 124.8 (CH), 85.8 (CH), 51.6 (CH_2), 30.6 (CH_2), 28.3 (CH), 28.0 (CH), 25.0 (CH_3), 24.6 (CH_3), 24.1 (CH_3), 24.0 (CH_3) HRMS (ESI): m/z calculated for C29H43N2 [M-NH3+H]+ 419.3421, found 419.3416.

^1H

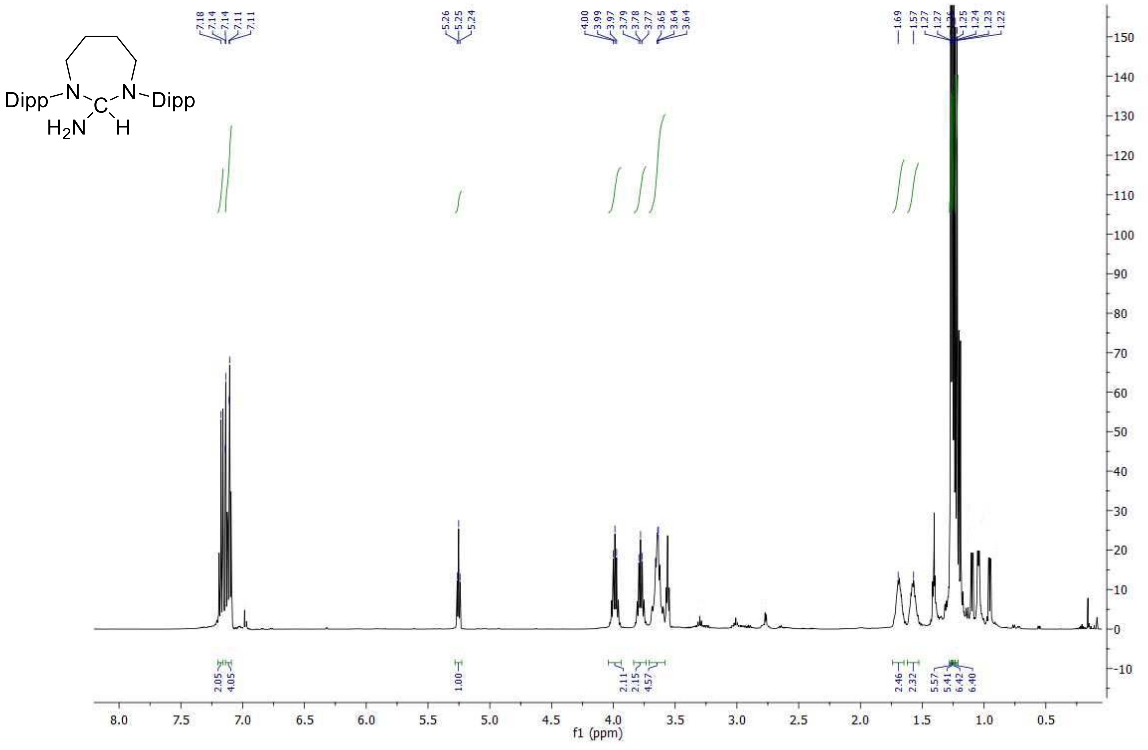

$^{13}C\{^1H\}$

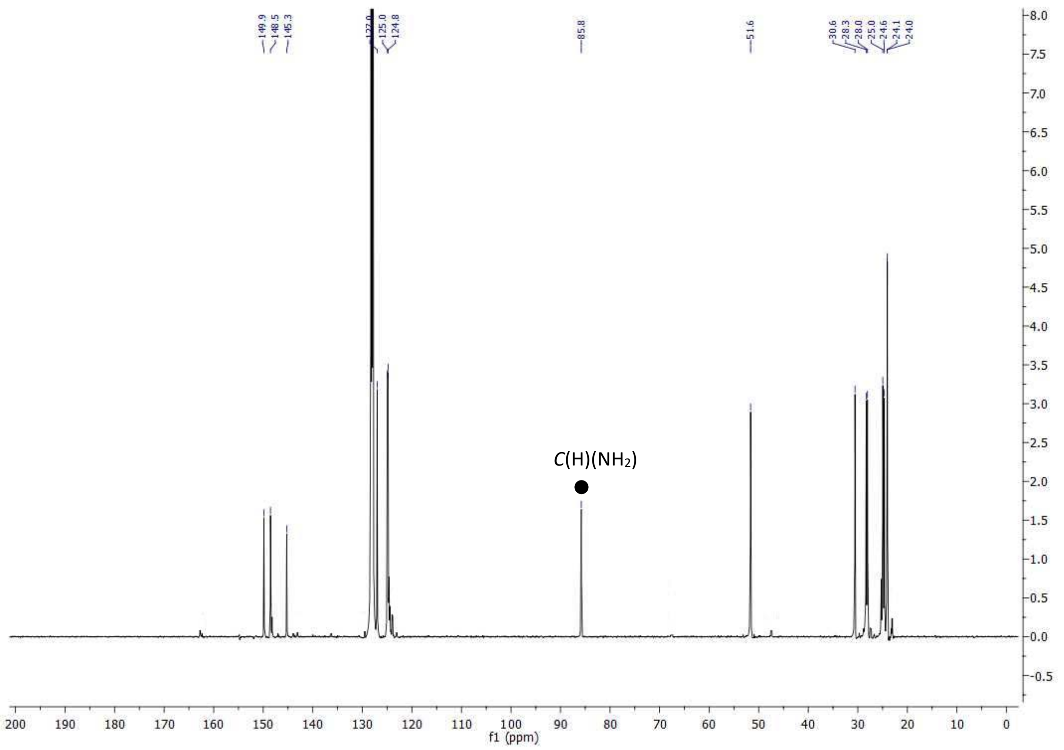

C.2. MesNHC-8 with Ammonia (3c)

In a dry J Young NMR tube in an Ar-filled glovebox, free MesNHC-8 (35 mg) was dissolved in C_6D_6. The NMR tube was brought out of the glovebox and the solution was degassed 3 times (Freeze/Pump/Thaw). NH_3 (~2 bar) was added at -78°C. Then the solution was slowly warmed to room temperature and carefully inverted multiple times to mix. Compound **3c** was too reactive to be isolated and was characterized in situ.

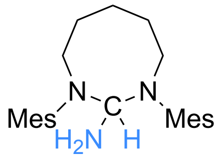

<u>MesNHC-8(H)(NH_2) Adduct:</u> ^{1}H NMR (500 MHz, C_6D_6) δ = 6.86 (s, 4H), 5.16 (t, 1H, J=5.5Hz), 3.47 (m, 4H), 2.20 (s, 6H), 2.18 (s, 6H), 2.15 (s, 6H), 1.62 (m, 2H), 1.48 (m, 4H). $^{13}C\{^1H\}$ NMR (125 MHz, C_6D_6) δ = 149.7, 134.3, 130.7, 129.8, 85.7, 50.6, 29.8, 21.9, 20.9, 19.3.

^{1}H

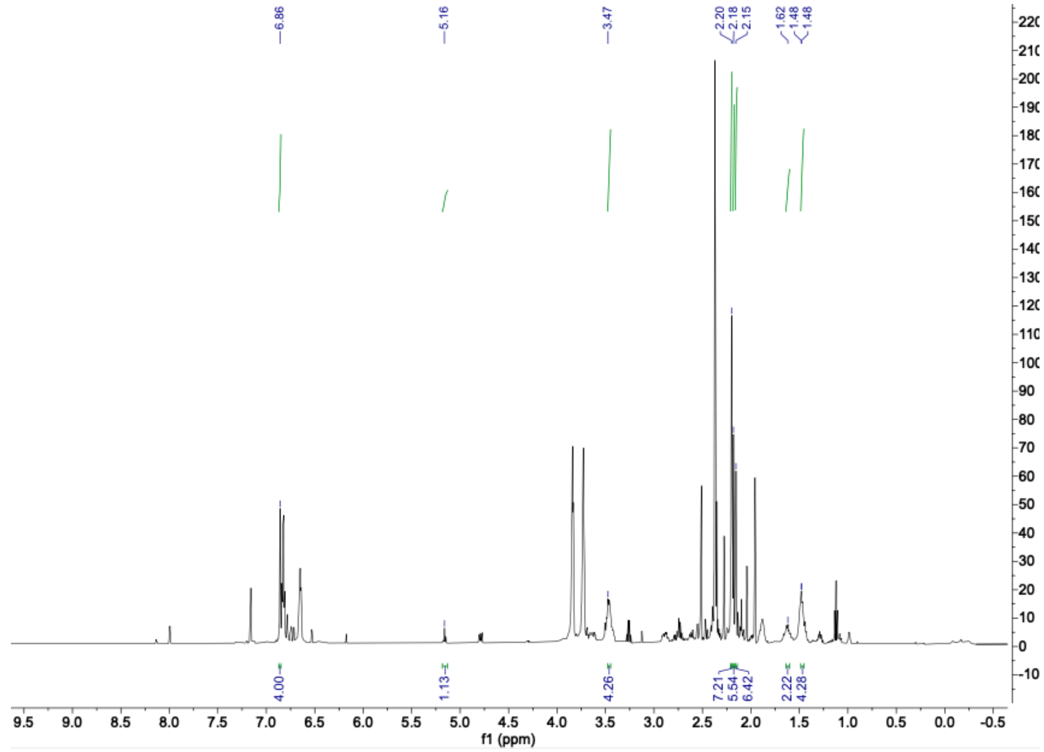

6.86
5.16
3.47
2.20
2.18
2.15
1.62
1.48
1.48
4.00
1.13
4.26
7.21
5.54
6.42
2.22
4.28
f1 (ppm)

$^{13}C\{^{1}H\}$

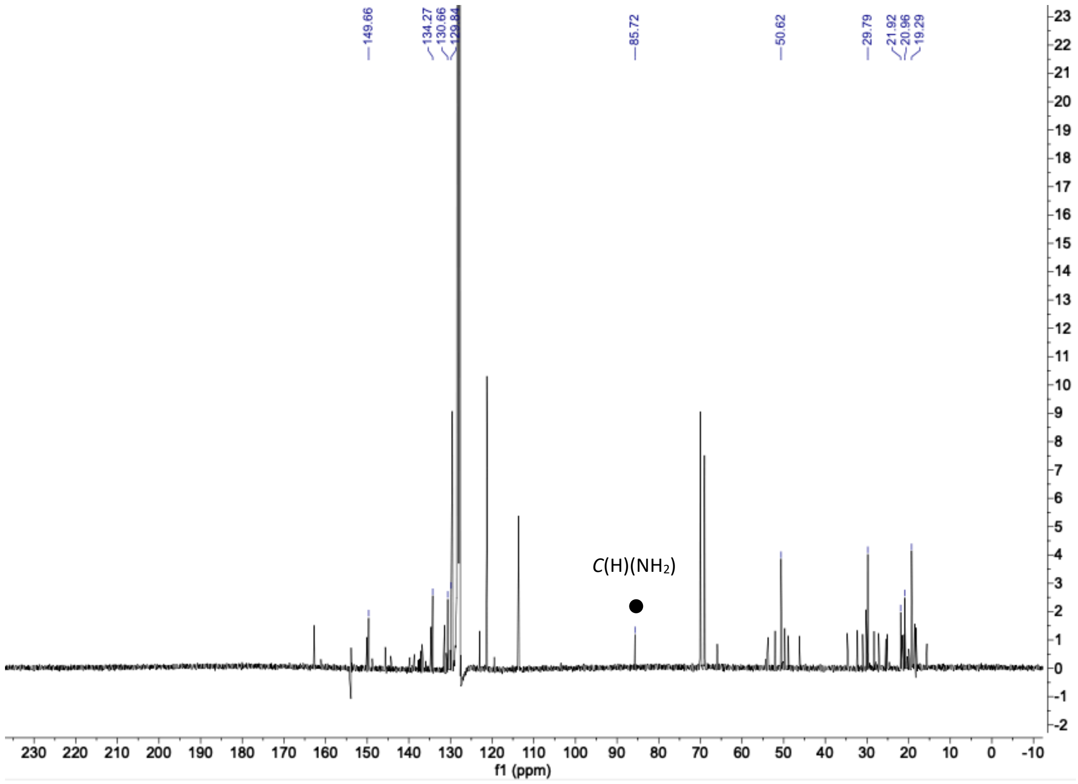

149.66
134.27
130.66
129.84
85.72
50.62
29.79
21.92
20.96
19.29
C(H)(NH2)
f1 (ppm)

D. Synthesis of NHC-Selenium Adducts

D.1. NHC-8.HBr with Selenium (4a)

In a 20 mL scintillation vial equipped with mini magnetic stir bar, NHC-8.HBr (51 mg, 1.0 eq), KHMDS (22 mg, 1.1 eq), selenium (15 mg, 2.0 eq), and THF (2.0 mL) were added. The solution was stirred overnight. The solution was filtered and all volatiles removed under vacuum. The resulting solid was redissolved in C_6D_6 and the solution allowed to slowly evaporate to give off-white crystals. NMR shows presence of HHMDS and minor amounts of NHC-8^H, likely from some hydrolysis.

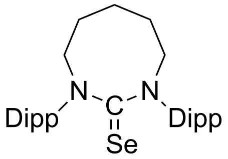

NHC-8(Se)

<u>NHC-8(Se) Adduct:</u> ^{1}H NMR (500 MHz, C_6D_6) δ = 7.24 (t, 2H, J=6.9Hz), 7.20 (m, 2H), 7.14 (d, 2H, J=6.9Hz), 3.61 (m, 4H), 3.24 (sept, 4H, J=7.0Hz), 1.70 (m, 4H), 1.59 (d, 12H, J=7.0Hz), 1.34 (m, 2H) 1.32 (d, 12H, J=7.0Hz). ^{13}C{^{1}H} NMR (125 MHz, C_6D_6) δ = 186.6, 147.5, 144.6, 127.8, 124.9, 55.6, 29.5, 26.1, 24.9, 24.7. ^{77}Se NMR (76.27 MHz, C_6D_6): 571.7.

^{1}H

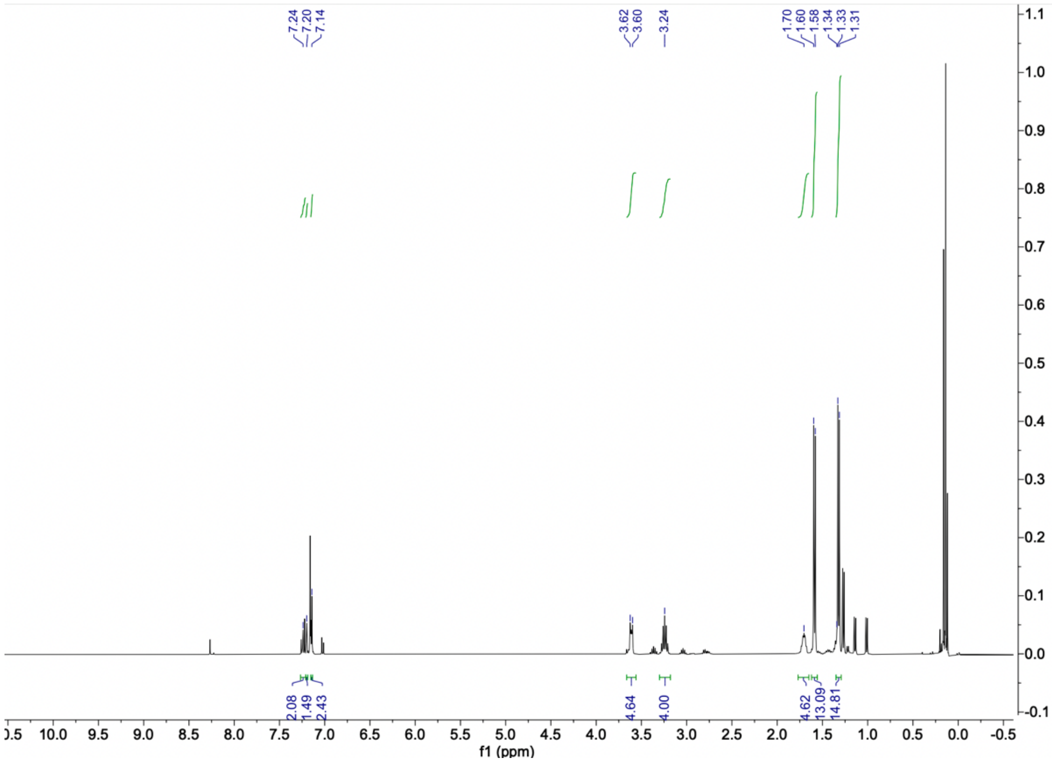

$^{13}C\{^{1}H\}$

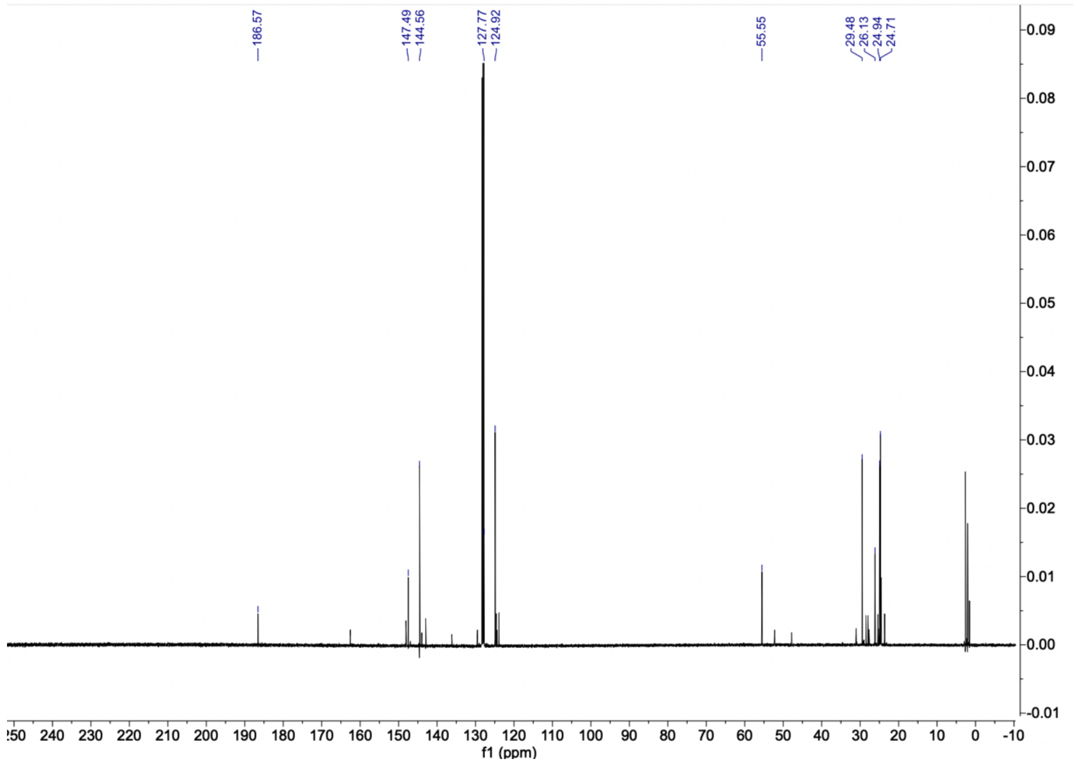
186.57
147.49
144.56
127.77
124.92
55.55
29.48
26.13
24.94
24.71
f1 (ppm)

^{77}Se

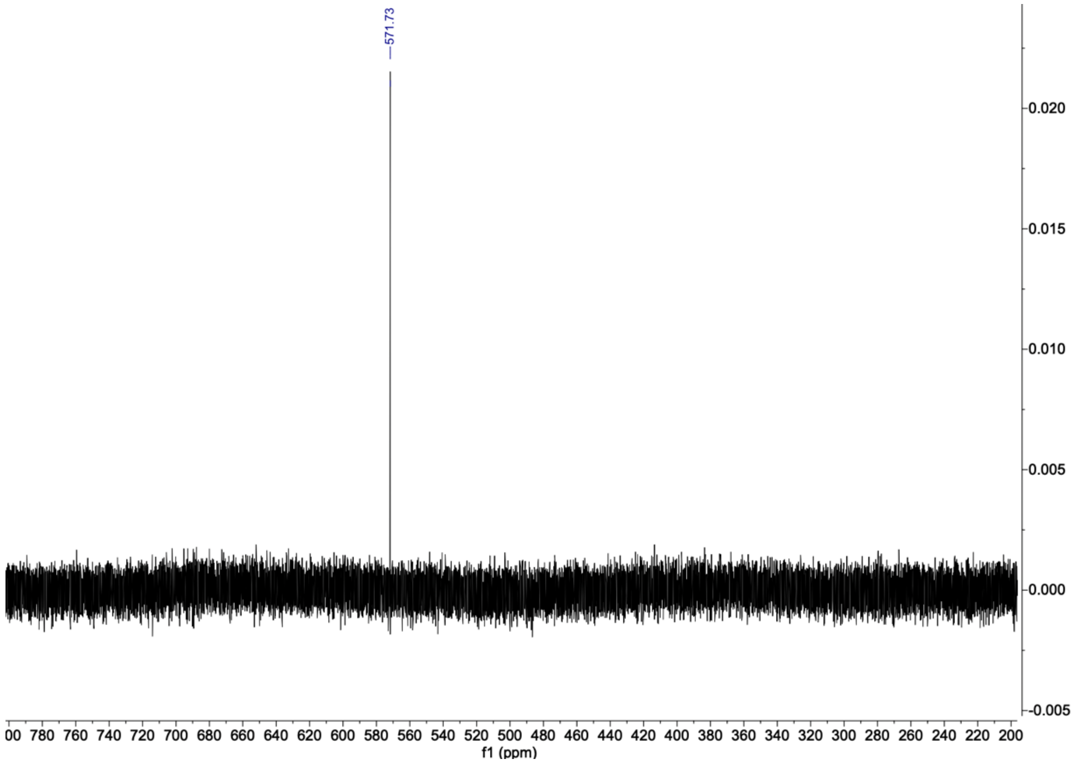
571.73
f1 (ppm)

D.2. ^{Mes}NHC-8.HBr with Selenium (4b)

In a 20 mL scintillation vial equipped with mini magnetic stir bar, ^{Mes}NHC-8.HBr (43 mg, 1.0 eq), KHMDS (22 mg, 1.1 eq), selenium (15 mg, 2.0 eq), and THF (2.0 mL) were added. The solution was stirred overnight. The solution was filtered and all volatiles removed under vacuum. The resulting solid was redissolved in C_6D_6 and the solution allowed to slowly evaporate to give off-white crystals.

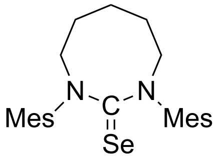

^{Mes}NHC-8(Se)

<u>^{Mes}NHC-8(Se) Adduct:</u> ^{1}H NMR (500 MHz, C_6D_6) δ = 6.82 (s, 4H), 3.40 (m, 4H), 2.34 (s, 12H), 2.15 (s, 6H), 1.63 (m, 4H), 1.29 (m, 2H). ^{13}C{^{1}H} NMR (125 MHz, C_6D_6) δ = 183.9, 147.2, 136.0, 134.6, 130.2, 54.2, 26.2, 24.1, 21.1, 19.5. ^{77}Se NMR (76.27 MHz, C_6D_6): 437.9.

^{1}H

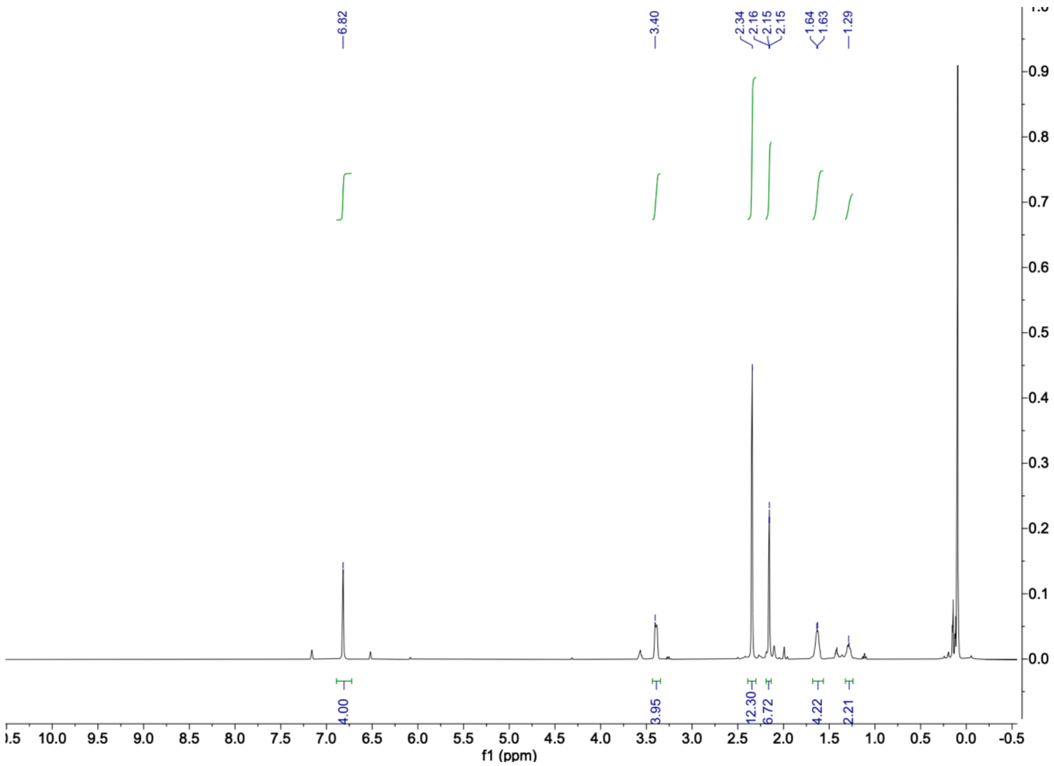

$^{13}C\{^1H\}$

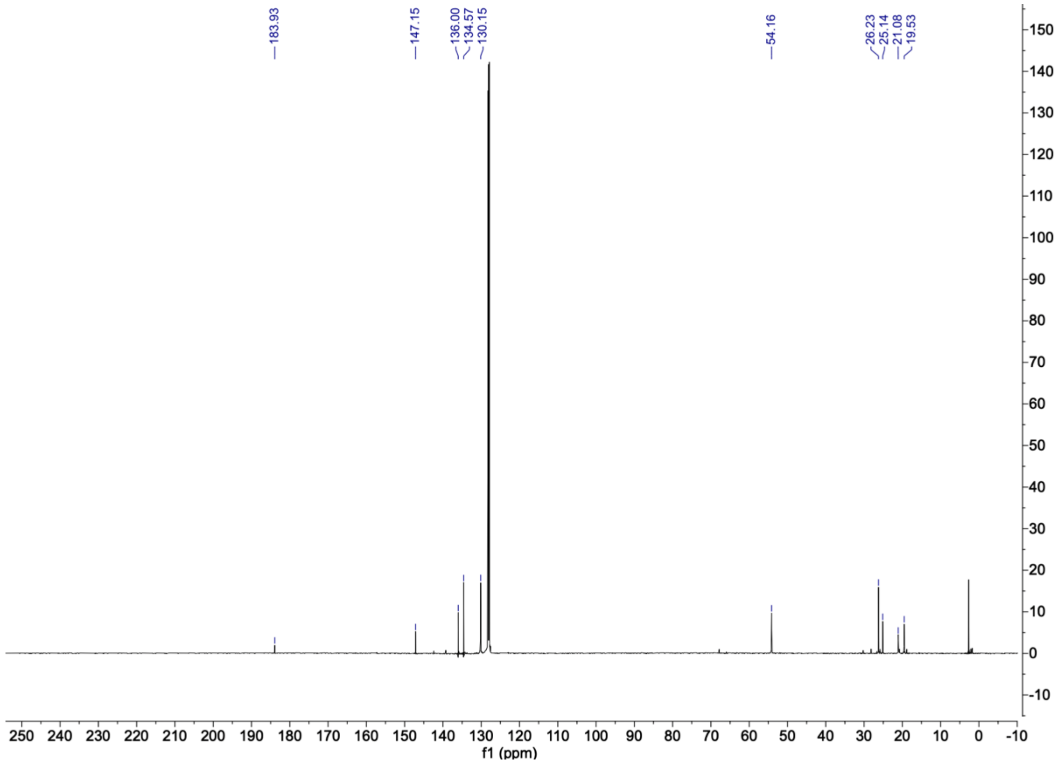
183.93
147.15
136.00
134.57
130.15
54.16
26.23
25.14
21.08
19.53
f1 (ppm)

^{77}Se

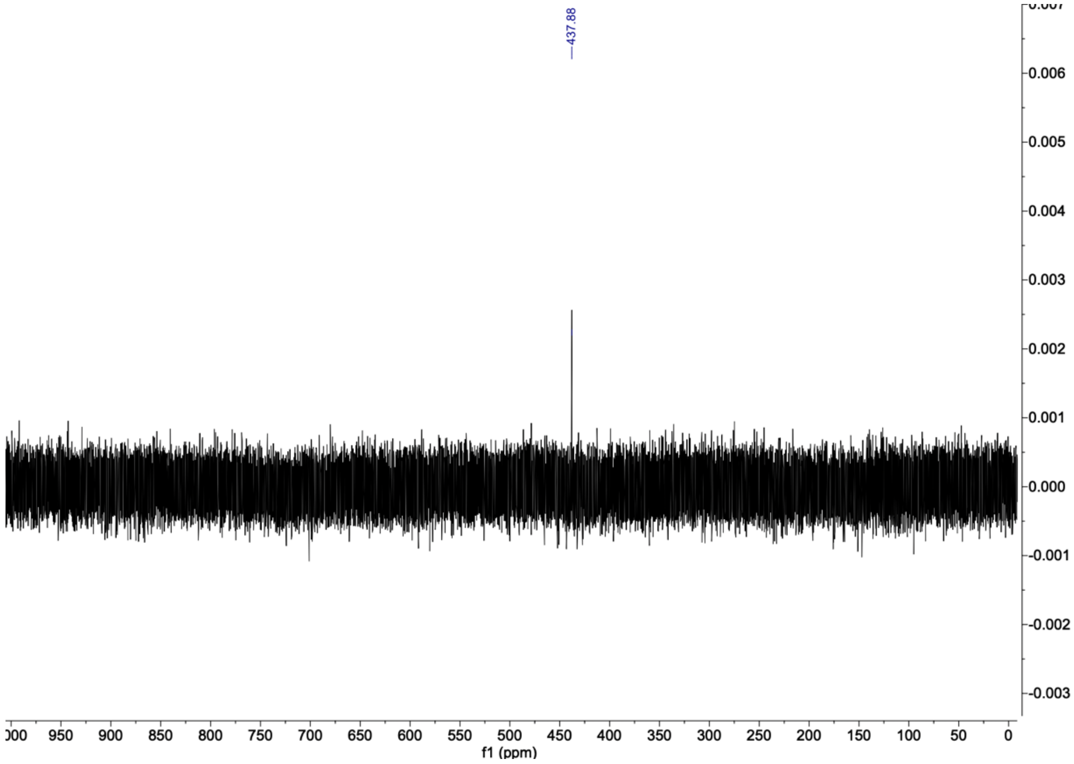
437.88
f1 (ppm)

E. Reactions Between NHC.HBr and $NaNH_2$

E.1. NHC6.HBr and $NaNH_2$ with *dibenzo*-18-crown-6.

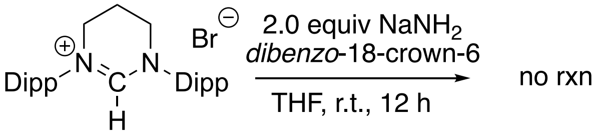

To a 20 mL scintillation vial equipped with a mini magnetic stir bar, NHC6.HBr (48.5 mg, 0.1 mmol), *dibenzo*-18-crown-6 (36.0 mg, 0.1 mmol), $NaNH_2$ (8.0 mg, 0.2 mmol), and THF (2 mL) were added in the glovebox. The vial was capped and the reaction was stirred for 12 hours. The solution was filtered and transferred to an NMR tube and a decoupled ^{13}C-NMR spectrum was taken to evaluate the disappearance of the amidinium peak and the appearance of a peak around 80-90 ppm. Minor carbene formation was observed.

^{13}C (125 MHz, THF)

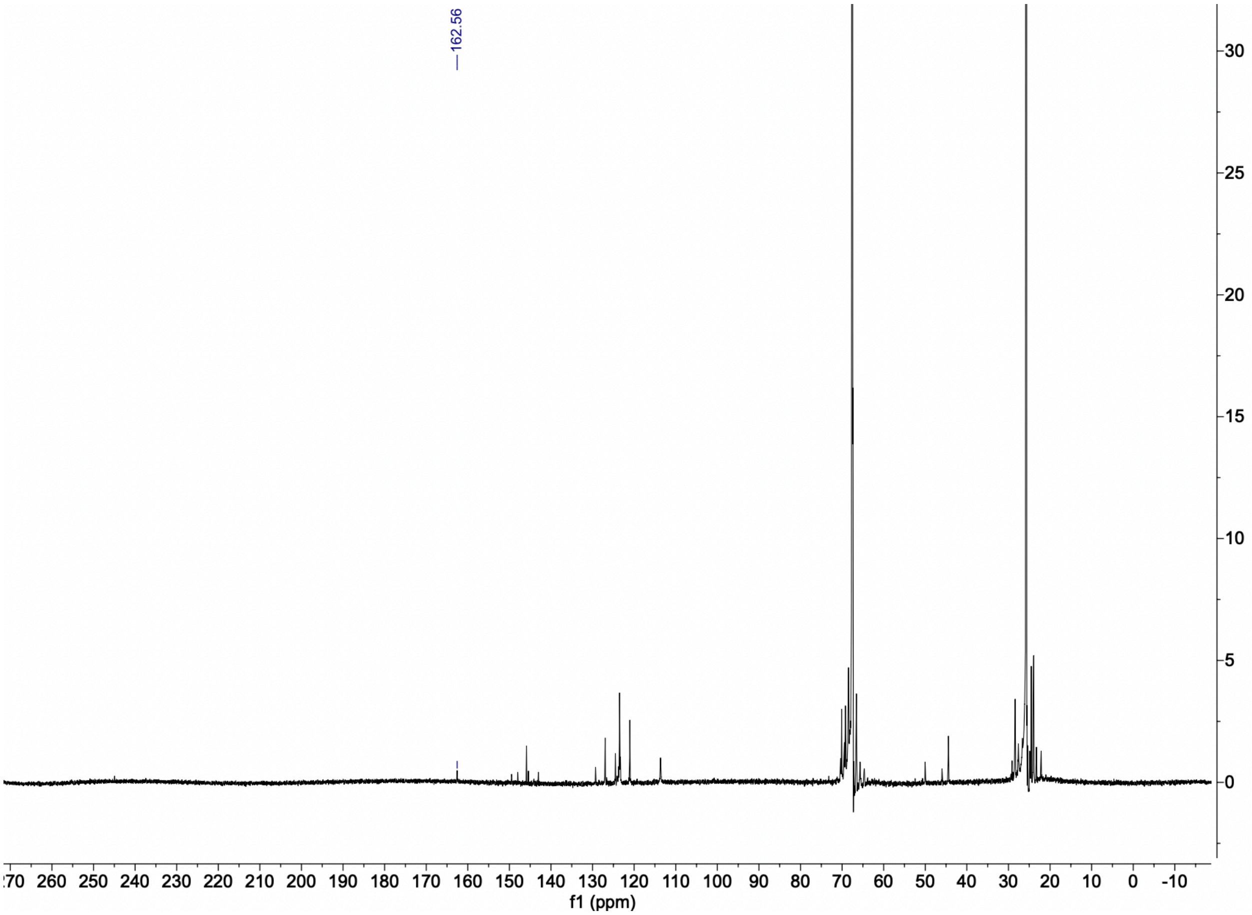

E.2. NHC6.HBr and $NaNH_2$ with *dibenzo*-18-crown-6.

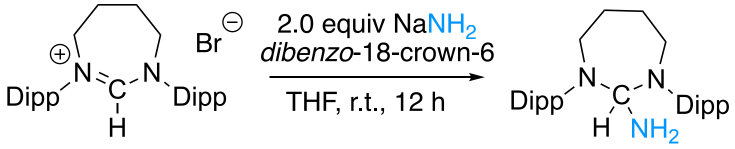

To a 20 mL scintillation vial equipped with a mini magnetic stir bar, NHC7.HBr (50.0 mg, 0.1 mmol), *dibenzo*-18-crown-6 (36.0 mg, 0.1 mmol), $NaNH_2$ (8.0 mg, 0.2 mmol), and THF (2 mL) were added in the glovebox. The vial was capped and the reaction was stirred for 12 hours. The solution was filtered and transferred to an NMR tube and a decoupled ^{13}C-NMR spectrum was taken to evaluate the disappearance of the amidinium peak and the appearance of a peak around 80-90 ppm.

^{13}C (125 MHz, THF)

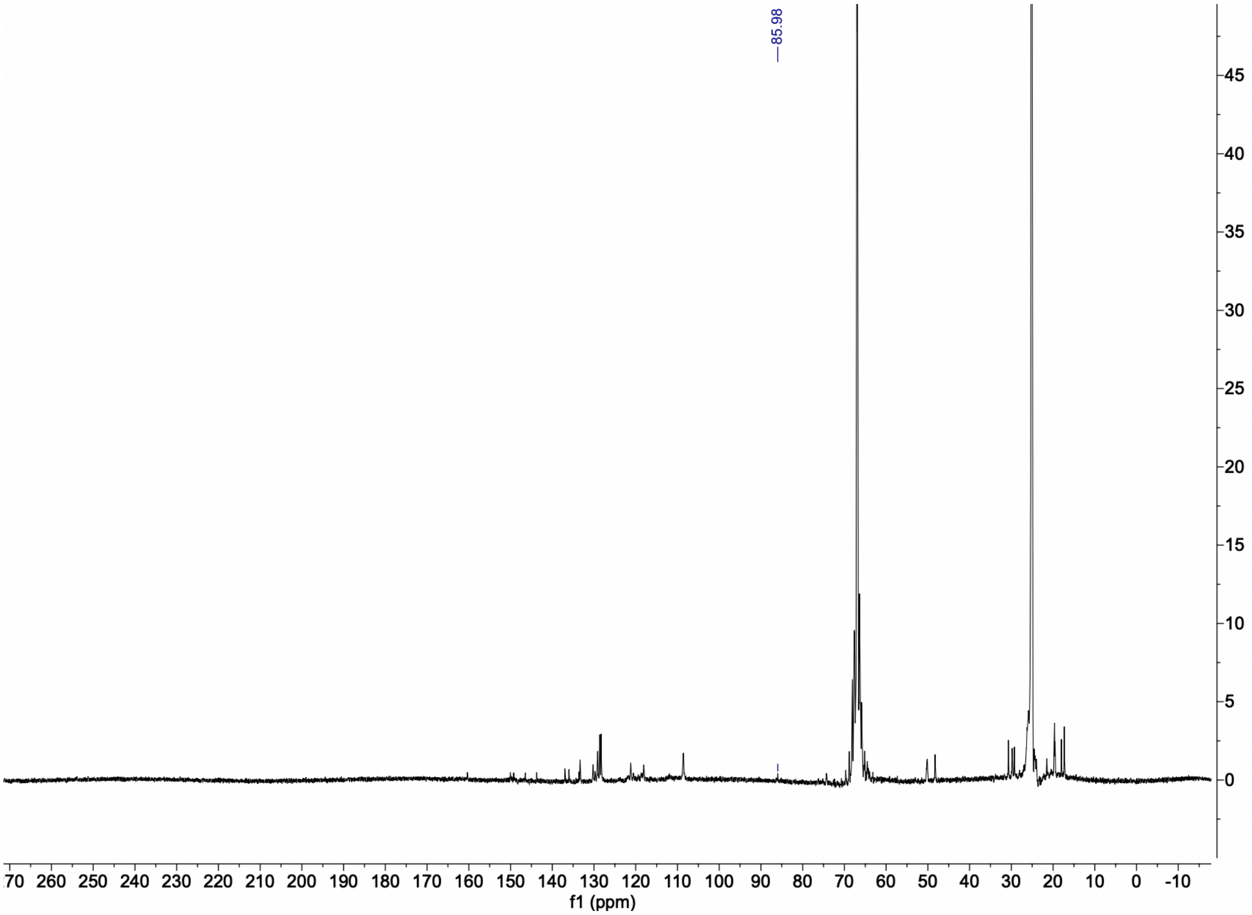

E.3. NHC8.HBr and $NaNH_2$ with *dibenzo*-18-crown-6.

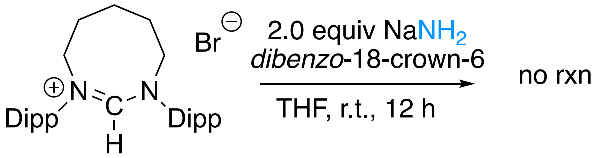

To a 20 mL scintillation vial equipped with a mini magnetic stir bar, NHC8.HBr (51.3 mg, 0.1 mmol), *dibenzo*-18-crown-6 (36.0 mg, 0.1 mmol), $NaNH_2$ (8.0 mg, 0.2 mmol), and THF (2 mL) were added in the glovebox. The vial was capped and the reaction was stirred for 12 hours. The solution was filtered and transferred to an NMR tube and a decoupled ^{13}C-NMR spectrum was taken to evaluate the disappearance of the amidinium peak and the appearance of a peak around 80-90 ppm. Minor carbene formation was observed.

^{13}C (125 MHz, THF)

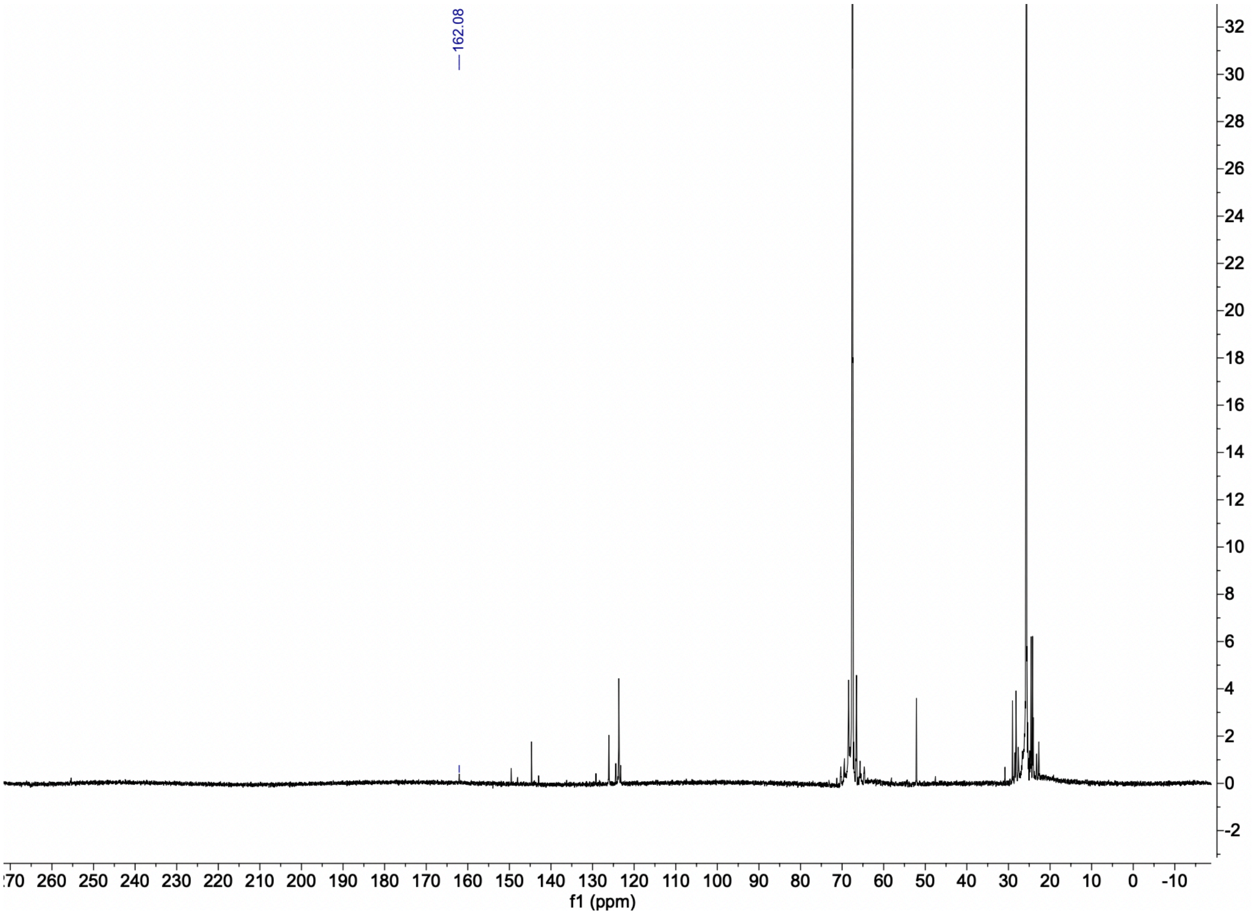

E.4. NHC8.HBr and $NaNH_2$ with *dibenzo*-18-crown-6.

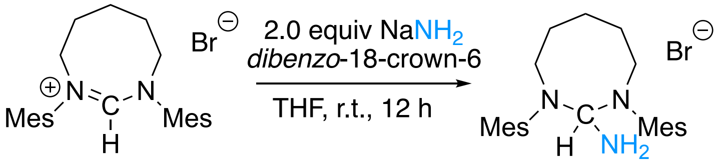

To a 20 mL scintillation vial equipped with a mini magnetic stir bar, ^{Mes}NHC8.HBr (43.0 mg, 0.1 mmol), *dibenzo*-18-crown-6 (36.0 mg, 0.1 mmol), $NaNH_2$ (8.0 mg, 0.2 mmol), and C_6D_6 (2 mL) were added in the glovebox. The vial was capped and the reaction was stirred for 12 hours. The solution was filtered and transferred to an NMR tube and a decoupled ^{13}C-NMR spectrum was taken to evaluate the disappearance of the amidinium peak and the appearance of a peak around 80-90 ppm.

^{13}C (125 MHz, C_6D_6)

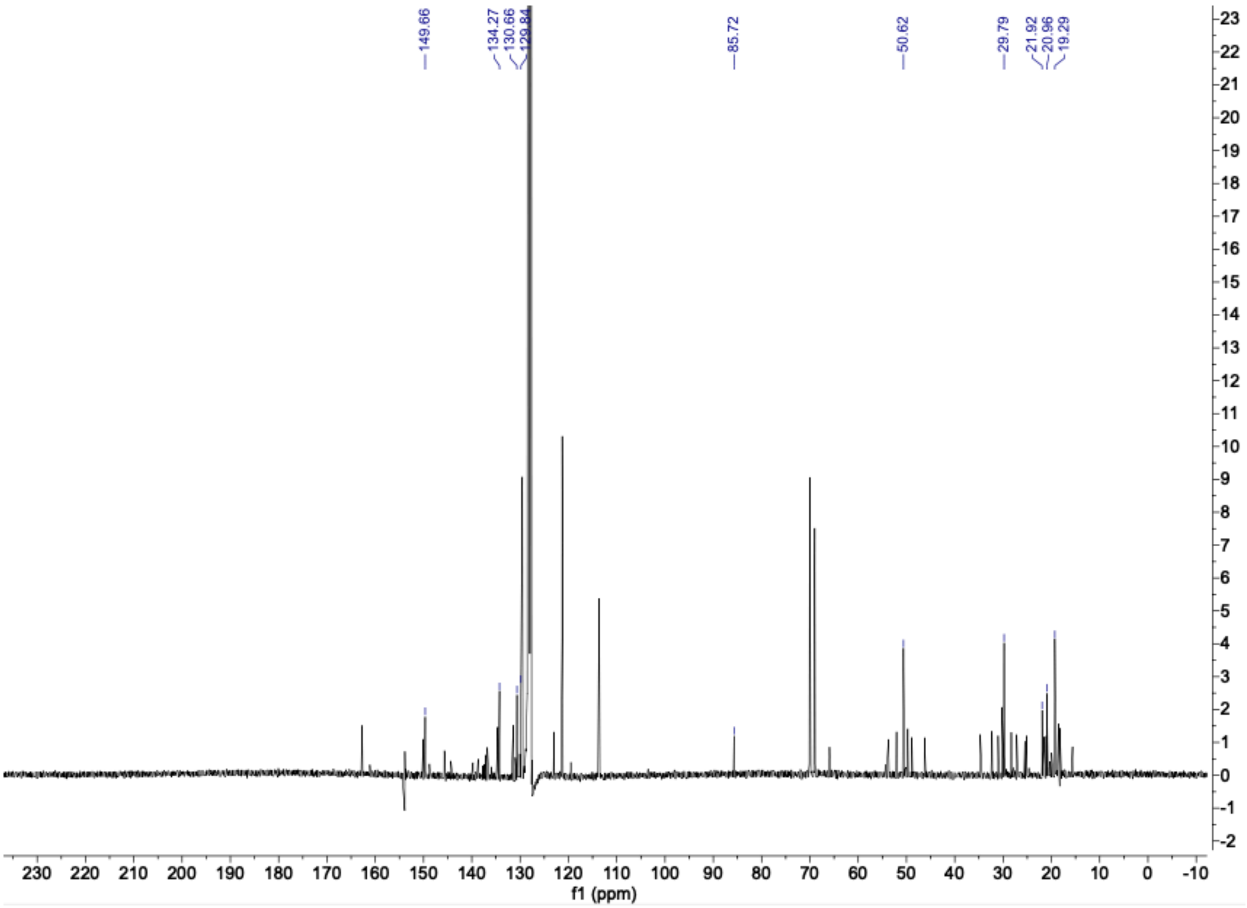

IV. X-Rays Structures

IV.1 X-Ray crystal structure determination

Olex2 software[5] was used for the resolution, refinement, and generation of crystallographic information files of every structure. The structures were solved with the ShelXS9[6] structure solution program using Direct Methods and refined with the ShelXL9[6] refinement package using Least Squares minimization. During the final stages of the refinements, all the positional parameters and the anisotropic temperature factors of all the non-H atoms were refined. The H atoms were geometrically located and their coordinates were refined riding on their parent atoms.

IV.2 X-Ray crystal structure determination for Compound 1c (CCDC 2291449)

Empirical formula	$C_{38}H_{50}N_2$
Formula weight	534.80
Temperature/K	100.0
Crystal system	triclinic
Space group	P-1
a/Å	9.9626(2)
b/Å	12.7843(2)
c/Å	13.6552(2)
α/°	69.2430(10)
β/°	75.6740(10)
γ/°	87.5200(10)
Volume/Å^3	1573.84(5)
Z	2
ρ_{calc}g/cm^3	1.129
μ/mm^{-1}	0.482
F(000)	584.0
Crystal size/mm^3	0.05 × 0.05 × 0.04
Radiation	CuKα (λ = 1.54178)
2Θ range for data collection/°	7.146 to 136.57
Index ranges	-11 ≤ h ≤ 11, -15 ≤ k ≤ 15, -16 ≤ l ≤ 16
Reflections collected	17967
Independent reflections	5563 [R_{int} = 0.0422, R_{sigma} = 0.0425]
Data/restraints/parameters	5563/0/370
Goodness-of-fit on F^2	1.036
Final R indexes [I>=2σ (I)]	R_1 = 0.0397, wR_2 = 0.0965
Final R indexes [all data]	R_1 = 0.0486, wR_2 = 0.1023
Largest diff. peak/hole / e Å^{-3}	0.22/-0.21

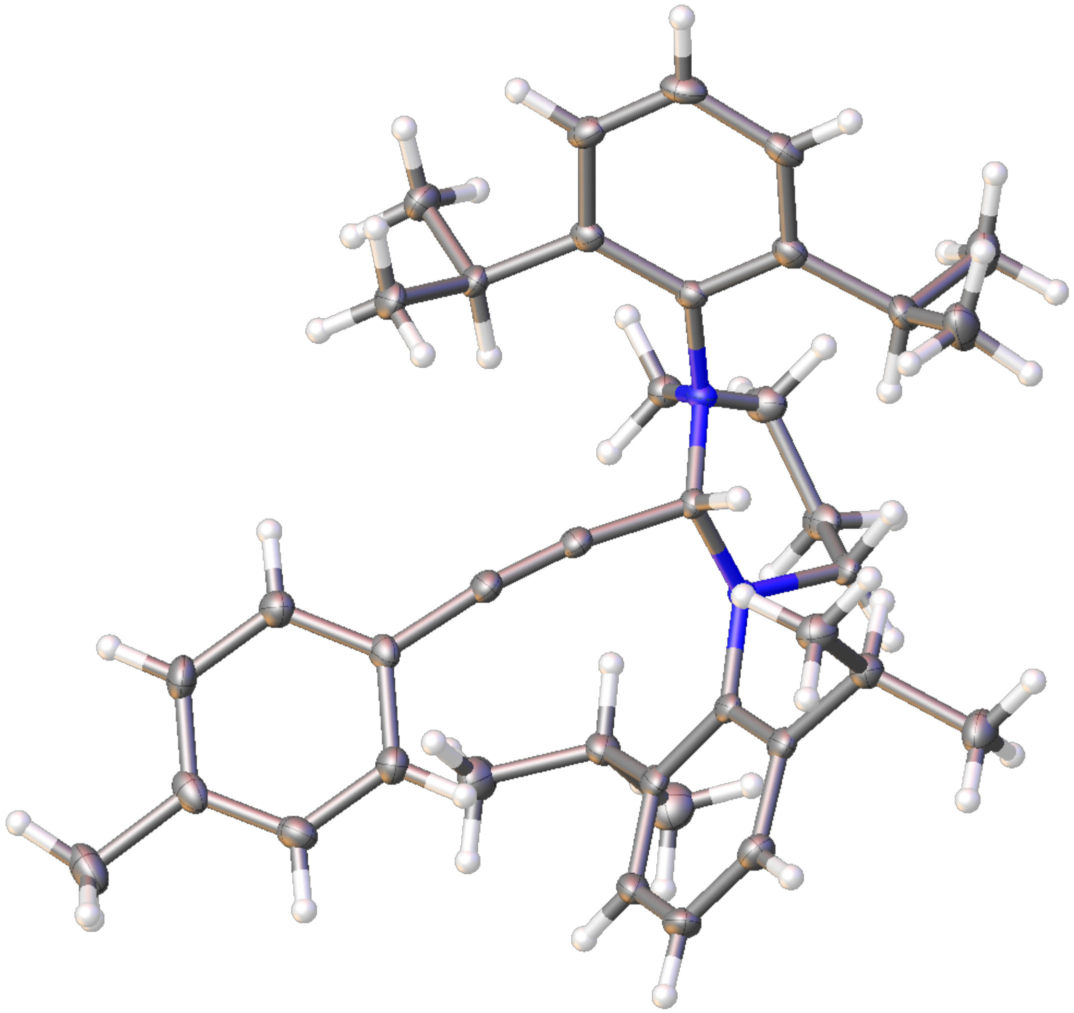

IV.3 X-Ray crystal structure determination for Compound 1d (CCDC 2291455)

Table 1 Crystal data and structure refinement for 1d.

Identification code	1d
Empirical formula	$C_{39}H_{52}N_2$
Formula weight	548.82
Temperature/K	100
Crystal system	triclinic
Space group	P-1
a/Å	10.2400(3)
b/Å	12.6553(3)
c/Å	13.8707(4)
α/°	68.0400(10)
β/°	75.9920(10)
γ/°	87.3960(10)
Volume/Å^3	1615.48(8)
Z	2
ρ_{calc}g/cm^3	1.128
μ/mm^{-1}	0.064
F(000)	600.0
Crystal size/mm^3	0.025 × 0.012 × 0.01
Radiation	MoKα (λ = 0.71073)
2Θ range for data collection/°	5.1 to 50.754
Index ranges	-12 ≤ h ≤ 12, -15 ≤ k ≤ 15, -16 ≤ l ≤ 16
Reflections collected	54670
Independent reflections	5923 [R_{int} = 0.0694, R_{sigma} = 0.0331]
Data/restraints/parameters	5923/0/379
Goodness-of-fit on F^2	1.018
Final R indexes [I>=2σ (I)]	R_1 = 0.0397, wR_2 = 0.0911
Final R indexes [all data]	R_1 = 0.0523, wR_2 = 0.0978
Largest diff. peak/hole / e Å^{-3}	0.19/-0.22

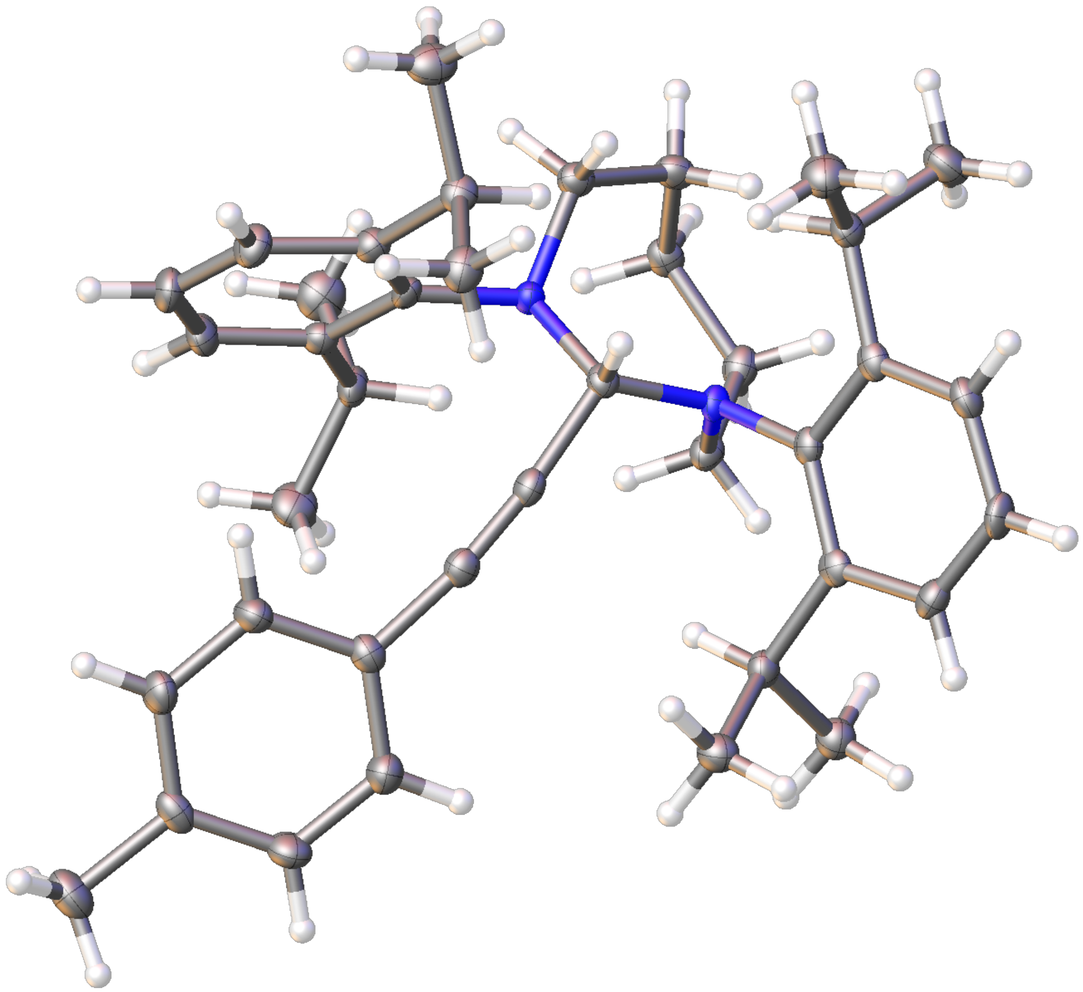

IV.4 X-Ray crystal structure determination for Compound 2b (CCDC 2291453)

Empirical formula	$C_{46}H_{63}N_3$
Formula weight	657.99
Temperature/K	100.00
Crystal system	monoclinic
Space group	$P2_1/c$
a/Å	20.277(3)
b/Å	10.5458(12)
c/Å	18.401(2)
α/°	90
β/°	92.839(6)
γ/°	90
Volume/Å^3	3930.0(8)
Z	4
ρ_{calc}g/cm^3	1.112
μ/mm^{-1}	0.478
F(000)	1440.0
Crystal size/mm^3	0.23 × 0.15 × 0.01
Radiation	CuKα (λ = 1.54178)
2Θ range for data collection/°	4.364 to 144.908
Index ranges	-24 ≤ h ≤ 24, -12 ≤ k ≤ 12, -22 ≤ l ≤ 22
Reflections collected	64029
Independent reflections	7157 [R_{int} = 0.1170, R_{sigma} = 0.0793]
Data/restraints/parameters	7157/0/450
Goodness-of-fit on F^2	1.089
Final R indexes [I>=2σ (I)]	R_1 = 0.0714, wR_2 = 0.1465
Final R indexes [all data]	R_1 = 0.0986, wR_2 = 0.1601
Largest diff. peak/hole / e Å^{-3}	0.34/-0.34

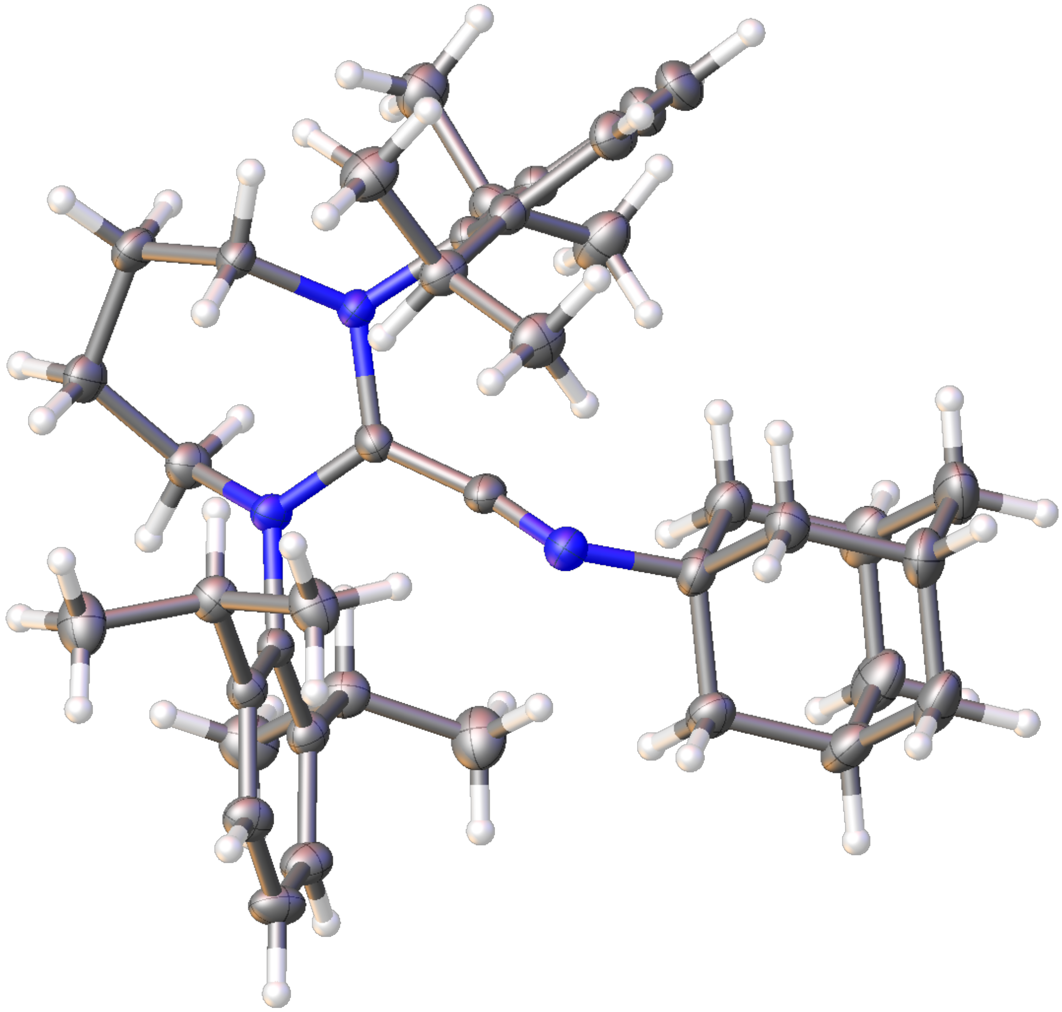

IV.5 X-Ray crystal structure determination for Compound 2c (CCDC 2291452)

Empirical formula	$C_{47}H_{65}N_3$
Formula weight	672.02
Temperature/K	273.15
Crystal system	monoclinic
Space group	$P2_1/c$
a/Å	10.1396(4)
b/Å	20.3171(8)
c/Å	19.4251(7)
α/°	90
β/°	100.0250(10)
γ/°	90
Volume/Å^3	3940.6(3)
Z	4
ρ_{calc}g/cm^3	1.133
μ/mm^{-1}	0.065
F(000)	1472.0
Crystal size/mm^3	0.024 × 0.023 × 0.01
Radiation	MoKα (λ = 0.71073)
2Θ range for data collection/°	5.312 to 51.448
Index ranges	$-12 \le h \le 12$, $-24 \le k \le 24$, $-23 \le l \le 23$
Reflections collected	55840
Independent reflections	7503 [R_{int} = 0.0799, R_{sigma} = 0.0446]
Data/restraints/parameters	7503/2/480
Goodness-of-fit on F^2	1.026
Final R indexes [I>=2σ (I)]	R_1 = 0.0441, wR_2 = 0.0961
Final R indexes [all data]	R_1 = 0.0665, wR_2 = 0.1063
Largest diff. peak/hole / e Å^{-3}	0.21/-0.28

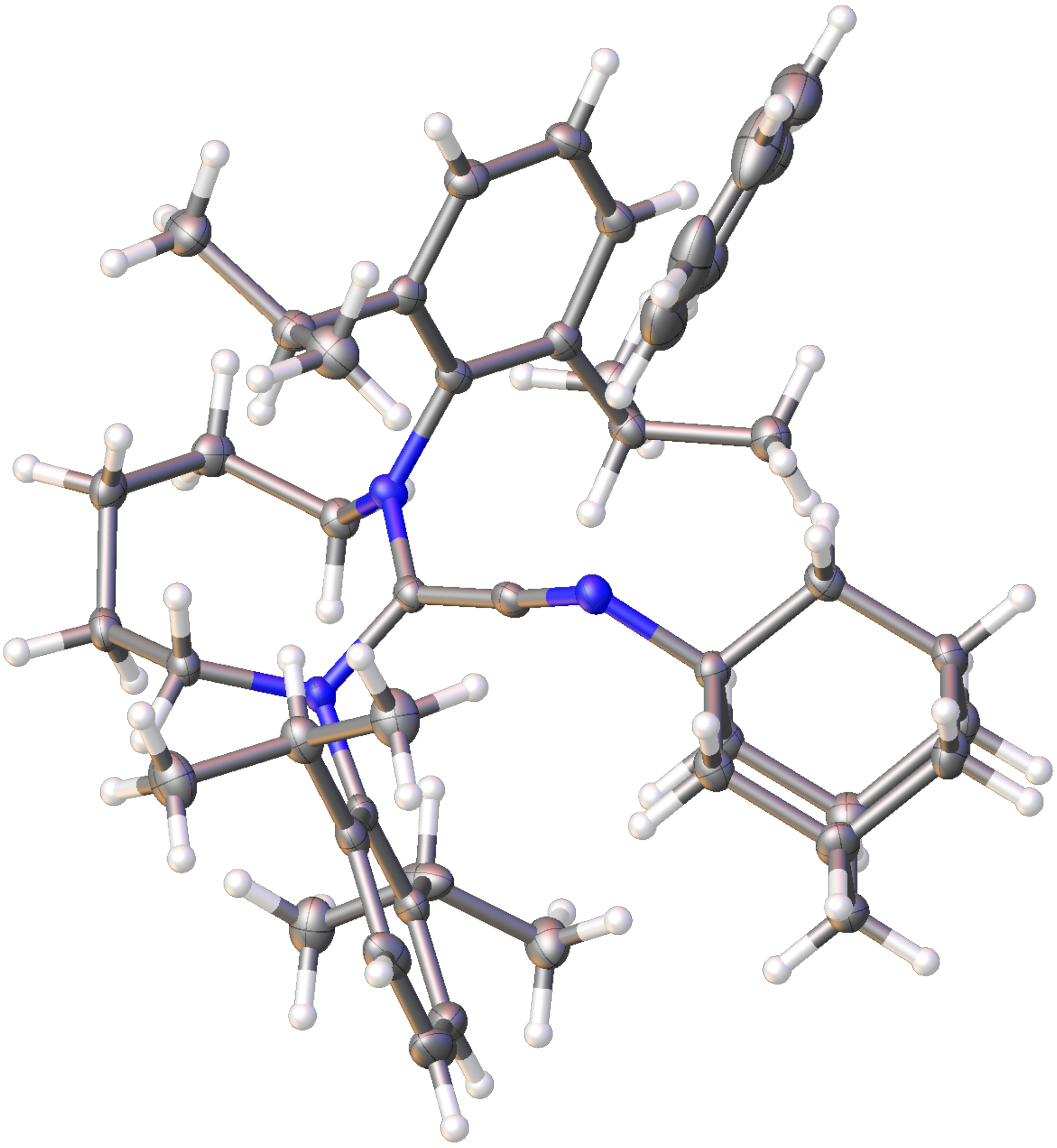

IV.6 X-Ray crystal structure determination for Compound 3b (CCDC 2291450)

Empirical formula	$C_{29}H_{45}N_3$
Formula weight	435.68
Temperature/K	100.15
Crystal system	monoclinic
Space group	$P2_1/c$
a/Å	10.4427(9)
b/Å	24.913(2)
c/Å	11.5295(9)
α/°	90
β/°	116.382(4)
γ/°	90
Volume/Å^3	2687.2(4)
Z	4
ρ_{calc}g/cm^3	1.077
μ/mm^{-1}	0.470
F(000)	960.0
Crystal size/mm^3	0.04 × 0.021 × 0.01
Radiation	CuKα (λ = 1.54178)
2Θ range for data collection/°	9.268 to 138.388
Index ranges	-12 ≤ h ≤ 12, -29 ≤ k ≤ 30, -13 ≤ l ≤ 13
Reflections collected	25294
Independent reflections	4946 [R_{int} = 0.0628, R_{sigma} = 0.0432]
Data/restraints/parameters	4946/0/305
Goodness-of-fit on F^2	1.032
Final R indexes [I>=2σ (I)]	R_1 = 0.0479, wR_2 = 0.1204
Final R indexes [all data]	R_1 = 0.0645, wR_2 = 0.1317
Largest diff. peak/hole / e Å^{-3}	0.42/-0.22

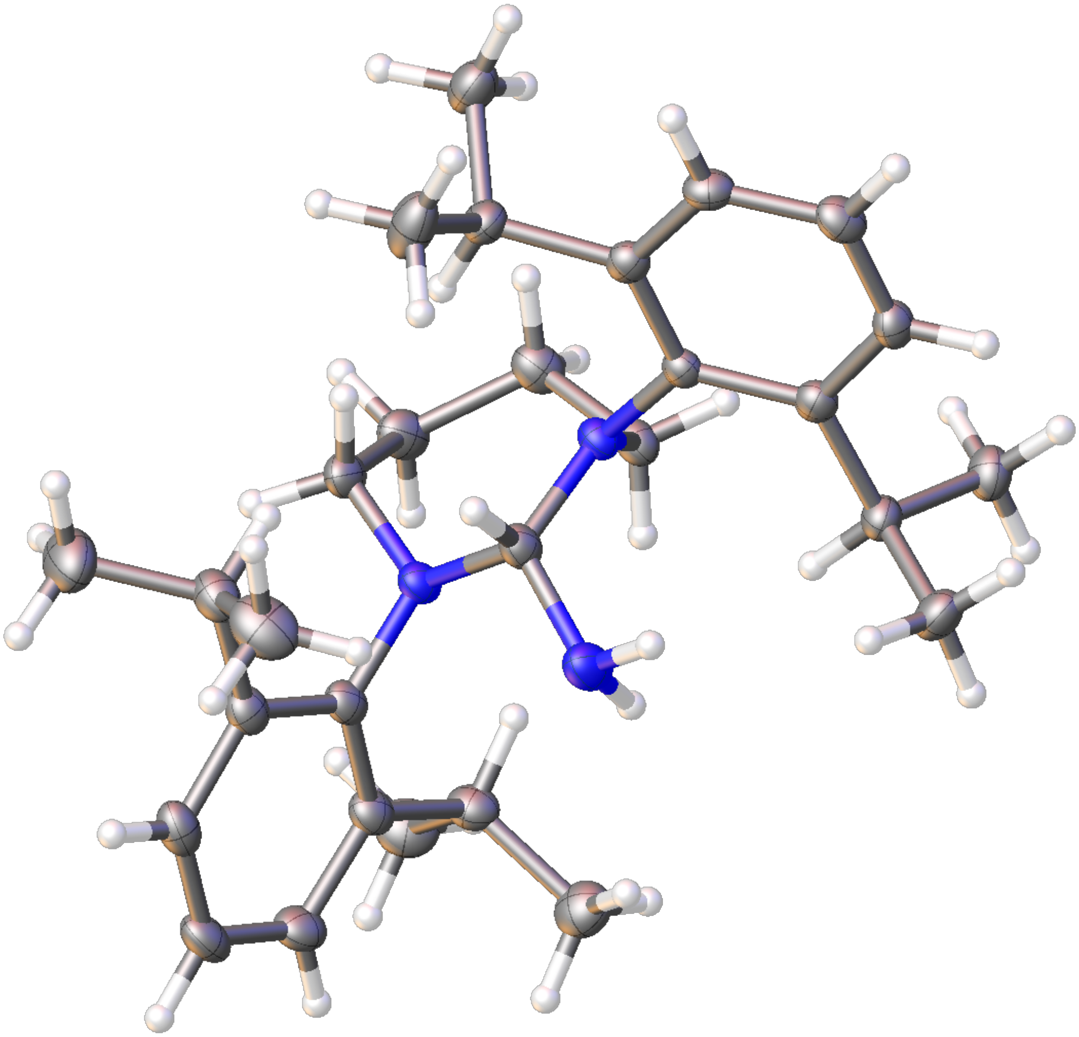

IV.7 X-Ray crystal structure determination for Compound 4a (CCDC 2291454)

Empirical formula	$C_{36}H_{50}N_2Se$
Formula weight	589.74
Temperature/K	273.15
Crystal system	monoclinic
Space group	$P2_1/c$
a/Å	15.9260(9)
b/Å	15.8347(8)
c/Å	14.3550(8)
α/°	90
β/°	116.049(2)
γ/°	90
Volume/Å^3	3252.4(3)
Z	4
ρ_{calc}g/cm^3	1.204
μ/mm^{-1}	1.180
F(000)	1256.0
Crystal size/mm^3	0.03 × 0.02 × 0.01
Radiation	MoKα (λ = 0.71073)
2Θ range for data collection/°	5.676 to 50.758
Index ranges	-19 ≤ h ≤ 19, -19 ≤ k ≤ 19, -17 ≤ l ≤ 17
Reflections collected	70350
Independent reflections	5970 [R_{int} = 0.0917, R_{sigma} = 0.0401]
Data/restraints/parameters	5970/0/360
Goodness-of-fit on F^2	1.023
Final R indexes [I>=2σ (I)]	R_1 = 0.0320, wR_2 = 0.0768
Final R indexes [all data]	R_1 = 0.0425, wR_2 = 0.0824
Largest diff. peak/hole / e Å^{-3}	0.42/-0.49

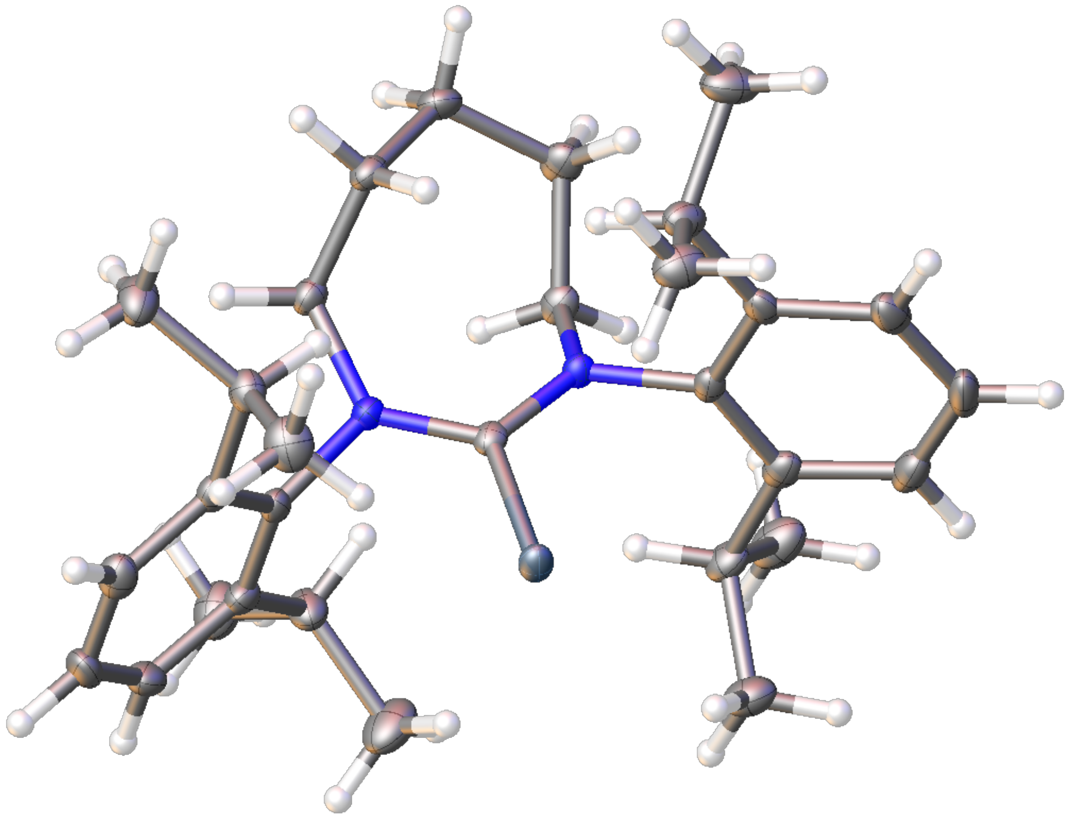

IV.8 X-Ray crystal structure determination for Compound 4b (CCDC 2291451)

Empirical formula	$C_{24}H_{32}N_2Se$
Formula weight	427.494
Temperature/K	100.00
Crystal system	monoclinic
Space group	C2/c
a/Å	19.8071(9)
b/Å	13.3759(7)
c/Å	17.5615(8)
α/°	90
β/°	110.970(2)
γ/°	90
Volume/Å^3	4344.5(4)
Z	8
ρ_{calc}g/cm^3	1.307
μ/mm^{-1}	1.739
F(000)	1792.7
Crystal size/mm^3	0.1 × 0.067 × 0.055
Radiation	Mo Kα (λ = 0.71073)
2Θ range for data collection/°	3.76 to 52.88
Index ranges	-24 ≤ h ≤ 24, -16 ≤ k ≤ 16, -21 ≤ l ≤ 21
Reflections collected	44841
Independent reflections	4459 [R_{int} = 0.0474, R_{sigma} = 0.0225]
Data/restraints/parameters	4459/0/250
Goodness-of-fit on F^2	1.037
Final R indexes [I>=2σ (I)]	R_1 = 0.0218, wR_2 = 0.0539
Final R indexes [all data]	R_1 = 0.0254, wR_2 = 0.0555
Largest diff. peak/hole / e Å^{-3}	0.34/-0.29

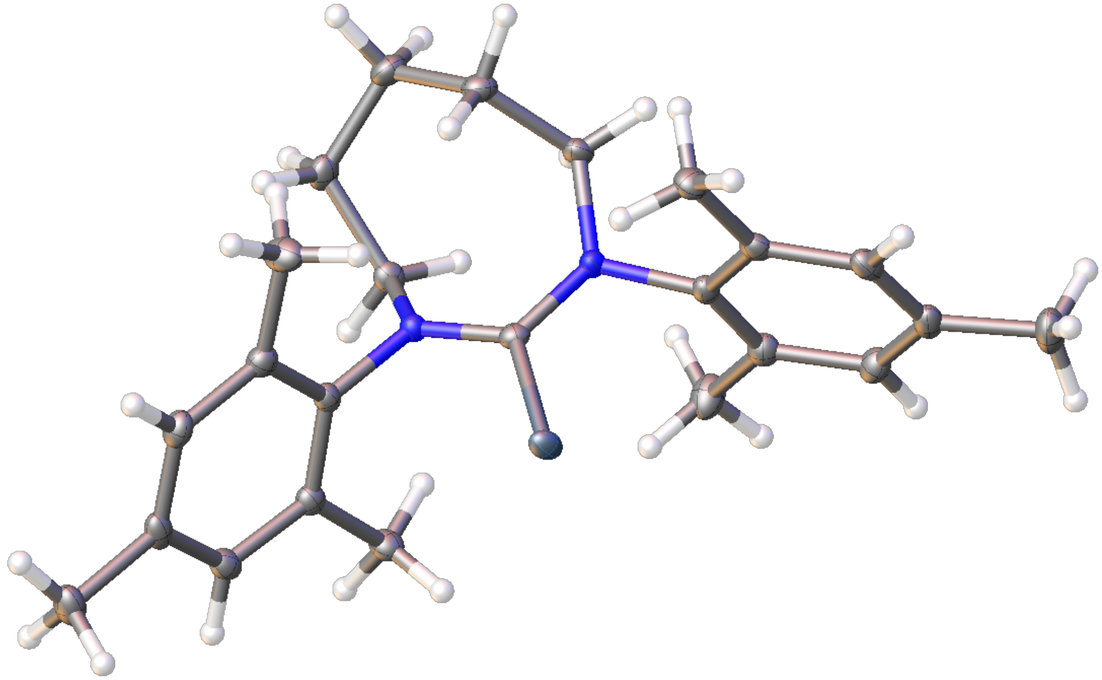

X. Computational Data

All calculations were performed with the Gaussian16 program package.[7] The theoretical approach is based on the framework of density functional theory (DFT). All calculations were performed with the B3LYP functional and employing Weigend and Ahlrich's def2-TZVPP basis set.[8] The calculations were benchmarked against the functional-basis set combinations of MO 62x//TZVP and B3LYP//6-31G(d,f). The singlet and triplet states of each carbene were fully optimized without constraints at the aforementioned level of theory. The Gibbs energy corrections from frequency calculations and dispersion corrections were added to the single-point energies to obtain the Gibbs free energies in solution. Gibbs free reaction energies and enthalpies were calculated for standard conditions (P = 1 atm, T = 298 K) and are unscaled and are given in the following Table S1-3. The $\%V_{bur}$ was calculated using the SambVca 2 program and measured at a distance of 0 Å from the carbene carbon.[9] Cartesian coordinates for the optimized structures are given in the following subsection.

Table S1. DFT Calculated Geometric and Thermodynamic Data of CAAC-5 and NHCs at the B3LYP//def2-TZVPP level.

Carbene	$\Delta E_{S\text{-}T}$ (kcal/mol)	Singlet LUMO (eV)<br>Singlet HOMO (eV)	$\Delta E_{H\text{-}L}$ (eV)	Carbene angle (°)
CAAC-5	45.72	-0.448<br>-5.290	4.842	107.3
NHC-5	73.08	-0.359<br>-5.805	5.446	106.3
NHC-6	59.89	-0.342<br>-5.322	4.980	116.0
NHC-7	50.02	-0.358<br>-5.103	4.745	118.7
NHC-8	49.29	-0.384<br>-5.110	4.726	122.1

Table S2. DFT Calculated Geometric and Thermodynamic Data of CAAC-5 and NHCs with MO62x//def2-TZVPP level.

Carbene	$\Delta E_{S\text{-}T}$ (kcal/mol)	Singlet LUMO (eV)<br>Singlet HOMO (eV)	$\Delta E_{H\text{-}L}$ (eV)	Carbene angle (°)
CAAC-5	45.72	-0.448<br>-5.290	4.842	107.3
NHC-5	73.08	-0.359<br>-5.805	5.446	106.3
NHC-6	59.89	-0.342<br>-5.322	4.980	116.0
NHC-7	50.02	-0.358<br>-5.103	4.745	118.7
NHC-8	49.29	-0.384<br>-5.110	4.726	122.1

Table S3. DFT Calculated Geometric and Thermodynamic Data of CAAC-5 and NHCs at the B3LYP//6-31G(d,f) level.

Carbene	ΔE_{S-T} (kcal/mol)	Singlet LUMO (eV)<br>Singlet HOMO (eV)	ΔE_{H-L} (eV)	Carbene angle (°)
CAAC-5	45.72	-0.448<br>-5.290	4.842	107.3
NHC-5	73.08	-0.359<br>-5.805	5.446	106.3
NHC-6	59.89	-0.342<br>-5.322	4.980	116.0
NHC-7	50.02	-0.358<br>-5.103	4.745	118.7
NHC-8	49.29	-0.384<br>-5.110	4.726	122.1

Optimized x,y,z-coordinates

CAAC-5 singlet

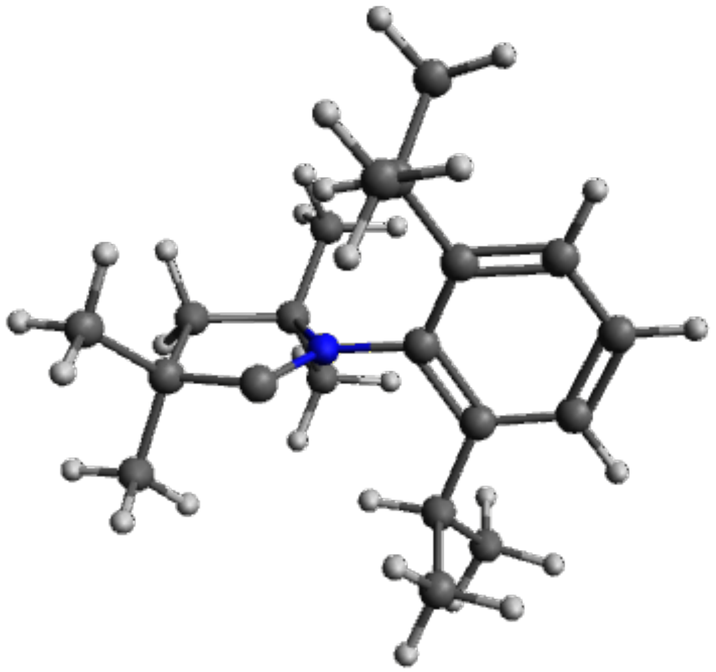

**Zero-point correction=**	**0.464716 (Hartree/Particle)**
**Thermal correction to Energy=**	**0.487947**
**Thermal correction to Enthalpy=**	**0.488891**
**Thermal correction to Gibbs Free Energy=**	**0.414529**
**Sum of electronic and zero-point Energies=**	**-835.349444**
**Sum of electronic and thermal Energies=**	**-835.326213**
**Sum of electronic and thermal Enthalpies=**	**-835.325268**
**Sum of electronic and thermal Free Energies=**	**-835.399631**

C	-1.33181 -0.07661 -1.04389	C	3.60801 0.19134 -0.01714
C	-2.78932 -0.17973 -0.62513	H	4.6885 0.2514 0.00031
C	-2.77351 -0.45341 0.90599	C	2.97987 -1.04264 -0.04801
H	-3.53022 0.12154 1.44005	H	3.58113 -1.94079 -0.08184
H	-2.97969 -1.50788 1.09509	C	1.58935 -1.1488 -0.06088
C	-1.3577 -0.10728 1.39875	C	0.67824 2.59354 -0.284
C	-3.48192 1.14972 -0.97893	H	-0.37121 2.39684 -0.08838
H	-3.39138 1.35431 -2.0455	C	1.11613 3.73555 0.64232
H	-3.04845 1.99363 -0.44094	H	1.06516 3.44832 1.69318
H	-4.54311 1.09923 -0.72493	H	0.46865 4.60231 0.49835
C	-3.46873 -1.31661 -1.40045	H	2.13733 4.05902 0.43625
H	-4.51296 -1.4183 -1.09431	C	0.77564 3.01604 -1.76094
H	-2.97073 -2.27107 -1.22188	H	1.80881 3.23099 -2.04044
H	-3.43897 -1.12106 -2.47214	H	0.18467 3.91718 -1.93791
C	-1.29707 1.24903 2.10908	H	0.40107 2.22679 -2.41196

H	-1.83488	1.17996	3.05576	C	0.95793	-2.52043	-0.2612
H	-1.75831	2.04109	1.52257	H	-0.10432	-2.43961	-0.04662
H	-0.27015	1.53552	2.33361	C	1.53471	-3.60418	0.65889
C	-0.7825	-1.16932	2.33561	H	0.98265	-4.53676	0.52895
H	0.25811	-0.96401	2.58707	H	1.47271	-3.31832	1.70942
H	-0.84755	-2.16796	1.90969	H	2.58067	-3.81615	0.43342
H	-1.354	-1.16886	3.2651	C	1.07372	-2.9342	-1.73928
C	0.83265	0.03796	-0.01111	H	2.1192	-3.0342	-2.03715
C	1.45256	1.30027	-0.06818	H	0.60329	-2.1931	-2.38484
C	2.84729	1.3474	-0.05274	H	0.58311	-3.89576	-1.90462
H	3.34537	2.30649	-0.09086	N	-0.61511	-0.05379	0.04614

CAAC-5 triplet

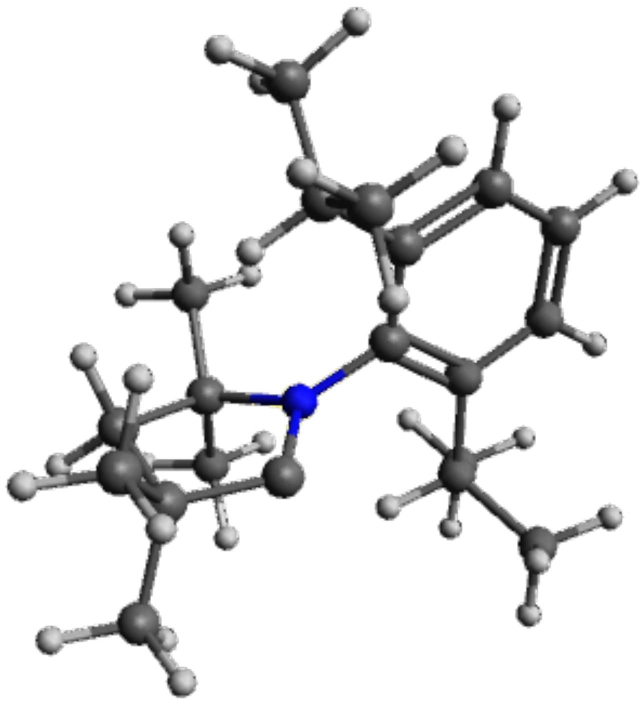

Zero-point correction= **0.462873 (Hartree/Particle)**
Thermal correction to Energy= **0.486544**
Thermal correction to Enthalpy= **0.487488**
Thermal correction to Gibbs Free Energy= **0.410338**
Sum of electronic and zero-point Energies= **-835.274239**
Sum of electronic and thermal Energies= **-835.250568**
Sum of electronic and thermal Enthalpies= **-835.249624**
Sum of electronic and thermal Free Energies= **-835.326773**

C	-1.28246	0.57811	-1.01322	C	3.34246	-1.4734	-0.17851
C	-2.74397	0.69528	-0.69859	H	4.31692	-1.9429	-0.21987
C	-2.6613	0.58515	0.86494	C	2.19864	-2.25051	-0.08743
H	-3.32187	1.30427	1.34988	H	2.29275	-3.32823	-0.07212

H	-2.98401	-0.41251	1.1649	C	0.93223	-1.67166	-0.02312
C	-1.1796	0.7902	1.30321	C	1.91949	2.04146	-0.37464
C	-3.3384	2.03947	-1.16005	H	0.88289	2.34083	-0.25223
H	-3.28477	2.12421	-2.24625	C	2.76028	2.80556	0.65797
H	-2.79864	2.88448	-0.735	H	2.48038	2.54273	1.67894
H	-4.38856	2.11945	-0.86687	H	2.62422	3.88194	0.53707
C	-3.59819	-0.44324	-1.28948	H	3.82535	2.59571	0.54542
H	-4.63551	-0.36749	-0.95194	C	2.32799	2.43158	-1.80531
H	-3.21386	-1.41962	-0.99616	H	3.36691	2.16713	-2.0112
H	-3.59755	-0.39476	-2.37932	H	2.22251	3.50872	-1.94984
C	-0.91082	2.26395	1.64922	H	1.70086	1.92866	-2.54164
H	-1.56337	2.57892	2.46591	C	-0.28945	-2.57805	-0.01069
H	-1.10033	2.91327	0.79591	H	-1.1541	-1.95757	0.21337
H	0.11895	2.41143	1.972	C	-0.21652	-3.6784	1.05765
C	-0.81616	-0.07431	2.50815	H	-1.1546	-4.23586	1.0879
H	0.24176	0.01719	2.75605	H	-0.03589	-3.26569	2.05076
H	-1.0393	-1.12498	2.33703	H	0.57898	-4.39508	0.84868
H	-1.39099	0.25556	3.37502	C	-0.52097	-3.1902	-1.40284
C	0.8299	-0.26165	-0.03277	H	0.31386	-3.83092	-1.69331
C	1.98531	0.53363	-0.18726	H	-0.62839	-2.41318	-2.15953
C	3.23	-0.09556	-0.2415	H	-1.42768	-3.79882	-1.40835
H	4.12407	0.5042	-0.35107	N	-0.45453	0.36062	0.06308

NHC-5 singlet

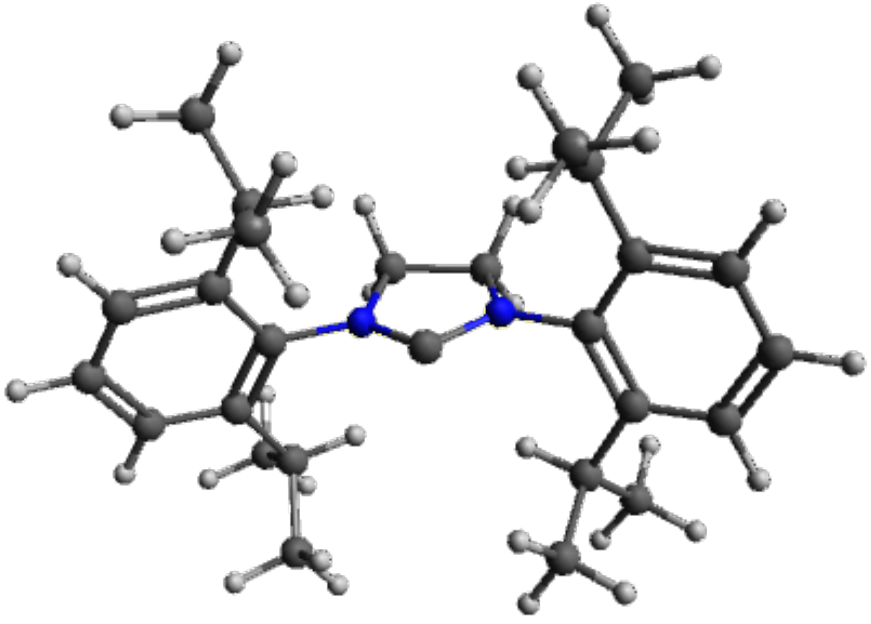

Zero-point correction= **0.590508 (Hartree/Particle)**

Thermal correction to Energy= **0.621778**

Thermal correction to Enthalpy= **0.622722**

Thermal correction to Gibbs Free Energy= 0.525672
Sum of electronic and zero-point Energies= -1161.080649
Sum of electronic and thermal Energies= -1161.049380
Sum of electronic and thermal Enthalpies= -1161.048436
Sum of electronic and thermal Free Energies= -1161.145486

N	-1.07505	-0.04006	0.51939
N	1.07504	0.04001	0.5194
C	0.	0.	-0.28662
C	-0.75778	-0.10656	1.96866
H	-1.04729	-1.07934	2.37413
H	-1.29704	0.66086	2.52391
C	0.75775	0.10649	1.96868
H	1.04727	1.07927	2.37417
H	1.297	-0.66095	2.52391
C	2.42854	0.15964	0.06903
C	2.92921	1.42229	-0.29752
C	4.25565	1.50767	-0.71977
H	4.6595	2.46584	-1.01855
C	5.06771	0.38567	-0.7675
H	6.09497	0.4737	-1.09708
C	4.56071	-0.84963	-0.39765
H	5.19971	-1.72159	-0.4473
C	3.23884	-0.98889	0.02412
C	2.70793	-2.36922	0.38028
H	1.68461	-2.24816	0.73223
C	3.51271	-3.02994	1.50942
H	3.54316	-2.40036	2.40004
H	4.54333	-3.22469	1.20839
H	3.06507	-3.98644	1.78591
C	2.64778	-3.27614	-0.85909
H	2.03648	-2.82724	-1.64169
H	2.21667	-4.24588	-0.60232
H	3.64358	-3.45277	-1.26966
C	2.06147	2.6714	-0.28529
H	1.13561	2.42563	0.23258
C	1.67683	3.08223	-1.7158
H	1.15442	2.27111	-2.22187
H	2.56226	3.33739	-2.30157
H	1.02195	3.95582	-1.70016
C	2.71339	3.838	0.47081
H	2.98552	3.55308	1.48827
H	2.02235	4.68097	0.52857
H	3.61705	4.19256	-0.02721
C	-2.42856	-0.15966	0.06904
C	-3.23884	0.98889	0.02416
C	-4.56072	0.84965	-0.39756
H	-5.1997	1.72163	-0.44722
C	-5.06776	-0.38564	-0.76738
H	-6.09504	-0.47366	-1.0969
C	-4.25572	-1.50766	-0.71969
H	-4.65961	-2.46581	-1.01845
C	-2.92926	-1.4223	-0.2975
C	-2.06151	-2.6714	-0.28531
H	-1.13567	-2.42564	0.23261
C	-2.71344	-3.83807	0.47063
H	-2.98551	-3.55332	1.48816
H	-2.02243	-4.68109	0.52823
H	-3.61713	-4.19252	-0.0274
C	-1.67679	-3.08212	-1.71585
H	-1.15434	-2.27098	-2.22183
H	-2.56219	-3.33724	-2.30168
H	-1.02192	-3.95572	-1.70024
C	-2.70789	2.36922	0.38025
H	-1.68459	2.24814	0.73227
C	-3.51268	3.03007	1.50931
H	-3.54315	2.40058	2.39999
H	-4.54329	3.22481	1.20824
H	-3.06503	3.98659	1.78571
C	-2.64763	3.27604	-0.85918
H	-2.03625	2.8271	-1.64169
H	-2.21656	4.24582	-0.60245
H	-3.6434	3.45262	-1.26986

NHC-5 triplet

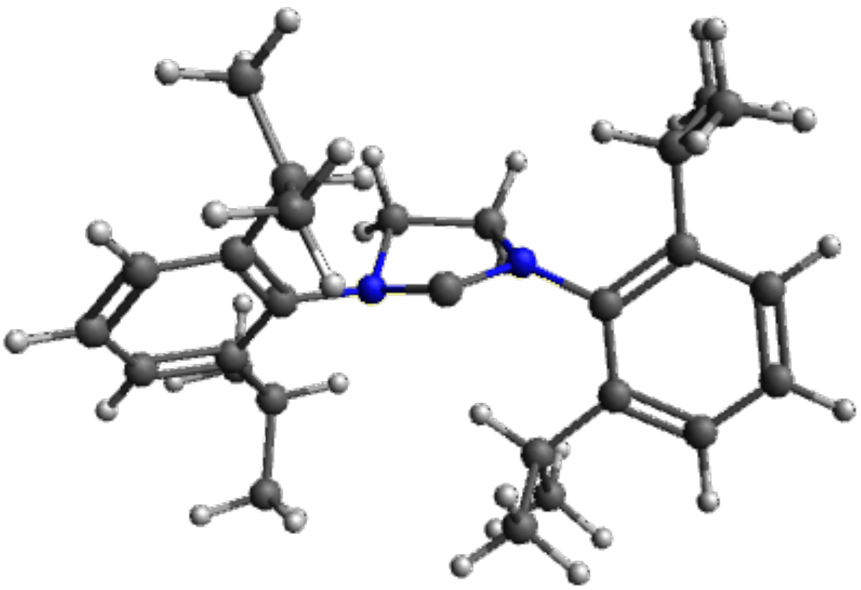

**Zero-point correction=**	**0.588413 (Hartree/Particle)**
**Thermal correction to Energy=**	**0.619992**
**Thermal correction to Enthalpy=**	**0.620936**
**Thermal correction to Gibbs Free Energy=**	**0.522394**
**Sum of electronic and zero-point Energies=**	**-1160.963004**
**Sum of electronic and thermal Energies=**	**-1160.931425**
**Sum of electronic and thermal Enthalpies=**	**-1160.930481**
**Sum of electronic and thermal Free Energies=**	**-1161.029023**

N	-1.24902	0.32586	0.44966	C	2.36882	3.96653	0.07441
N	1.0559	0.10195	0.493	H	2.46168	3.73609	1.13681
C	-0.0714	0.44282	-0.2682	H	1.65346	4.78433	-0.03281
C	-0.86321	0.23854	1.8744	H	3.33907	4.32891	-0.27019
H	-1.08768	-0.7643	2.2473	C	-2.45349	-0.27243	-0.02484
H	-1.39342	0.96384	2.49512	C	-3.6492	0.46734	0.11705
C	0.65426	0.50473	1.86222	C	-4.84061	-0.09148	-0.34075
H	0.86135	1.56733	2.03355	H	-5.76395	0.46302	-0.23329
H	1.18862	-0.08057	2.60868	C	-4.86298	-1.33386	-0.95369
C	2.40611	0.33443	0.07181	H	-5.79546	-1.74702	-1.31558
C	2.82241	1.54703	-0.51958	C	-3.68135	-2.04501	-1.10055
C	4.15633	1.66696	-0.9122	H	-3.70773	-3.01842	-1.57294
H	4.48833	2.58694	-1.37542	C	-2.46648	-1.54819	-0.63254
C	5.06965	0.64698	-0.70595	C	-1.21751	-2.40661	-0.77553
H	6.10091	0.76936	-1.01077	H	-0.40094	-1.90446	-0.26281
C	4.6544	-0.5295	-0.1039	C	-1.38655	-3.78244	-0.11122
H	5.37055	-1.32506	0.05571	H	-1.66719	-3.6846	0.93849
C	3.32916	-0.71354	0.28454	H	-0.45046	-4.34192	-0.16105
C	2.92135	-2.03779	0.91423	H	-2.15366	-4.38164	-0.60404
H	1.84857	-1.99018	1.09304	C	-0.80288	-2.55654	-2.24775
C	3.61496	-2.26561	2.26653	H	-0.63107	-1.58338	-2.7083
H	3.42408	-1.44351	2.95801	H	-1.57316	-3.07043	-2.82601

H	4.69704	-2.35195	2.15096	H	0.11823	-3.1368	-2.32973
H	3.2571	-3.18726	2.72977	C	-3.66598	1.86093	0.72886
C	3.17156	-3.22103	-0.03306	H	-2.63266	2.13572	0.93452
H	2.6581	-3.07895	-0.98442	C	-4.43923	1.89408	2.05664
H	2.80891	-4.14899	0.41347	H	-4.03486	1.17769	2.77331
H	4.23437	-3.35022	-0.245	H	-5.49277	1.64977	1.90776
C	1.89997	2.74164	-0.72617	H	-4.38999	2.88793	2.50619
H	0.91042	2.46668	-0.36147	C	-4.21799	2.90856	-0.25029
C	1.73511	3.08531	-2.21422	H	-3.65391	2.90932	-1.18341
H	1.35045	2.23184	-2.77331	H	-4.15315	3.90734	0.18613
H	2.6827	3.38281	-2.66701	H	-5.26538	2.72214	-0.49359
H	1.03391	3.91283	-2.33759				

NHC-6 singlet

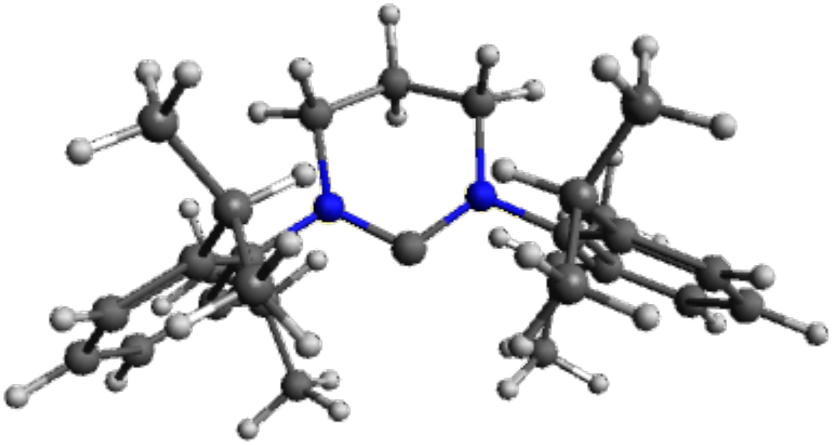

Zero-point correction= **0.619796 (Hartree/Particle)**
Thermal correction to Energy= **0.651932**
Thermal correction to Enthalpy= **0.652876**
Thermal correction to Gibbs Free Energy= **0.554296**
Sum of electronic and zero-point Energies= **-1200.375792**
Sum of electronic and thermal Energies= **-1200.343656**
Sum of electronic and thermal Enthalpies= **-1200.342711**
Sum of electronic and thermal Free Energies= **-1200.441291**

N	-1.14132	0.00275	0.69449	C	3.16388	-3.58381	0.65625
N	1.14075	-0.06178	0.69683	H	2.6151	-4.50447	0.864
C	0.00038	-0.02801	-0.01769	H	3.46363	-3.14666	1.61003
C	-1.24016	0.00432	2.16676	H	4.07319	-3.85859	0.11927
H	-2.15705	-0.51095	2.45215	C	1.85463	-3.25071	-1.48747
H	-1.32343	1.03351	2.53337	H	1.31474	-4.1825	-1.30759
C	-0.02429	-0.68041	2.76905	H	2.71547	-3.48066	-2.11821

H	-0.0198	-0.55992	3.85273
H	-0.06718	-1.75123	2.55991
H	1.41576	0.91799	2.55307
H	2.11326	-0.69075	2.44118
C	-2.39678	0.08667	-0.01096
C	-3.10281	-1.09426	-0.30226
C	-4.33389	-0.98768	-0.94837
H	-4.88862	-1.88516	-1.18937
C	-4.85829	0.24733	-1.29464
H	-5.81555	0.30997	-1.79595
C	-2.55904	-2.4737	0.03789
H	-1.61305	-2.33624	0.55857
C	-3.49632	-3.2527	0.97352
H	-3.04961	-4.21005	1.24905
H	-4.45555	-3.46236	0.49734
H	-3.69891	-2.69806	1.89108
C	-2.25644	-3.28046	-1.23431
H	-1.81296	-4.24502	-0.97914
H	-1.56054	-2.74391	-1.87823
H	-3.16543	-3.47378	-1.80684
C	2.39931	-0.05682	-0.00847
C	2.97874	-1.27884	-0.39394
C	4.21434	-1.2506	-1.03979
H	4.67228	-2.17932	-1.35427
C	4.86638	-0.054	-1.29106
H	5.82592	-0.05286	-1.79192
C	2.29275	-2.61578	-0.15852
H	1.38791	-2.42598	0.41593
H	1.20092	-2.57674	-2.04003
C	1.24287	-0.09321	2.16937
C	-2.91372	1.34692	-0.35679
C	-4.14993	1.40089	-1.00152
H	-4.56261	2.36133	-1.2815
C	-2.16922	2.64221	-0.07157
H	-1.23696	2.38486	0.42821
C	-2.9608	3.56604	0.8672
H	-2.37888	4.45936	1.10264
H	-3.89708	3.89323	0.41176
H	-3.20775	3.0659	1.80492
C	-1.7947	3.36787	-1.3727
H	-1.2109	4.2644	-1.15582
H	-1.20181	2.72201	-2.01948
H	-2.68326	3.67684	-1.92613
C	3.04523	1.16585	-0.25982
C	4.28273	1.14087	-0.9037
H	4.79447	2.07181	-1.11011
C	2.43761	2.50576	0.12738
H	1.46616	2.31005	0.57844
C	3.29513	3.24533	1.16646
H	2.80986	4.17487	1.47077
H	3.45437	2.63842	2.05898
H	4.27653	3.50157	0.76346
C	2.18873	3.38737	-1.10578
H	1.69365	4.31635	-0.81639
H	3.1236	3.65234	-1.60257
H	1.55622	2.87492	-1.82974

NHC-6 triplet

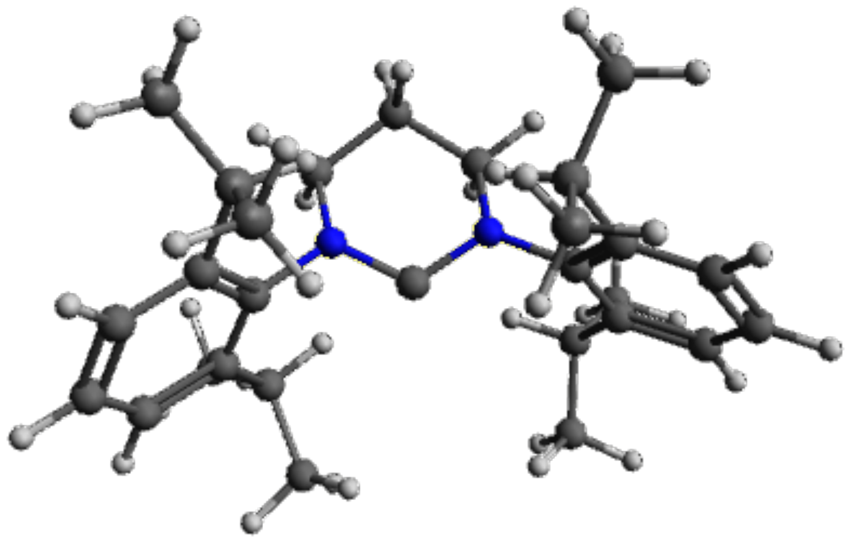

Zero-point correction= **0.617580 (Hartree/Particle)**

**Thermal correction to Energy=**	**0.650004**
**Thermal correction to Enthalpy=**	**0.650948**
**Thermal correction to Gibbs Free Energy=**	**0.550312**
**Sum of electronic and zero-point Energies=**	**-1200.278577**
**Sum of electronic and thermal Energies=**	**-1200.246153**
**Sum of electronic and thermal Enthalpies=**	**-1200.245208**
**Sum of electronic and thermal Free Energies=**	**-1200.345845**

**N**	**-1.17568**	**0.04207**	**0.72877**
**N**	**1.22376**	**-0.0189**	**0.7643**
**C**	**0.0504**	**0.46073**	**0.21656**
**C**	**-1.24185**	**0.27259**	**2.18857**
**H**	**-2.13511**	**-0.22323**	**2.56863**
**H**	**-1.33208**	**1.34523**	**2.41051**
**C**	**0.0135**	**-0.29284**	**2.86148**
**H**	**0.00424**	**-0.02066**	**3.91918**
**H**	**-0.00511**	**-1.38259**	**2.79862**
**H**	**1.4308**	**1.29203**	**2.43679**
**H**	**2.17217**	**-0.30824**	**2.60114**
**C**	**-2.38926**	**0.20671**	**-0.02598**
**C**	**-3.14787**	**-0.94984**	**-0.3036**
**C**	**-4.34689**	**-0.81129**	**-1.00147**
**H**	**-4.94195**	**-1.68916**	**-1.21708**
**C**	**-4.78774**	**0.42947**	**-1.43163**
**H**	**-5.71852**	**0.51743**	**-1.97714**
**C**	**-2.70331**	**-2.33534**	**0.13968**
**H**	**-1.7111**	**-2.22478**	**0.57456**
**C**	**-3.62995**	**-2.90767**	**1.2243**
**H**	**-3.26357**	**-3.87666**	**1.56937**
**H**	**-4.64374**	**-3.05159**	**0.84544**
**H**	**-3.69297**	**-2.24421**	**2.08798**
**C**	**-2.58566**	**-3.30825**	**-1.04264**
**H**	**-2.19409**	**-4.26972**	**-0.70478**
**H**	**-1.9141**	**-2.91984**	**-1.80835**
**H**	**-3.55365**	**-3.49507**	**-1.51106**
**C**	**2.40977**	**-0.2108**	**-0.0195**
**C**	**2.7174**	**-1.51017**	**-0.47014**
**C**	**3.90184**	**-1.7044**	**-1.17888**
**H**	**4.16109**	**-2.69706**	**-1.523**
**C**	**4.75929**	**-0.64833**	**-1.44711**
**H**	**5.67687**	**-0.81989**	**-1.99511**
**C**	**1.80942**	**-2.69484**	**-0.18157**
**H**	**0.88938**	**-2.29507**	**0.24181**
**C**	**2.43221**	**-3.63323**	**0.86459**
**H**	**1.75068**	**-4.45338**	**1.09953**
**H**	**2.65482**	**-3.1031**	**1.79167**
**H**	**3.36498**	**-4.06838**	**0.50038**
**C**	**1.4374**	**-3.46691**	**-1.45516**
**H**	**0.7204**	**-4.25604**	**-1.22266**
**H**	**2.30767**	**-3.94037**	**-1.91301**
**H**	**0.98604**	**-2.80879**	**-2.1981**
**C**	**1.30395**	**0.22411**	**2.21226**
**C**	**-2.83143**	**1.47627**	**-0.45147**
**C**	**-4.03111**	**1.55699**	**-1.16008**
**H**	**-4.38219**	**2.52327**	**-1.49813**
**C**	**-2.06748**	**2.76133**	**-0.16324**
**H**	**-1.16997**	**2.49805**	**0.39462**
**C**	**-2.88908**	**3.73345**	**0.69773**
**H**	**-2.29501**	**4.61555**	**0.94482**
**H**	**-3.78591**	**4.07484**	**0.17764**
**H**	**-3.20634**	**3.2675**	**1.63177**
**C**	**-1.59306**	**3.43965**	**-1.45737**
**H**	**-1.00409**	**4.32934**	**-1.22628**
**H**	**-0.9702**	**2.76563**	**-2.04572**
**H**	**-2.4335**	**3.75066**	**-2.08062**
**C**	**3.25738**	**0.87615**	**-0.31074**
**C**	**4.43352**	**0.62818**	**-1.01989**
**H**	**5.10103**	**1.44922**	**-1.24719**
**C**	**2.92892**	**2.30202**	**0.10824**
**H**	**1.94375**	**2.29249**	**0.57123**
**C**	**3.93369**	**2.84061**	**1.13902**
**H**	**3.64994**	**3.84414**	**1.4621**
**H**	**3.98126**	**2.20209**	**2.02208**
**H**	**4.93983**	**2.89947**	**0.71973**
**C**	**2.8331**	**3.24226**	**-1.10327**
**H**	**2.52922**	**4.24081**	**-0.78323**
**H**	**3.79047**	**3.33782**	**-1.61828**
**H**	**2.09937**	**2.88033**	**-1.82357**

NHC-7 singlet

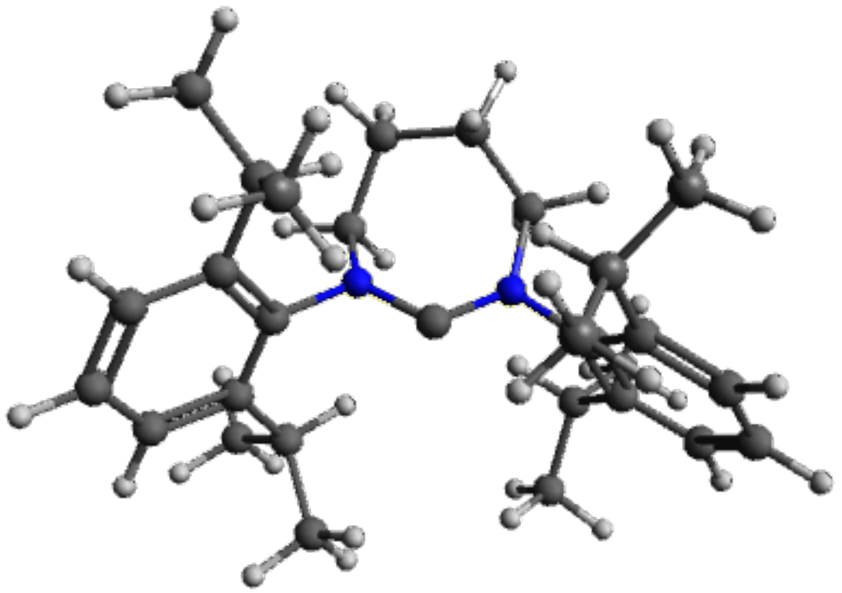

**Zero-point correction=**	**0.648623 (Hartree/Particle)**
**Thermal correction to Energy=**	**0.681790**
**Thermal correction to Enthalpy=**	**0.682734**
**Thermal correction to Gibbs Free Energy=**	**0.583904**
**Sum of electronic and zero-point Energies=**	**-1239.658797**
**Sum of electronic and thermal Energies=**	**-1239.625630**
**Sum of electronic and thermal Enthalpies=**	**-1239.624686**
**Sum of electronic and thermal Free Energies=**	**-1239.723515**

C	0.01006	-0.02613	0.00511	H	1.54591	0.41759	2.71241
N	-1.13238	0.18796	0.68423	H	2.30384	-1.06695	2.19123
C	-1.23938	0.59777	2.10727	H	5.06687	1.62668	-0.87812
C	-0.99814	-0.5216	3.11344	H	5.66871	-0.42678	-2.09012
C	0.26075	-1.33145	2.78178	H	4.16036	-2.36767	-2.04551
C	1.3734	-0.50291	2.14723	H	0.94527	-2.46283	-0.15265
N	1.15088	-0.20429	0.69751	H	0.57189	-3.80192	-2.18274
C	2.3644	-0.26308	-0.0914	H	2.05096	-3.16921	-2.90401
C	3.21313	0.85747	-0.11888	H	0.69979	-2.0802	-2.56842
C	4.40227	0.7732	-0.84394	H	1.82554	-4.75745	-0.18444
C	4.74372	-0.38008	-1.53005	H	3.36337	-4.20442	-0.83989
C	3.89153	-1.47242	-1.5001	H	2.88404	-3.72961	0.78668
C	2.69652	-1.44292	-0.78201	H	1.88989	2.041	1.0456
C	1.80056	-2.67182	-0.79083	H	2.4577	4.24118	0.13127
C	1.24878	-2.94531	-2.19818	H	3.73498	3.54524	-0.86187
C	2.51244	-3.90968	-0.22345	H	2.0527	3.12538	-1.17898
C	2.87367	2.1591	0.59395	H	-5.06915	-1.34412	-1.15263
C	2.77396	3.33511	-0.38968	H	-5.59145	0.85127	-2.12859
C	-2.33361	0.36139	-0.10605	H	-4.02903	2.72822	-1.84076
C	-3.21293	-0.72201	-0.27446	H	-0.82819	2.49813	0.06991
C	-4.38283	-0.51988	-1.0083	H	-0.40216	4.04173	-1.79709
C	-4.67921	0.71375	-1.56251	H	-1.90095	3.54853	-2.583

C	-3.79753 1.76963 -1.39499			H	-0.59128 2.38116 -2.3764		
C	-2.61644 1.61941 -0.66949			H	-1.98588 -2.04988 0.83109		
C	-1.67467 2.8074 -0.53958			H	-2.4619 -4.10911 -0.42596		
C	-1.10943 3.2175 -1.90821			H	-1.93381 -2.82271 -1.51846		
C	-2.92468 -2.10697 0.28483			H	-3.63908 -3.26179 -1.42767		
C	-2.72782 -3.13444 -0.84061			H	3.57498 3.37973 2.25294		
C	3.87027 2.47299 1.721			H	4.8757 2.63293 1.32762		
C	-4.00825 -2.57177 1.26945			H	3.92621 1.65857 2.44454		
C	-2.34136 3.99978 0.16386			H	-3.74258 -3.53886 1.70122		
H	-0.52065 1.404 2.27432			H	-4.13838 -1.86021 2.0863		
H	-2.2277 1.02946 2.25217			H	-4.97527 -2.68614 0.77686		
H	-0.9022 -0.06486 4.10239			H	-1.62458 4.81283 0.29471		
H	-1.86345 -1.18424 3.1626			H	-3.18008 4.39049 -0.4148		
H	0.65124 -1.79406 3.69069			H	-2.72082 3.72228 1.1485		
H	0.01403 -2.15127 2.1057						

NHC-7 triplet

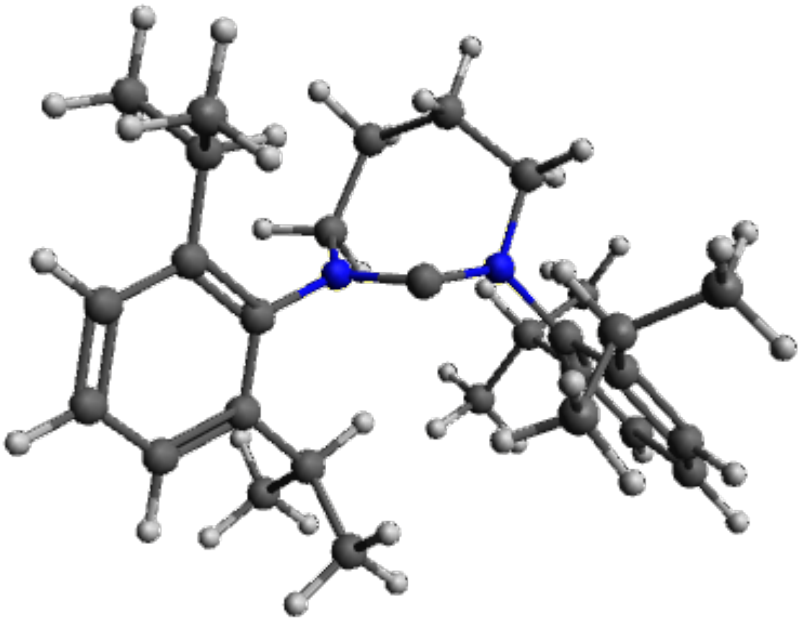

Zero-point correction=	0.646544 (Hartree/Particle)
Thermal correction to Energy=	0.680215
Thermal correction to Enthalpy=	0.681159
Thermal correction to Gibbs Free Energy=	0.579326
Sum of electronic and zero-point Energies=	-1239.576580
Sum of electronic and thermal Energies=	-1239.542909
Sum of electronic and thermal Enthalpies=	-1239.541965
Sum of electronic and thermal Free Energies=	-1239.643798

C	-0.14742 -0.71702 0.00954	H	1.99073 -1.70503 2.28107

N	-1.1798	-0.03515	0.63779
C	-1.05263	0.19244	2.07553
C	-0.80249	-1.08157	2.93024
C	-0.10532	-2.27708	2.25457
C	1.22473	-1.99861	1.55892
N	1.08213	-0.89816	0.58473
C	2.27983	-0.42739	-0.06714
C	3.09321	0.5306	0.57128
C	4.23342	0.98388	-0.09173
C	4.57489	0.50491	-1.34564
C	3.78298	-0.45827	-1.94947
C	2.63612	-0.95158	-1.32814
C	1.8347	-2.04258	-2.02624
C	1.19479	-1.53635	-3.32757
C	2.68808	-3.29444	-2.28601
C	2.78102	1.07871	1.95575
C	2.56644	2.5996	1.94176
C	-2.27136	0.50156	-0.11497
C	-3.50177	-0.18636	-0.15215
C	-4.55648	0.369	-0.87707
C	-4.4053	1.56043	-1.56676
C	-3.18642	2.22033	-1.53794
C	-2.10591	1.71357	-0.81756
C	-0.80451	2.49961	-0.78188
C	-0.20946	2.69648	-2.18356
C	-3.70891	-1.51404	0.55876
C	-4.07953	-2.63605	-0.4233
C	3.86494	0.69431	2.9759
C	-4.74821	-1.4065	1.68545
C	-0.98639	3.84992	-0.06984
H	-0.24135	0.90563	2.25767
H	-1.96941	0.677	2.40945
H	-0.22959	-0.77381	3.80986
H	-1.75911	-1.44715	3.30901
H	0.06987	-3.0453	3.0125
H	-0.78323	-2.719	1.52405
H	1.58087	-2.91443	1.06974
H	4.86334	1.72613	0.38133
H	5.45988	0.87472	-1.84711
H	4.06441	-0.84046	-2.92199
H	1.01743	-2.33314	-1.36882
H	0.60036	-2.3262	-3.79077
H	1.9505	-1.2212	-4.04974
H	0.53677	-0.68968	-3.13426
H	2.07284	-4.08706	-2.71606
H	3.50161	-3.09338	-2.9852
H	3.13052	-3.67313	-1.36348
H	1.84748	0.62543	2.28221
H	2.28633	2.95451	2.93552
H	3.47381	3.12884	1.64631
H	1.77609	2.88225	1.24667
H	-5.50818	-0.14559	-0.91123
H	-5.23419	1.97365	-2.127
H	-3.07674	3.15306	-2.07602
H	-0.08643	1.91756	-0.20791
H	0.75404	3.20516	-2.1191
H	-0.8618	3.30086	-2.81656
H	-0.0517	1.73972	-2.68077
H	-2.75814	-1.78763	1.00982
H	-4.15533	-3.59076	0.10128
H	-3.32558	-2.73668	-1.20425
H	-5.03986	-2.44805	-0.90673
H	3.58764	1.03542	3.97533
H	4.82527	1.14838	2.7257
H	4.01289	-0.38568	3.01537
H	-4.83867	-2.35648	2.21625
H	-4.47343	-0.6388	2.41036
H	-5.73477	-1.15092	1.29442
H	-0.03373	4.37935	-0.00429
H	-1.68569	4.49309	-0.60717
H	-1.37022	3.71709	0.9426

NHC-8 singlet

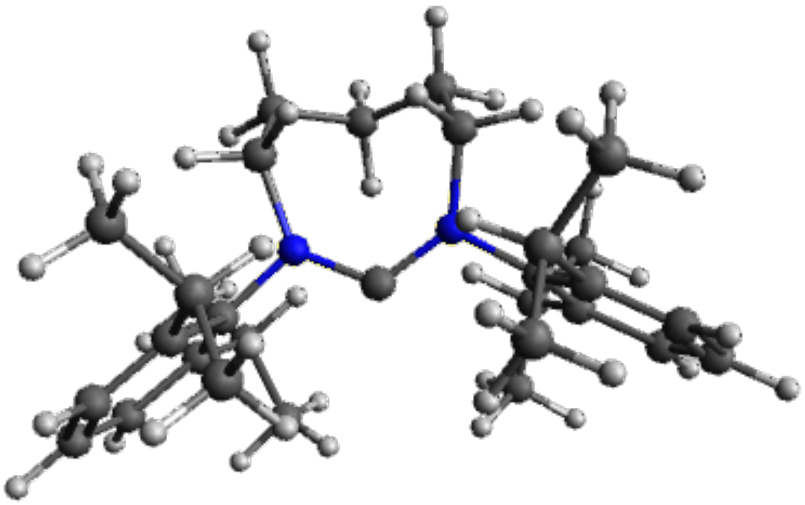

**Zero-point correction=**	**0.677907 (Hartree/Particle)**
**Thermal correction to Energy=**	**0.712030**
**Thermal correction to Enthalpy=**	**0.712974**
**Thermal correction to Gibbs Free Energy=**	**0.612343**
**Sum of electronic and zero-point Energies=**	**-1278.948681**
**Sum of electronic and thermal Energies=**	**-1278.914558**
**Sum of electronic and thermal Enthalpies=**	**-1278.913614**
**Sum of electronic and thermal Free Energies=**	**-1279.014246**

C	0.00018	0.03429	0.02125	C	2.09223	4.20491	0.44565
C	-1.5121	-0.25007	2.12847	C	3.11319	-1.91905	-0.1106
H	-2.5948	-0.14171	2.20112	N	1.16304	0.19414	0.7017
H	-1.09111	0.59981	2.66468	H	2.78726	4.71985	-0.22587
C	-1.11856	-1.5687	2.80822	H	2.64449	3.93783	1.35267
H	-1.34378	-1.45853	3.87838	H	1.31403	4.92479	0.72197
H	-1.77041	-2.36928	2.43768	C	-2.18617	-3.97782	-0.63438
C	0.33901	-2.00116	2.62544	H	-2.70368	-3.95617	0.33065
H	0.51104	-2.90037	3.2297	H	-1.41653	-4.75619	-0.58901
H	0.48839	-2.30917	1.58554	H	-2.91406	-4.28548	-1.3926
C	-2.34042	-0.2854	-0.19314	C	-0.81805	-2.64771	-2.32033
C	-3.27801	0.77302	-0.24479	H	-0.78972	-2.41805	-0.20264
C	-4.43187	0.60952	-1.02047	H	-1.521	-2.80753	-3.14566
H	-5.16016	1.41419	-1.06535	H	-0.08136	-3.45843	-2.34635
C	-4.65621	-0.55544	-1.74532	H	-0.3016	-1.69799	-2.48348
H	-5.55585	-0.66335	-2.34454	C	3.00599	-2.81571	-1.35883
C	-3.71345	-1.57585	-1.70589	H	2.82909	-3.85745	-1.06791
H	-3.88226	-2.47834	-2.2858	H	3.92675	-2.79096	-1.95166
C	-2.55025	-1.47042	-0.93195	H	2.18364	-2.49499	-2.00288
C	-3.07119	2.10063	0.48292	C	4.26203	-2.39403	0.79999
C	-1.54627	-2.61688	-0.96327	H	2.17891	-2.02549	0.44258
N	-1.17634	-0.15285	0.67133	H	4.35177	-1.77309	1.69714
C	1.4231	0.34095	2.16809	H	5.22512	-2.35613	0.27951
H	2.41195	0.79575	2.25926	H	4.09947	-3.4301	1.11754
H	0.72629	1.0727	2.57973	C	-4.12475	2.3331	1.58425

C	1.40963	-0.96562	2.99308	H	-5.13223	2.39663	1.15914
H	1.31393	-0.68757	4.05188	H	-3.92836	3.27272	2.11252
H	2.38759	-1.44912	2.8963	H	-4.13155	1.52646	2.32389
C	2.31058	0.53429	-0.13173	C	-3.06259	3.28553	-0.5028
C	2.48327	1.87943	-0.53133	H	-2.08634	2.07364	0.95723
C	3.61106	2.21442	-1.28929	H	-2.32483	3.13522	-1.29448
H	3.7482	3.24221	-1.61221	H	-2.81956	4.21659	0.02164
C	4.55688	1.25698	-1.64033	H	-4.04046	3.42153	-0.97676
H	5.4284	1.53671	-2.22543	C	0.68179	3.34354	-1.49086
C	4.37335	-0.06139	-1.24289	H	0.73604	2.55502	0.49072
H	5.10743	-0.81043	-1.52567	H	0.17681	2.46141	-1.8945
C	3.25735	-0.4496	-0.4886	H	1.35264	3.73851	-2.26185
C	1.46073	2.9666	-0.21612	H	-0.06928	4.111	-1.2745

NHC-8 triplet

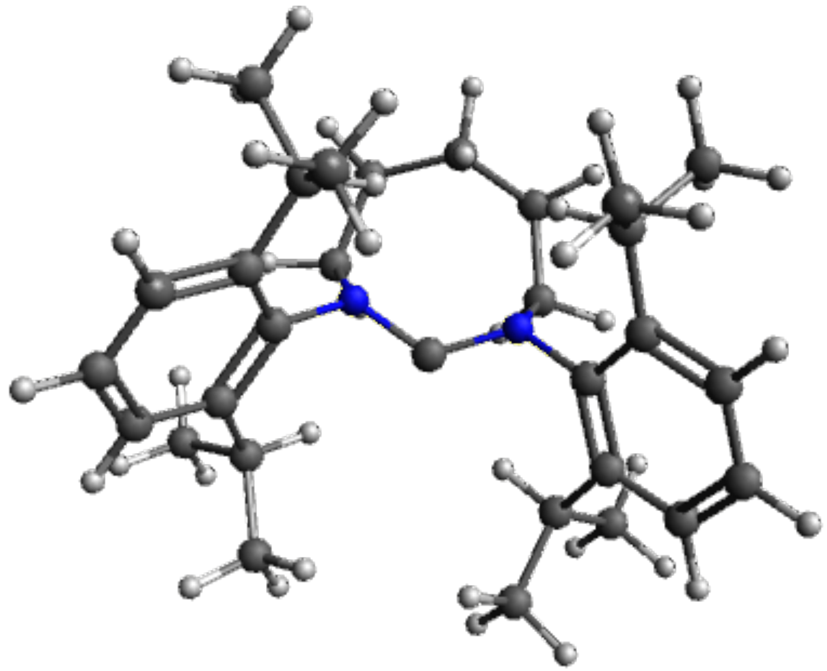

Zero-point correction= 0.675606 (Hartree/Particle)
Thermal correction to Energy= 0.710025
Thermal correction to Enthalpy= 0.710969
Thermal correction to Gibbs Free Energy= 0.607825
Sum of electronic and zero-point Energies= -1278.867915
Sum of electronic and thermal Energies= -1278.833496
Sum of electronic and thermal Enthalpies= -1278.832552
Sum of electronic and thermal Free Energies= -1278.935696

C	-0.04524	0.51846	0.4461	C	3.08563	3.5668	0.90112

C	-1.67035	0.05794	2.18989
H	-2.75779	0.17586	2.22291
H	-1.23546	0.98813	2.57794
C	-1.27332	-1.12699	3.09311
H	-1.36988	-0.79336	4.13616
H	-2.01285	-1.92647	2.96197
C	0.10934	-1.75741	2.86451
H	0.17348	-2.63821	3.5159
H	0.1466	-2.13745	1.83958
C	-2.32058	-0.06093	-0.22109
C	-3.01486	1.1445	-0.49031
C	-4.08091	1.11242	-1.39814
H	-4.62654	2.02787	-1.60822
C	-4.45374	-0.06481	-2.03713
H	-5.28582	-0.06793	-2.73563
C	-3.75073	-1.2365	-1.78057
H	-4.04274	-2.15365	-2.28344
C	-2.67752	-1.25921	-0.8809
C	-2.65819	2.47533	0.16936
C	-1.9547	-2.57537	-0.62111
N	-1.2601	-0.0952	0.76903
C	1.62133	0.29387	2.19171
H	2.68498	0.55079	2.22497
H	1.07054	1.16897	2.56057
C	1.37305	-0.91802	3.11566
H	1.38456	-0.55232	4.15219
H	2.22687	-1.60043	3.02783
C	2.29173	0.2483	-0.21453
C	2.71516	1.54797	-0.58836
C	3.7675	1.67008	-1.50459
H	4.10365	2.66076	-1.79664
C	4.39423	0.55218	-2.04342
H	5.21044	0.67033	-2.75063
C	3.97036	-0.71742	-1.66895
H	4.46524	-1.58908	-2.08689
C	2.92072	-0.8959	-0.75869
C	2.09653	2.8226	-0.01782
C	2.52446	-2.31212	-0.35897
N	1.24051	0.06823	0.77104
H	3.97288	3.89539	0.34853
H	3.42623	2.93206	1.72513
H	2.61395	4.45666	1.3328
C	-2.82131	-3.52847	0.22517
H	-3.11495	-3.06709	1.17301
H	-2.27611	-4.45192	0.45072
H	-3.73928	-3.80335	-0.30659
C	-1.51097	-3.26412	-1.92418
H	-1.05702	-2.33795	-0.04489
H	-2.36603	-3.60119	-2.52009
H	-0.90298	-4.14681	-1.70011
H	-0.91595	-2.5905	-2.54775
C	2.25265	-3.21378	-1.57645
H	1.87113	-4.18742	-1.24989
H	3.16236	-3.40069	-2.1573
H	1.51398	-2.76742	-2.24733
C	3.59243	-2.94972	0.55202
H	1.59728	-2.23361	0.21369
H	3.78194	-2.3378	1.4392
H	4.5447	-3.06379	0.02185
H	3.27456	-3.944	0.88562
C	-3.77001	2.95742	1.1223
H	-4.70363	3.14502	0.58056
H	-3.47702	3.89244	1.61265
H	-3.9841	2.22043	1.90237
C	-2.33988	3.55977	-0.87759
H	-1.74605	2.3279	0.7533
H	-1.5497	3.23052	-1.5575
H	-2.00352	4.47811	-0.3838
H	-3.21783	3.81416	-1.48111
C	1.58194	3.75573	-1.1297
H	1.22585	2.53659	0.57848
H	0.88264	3.23315	-1.78837
H	2.39813	4.14426	-1.74834
H	1.06202	4.61609	-0.69461

NHC-9 saturated singlet

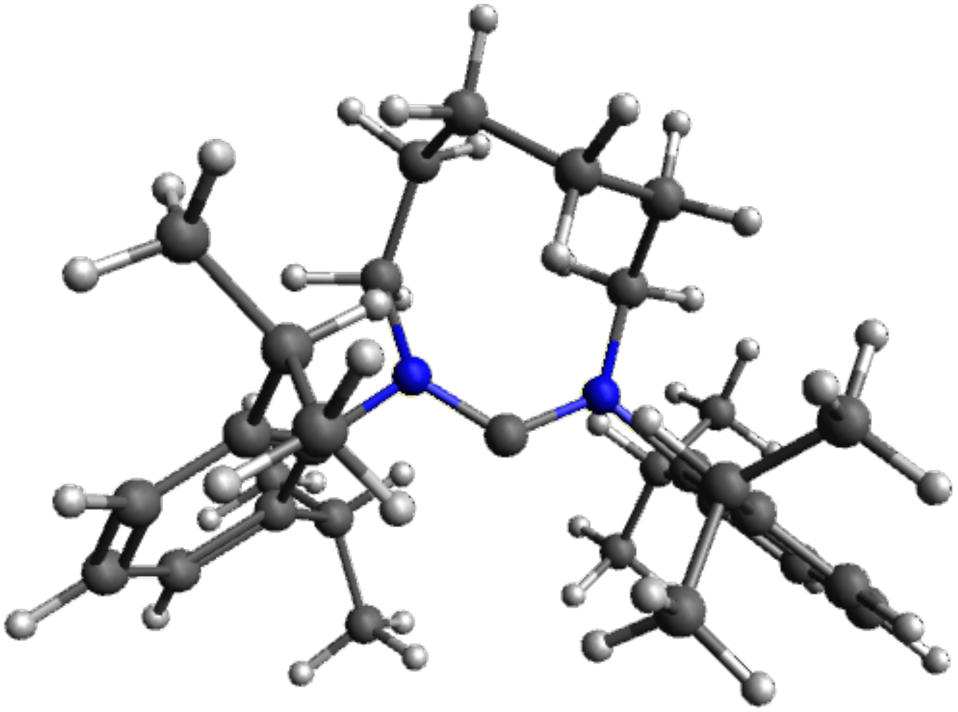

Zero-point correction=	0.751461 (Hartree/Particle)
Thermal correction to Energy=	0.791615
Thermal correction to Enthalpy=	0.792559
Thermal correction to Gibbs Free Energy=	0.678053
Sum of electronic and zero-point Energies=	-1623.080476
Sum of electronic and thermal Energies=	-1623.040322
Sum of electronic and thermal Enthalpies=	-1623.039378
Sum of electronic and thermal Free Energies=	-1623.153885

N	1.17354	-0.20428	0.59429
C	1.46247	-0.48508	2.02442
C	1.23499	-1.94610	2.43796
H	0.89654	0.20218	2.63922
H	2.50989	-0.24923	2.19362
C	-0.14487	-2.52947	2.12533
H	1.44503	-2.02371	3.51030
H	1.99015	-2.55797	1.93944
C	-1.31144	-2.00719	2.97394
H	-0.35518	-2.36874	1.06978
H	-0.09679	-3.61382	2.25270
C	-1.35823	-0.49967	3.24399
H	-1.26972	-2.48926	3.95549
H	-2.24972	-2.33666	2.52448
C	-1.50076	0.52511	2.08447
H	-0.49341	-0.24059	3.85350
H	-2.21653	-0.31493	3.89495
C	0.00917	0.05898	-0.01893
H	-2.25966	-1.91471	0.33100
C	-1.31458	3.03904	-0.24821
C	-0.64655	3.65480	-1.48697
H	-0.17055	2.88366	-2.09202
H	0.11455	4.37810	-1.18914
H	-1.37013	4.17838	-2.11375
C	-1.92963	4.13065	0.64252
H	-1.16469	4.84480	0.95421
H	-2.38310	3.70762	1.54000
H	-2.70494	4.68711	0.11294
H	-0.52774	2.55068	0.32288
C	2.54182	-1.32231	-1.16352
C	3.30187	0.77395	-0.15373
C	4.46057	0.69806	-0.92487
C	4.67578	-0.35407	-1.79888
C	3.71580	-1.34334	-1.91867
H	5.20072	1.48393	-0.84973
H	5.58091	-0.39642	-2.39072

N	-1.15541	0.22616	0.64459
H	-0.96721	1.43477	2.37219
H	-2.54883	0.80516	2.03475
C	2.34626	-0.25794	-0.26373
C	-2.24371	0.63458	-0.24280
C	-3.20672	-0.30226	-0.65669
C	-4.26065	0.13415	-1.46145
C	-4.37278	1.45729	-1.84965
C	-3.41248	2.36803	-1.44113
C	-2.33839	1.98288	-0.64016
H	-5.00444	-0.57924	-1.79167
H	-5.19914	1.77665	-2.47135
H	-3.49653	3.40050	-1.75412
C	-3.14169	-1.77452	-0.28613
C	-4.36349	-2.21643	0.53438
H	-4.26241	-3.25961	0.84112
H	-5.28475	-2.13195	-0.04446
H	-4.48425	-1.60961	1.43301
C	-2.96897	-2.66252	-1.52796
H	-3.83608	-2.60012	-2.18779
H	-2.85053	-3.70811	-1.23575
H	-2.09145	-2.36588	-2.10100
H	3.87870	-2.15480	-2.61558
C	1.52945	-2.43551	-1.38296
C	2.11984	-3.82574	-1.10240
H	1.34771	-4.59109	-1.20234
H	2.53093	-3.89106	-0.09390
H	2.92047	-4.07512	-1.80075
C	0.93583	-2.36328	-2.79824
H	0.70748	-2.27821	-0.69183
H	0.18666	-3.14525	-2.93742
H	0.45988	-1.39728	-2.96276
H	1.70431	-2.50180	-3.56108
C	3.10732	1.99648	0.73642
C	3.05800	3.28771	-0.09628
H	2.29338	3.22782	-0.86921
H	4.01327	3.48273	-0.58602
H	2.83339	4.14422	0.54282
C	4.18520	2.10558	1.82703
H	5.17513	2.25038	1.39140
H	4.22925	1.21011	2.44815
H	3.98388	2.95783	2.47907
H	2.14196	1.90440	1.23072

NHC-9 saturated triplet

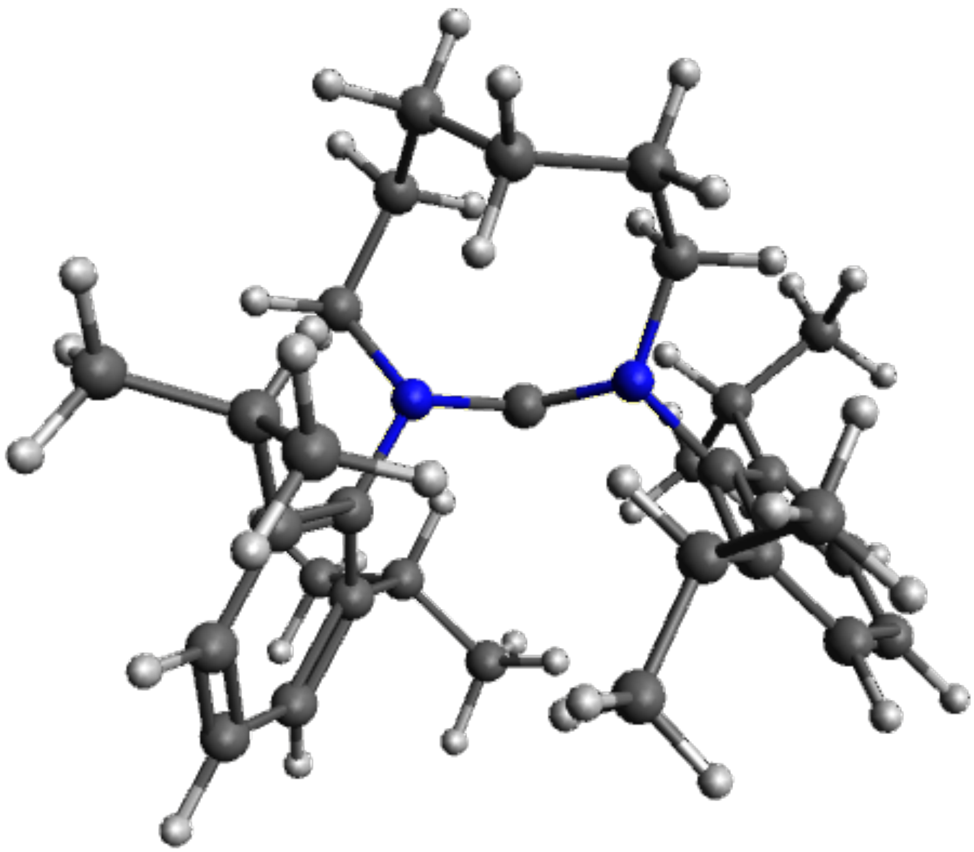

Zero-point correction=	0.751461 (Hartree/Particle)
Thermal correction to Energy=	0.791615
Thermal correction to Enthalpy=	0.792559
Thermal correction to Gibbs Free Energy=	0.678053
Sum of electronic and zero-point Energies=	-1623.080476
Sum of electronic and thermal Energies=	-1623.040322
Sum of electronic and thermal Enthalpies=	-1623.039378
Sum of electronic and thermal Free Energies=	-1623.153885

N	1.09677	-0.95886	0.30405	H	-2.86515	-1.56769	0.30271
C	1.53078	-2.17287	1.03902	C	-0.46432	2.88101	1.06031
C	0.64018	-3.40709	0.87281	C	0.60495	3.57942	0.20816
H	1.64080	-1.93356	2.09895	H	1.08317	2.87846	-0.47569
H	2.52521	-2.42686	0.67347	H	1.37788	4.01044	0.84705
C	-0.85792	-3.25405	1.14778	H	0.18169	4.39268	-0.38379
H	1.05699	-4.18549	1.52231	C	-1.06708	3.85420	2.08768
H	0.75027	-3.77488	-0.15097	H	-0.28442	4.24889	2.73874
C	-1.30975	-2.90601	2.57645	H	-1.81102	3.36060	2.71459
H	-1.25617	-2.52717	0.44545	H	-1.55395	4.70231	1.60267
H	-1.32614	-4.20878	0.89073	H	0.03905	2.08923	1.61202
C	-1.04282	-1.49743	3.13504	C	1.84606	-0.40503	-1.96775
H	-0.86219	-3.62726	3.26858	C	3.26176	0.21341	-0.06553
H	-2.38886	-3.08388	2.62913	C	4.19572	0.72618	-0.96531

**C**	**-1.68026**	**-0.31464**	**2.40666**
**H**	**0.02691**	**-1.31119**	**3.22966**
**H**	**-1.43422**	**-1.47217**	**4.15644**
**C**	**0.25605**	**-0.05893**	**0.94964**
**N**	**-1.12857**	**-0.06335**	**1.05507**
**H**	**-1.56116**	**0.58219**	**3.02842**
**H**	**-2.75390**	**-0.47521**	**2.29484**
**C**	**2.08296**	**-0.36894**	**-0.58016**
**C**	**-1.89537**	**0.87423**	**0.25088**
**C**	**-3.01891**	**0.40175**	**-0.46474**
**C**	**-3.72645**	**1.29155**	**-1.27160**
**C**	**-3.36185**	**2.62270**	**-1.37228**
**C**	**-2.29288**	**3.08803**	**-0.62730**
**C**	**-1.55397**	**2.24550**	**0.20481**
**H**	**-4.57899**	**0.93216**	**-1.83304**
**H**	**-3.91703**	**3.29585**	**-2.01254**
**H**	**-2.03255**	**4.13696**	**-0.67811**
**C**	**-3.52094**	**-1.03245**	**-0.37521**
**C**	**-4.93973**	**-1.09817**	**0.21467**
**H**	**-5.24887**	**-2.13696**	**0.34670**
**H**	**-5.66871**	**-0.61714**	**-0.43949**
**H**	**-4.99341**	**-0.60316**	**1.18532**
**C**	**-3.46471**	**-1.75976**	**-1.72637**
**H**	**-4.11156**	**-1.28371**	**-2.46516**
**H**	**-3.79463**	**-2.79456**	**-1.61515**
**H**	**-2.45223**	**-1.76966**	**-2.12842**
**C**	**3.97961**	**0.68413**	**-2.33327**
**C**	**2.81112**	**0.12672**	**-2.82394**
**H**	**5.10713**	**1.17150**	**-0.58813**
**H**	**4.71823**	**1.08829**	**-3.01349**
**H**	**2.64581**	**0.09865**	**-3.89300**
**C**	**0.58992**	**-1.02109**	**-2.56313**
**C**	**0.91247**	**-2.30559**	**-3.34302**
**H**	**-0.00365**	**-2.76680**	**-3.71766**
**H**	**1.42481**	**-3.03441**	**-2.71334**
**H**	**1.55547**	**-2.10006**	**-4.20106**
**C**	**-0.17839**	**-0.02347**	**-3.44192**
**H**	**-0.05573**	**-1.29556**	**-1.73116**
**H**	**-1.09801**	**-0.47618**	**-3.81645**
**H**	**-0.44995**	**0.87054**	**-2.88072**
**H**	**0.40921**	**0.28735**	**-4.30761**
**C**	**3.56183**	**0.30308**	**1.42626**
**C**	**3.80112**	**1.75033**	**1.88304**
**H**	**2.95286**	**2.38610**	**1.63472**
**H**	**4.69020**	**2.18045**	**1.41918**
**H**	**3.94585**	**1.78362**	**2.96472**
**C**	**4.75460**	**-0.58356**	**1.82180**
**H**	**5.67326**	**-0.24676**	**1.33794**
**H**	**4.59684**	**-1.62471**	**1.53956**
**H**	**4.91587**	**-0.54757**	**2.90107**
**H**	**2.68508**	**-0.04843**	**1.96519**

1 A. J. Arduengo, R. Krafczyk, R. Schmutzler, H. A. Craig, J. R. Goerlich, W. J. Marshall and M. Unverzagt, *Tetrahedron* 1999, **55**, 14523.

2 M. Iglesias, D. J. Beetstra, J. C. Knight, L.-L. Ooi, A. Stasch, S. Coles, L. Male, M. B. Hursthouse, K. J. Cavell, A. Dervisi and I. A. Fallis, *Organometallics*, 2008, **27**, 3279.

3 E. L. Kolychev, I. A. Portnyagin, V. V. Shuntikov, V. N. Khrustalev and M. S. Nechaev, *J. Organomet. Chem.* 2009, **694**, 2454.

4 W. Y. Lu, K. J. Cavell, J. S. Wixey and B. Kariuki, *Organometallics*, 2011, **30**, 5649.

5 O.V. Dolomanov, L. J. Bourhis, R. J. Gildea, J. A. K. Howard, H. Puschmann. *J. Appl. Cryst.* 2009, **42**, 339–341.

6 G. M. Sheldrick. *Acta Crystallogr. A.* 2008, **64**, 112–122.

7 Gaussian 16, Revision C.01, Frisch, M. J.; Trucks, G. W.; Schlegel, H. B.; Scuseria, G. E.; Robb, M. A.; Cheeseman, J. R.; Scalmani, G.; Barone, V.; Petersson, G. A.; Nakatsuji, H.; Li, X.; Caricato, M.; Marenich, A. V.; Bloino, J.; Janesko, B. G.; Gomperts, R.; Mennucci, B.; Hratchian, H. P.; Ortiz, J. V.; Izmaylov, A. F.; Sonnenberg, J. L.; Williams-Young, D.; Ding, F.; Lipparini, F.; Egidi, F.; Goings, J.; Peng, B.; Petrone, A.; Henderson, T.; Ranasinghe, D.; Zakrzewski, V. G.; Gao, J.; Rega, N.; Zheng, G.; Liang, W.; Hada, M.; Ehara, M.; Toyota, K.; Fukuda, R.; Hasegawa, J.; Ishida, M.; Nakajima, T.; Honda, Y.; Kitao, O.; Nakai, H.; Vreven, T.; Throssell, K.; Montgomery, J. A., Jr.; Peralta, J. E.; Ogliaro, F.; Bearpark, M. J.; Heyd, J. J.; Brothers, E. N.; Kudin, K. N.; Staroverov, V. N.; Keith, T. A.;

Kobayashi, R.; Normand, J.; Raghavachari, K.; Rendell,A. P.; Burant, J. C.; Iyengar, S. S.; Tomasi, J.; Cossi, M.; Millam, J. M.; Klene, M.; Adamo, C.; Cammi, R.; Ochterski, J. W.; Martin, R. L.; Morokuma, K.; Farkas, O.; Foresman, J. B.; Fox, D. J. Gaussian, Inc., Wallingford CT, 2016.

8 F. Weigend and R. Ahlrichs. *Phys. Chem. Chem. Phys.* 2005, **7**, 3297-3305.

9 L. Falivene, Z. Cao, A. Petta, L. Serra, A. Poater, R. Oliva, V. Scarano and L. Cavallo. *Nat. Chem.* 2019, **11**, 872 – 879.
